# ENaC regulation by phospholipids and DGK explained through mathematical modeling

**DOI:** 10.1038/s41598-020-70630-w

**Published:** 2020-08-18

**Authors:** Daniel V. Olivença, Eberhard O. Voit, Francisco R. Pinto

**Affiliations:** 1 Faculty of Sciences, BioISI – Biosystems and Integrative Sciences Institute, University of Lisboa, 1749-016 Lisbon, Portugal; 2 The Wallace H. Coulter Department of Biomedical Engineering, Georgia Institute of Technology and Emory University, 950 Atlantic Drive, Atlanta, GA 30332-2000 USA

**Keywords:** Computational models, Computer modelling, Respiration, Cystic fibrosis

## Abstract

Cystic fibrosis is a condition caused by mutations in the cystic fibrosis transmembrane conductance regulator (CFTR). It is also thought to increase the activity of epithelial sodium channels (ENaC). The altered function of these ion channels is one of the causes of the thick dehydrated mucus that characterizes the disease and is partially responsible for recurrent pulmonary infections and inflammation events that ultimately destroy the lungs of affected subjects. Phosphoinositides are signaling lipids that regulate numerous cellular processes and membrane proteins, including ENaC. Inhibition of diacylglycerol kinase (DGK), an enzyme of the phosphoinositide pathway, reduces ENaC function. We propose a computational analysis that is based on the combination of two existing mathematical models: one representing the dynamics of phosphoinositides and the other explaining how phosphatidylinositol 4,5-bisphosphate (PI(4,5)P_2_) influences ENaC activity and, consequently, airway surface liquid. This integrated model permits, for the first time, a detailed assessment of the intricate interactions between DGK and ENaC and is consistent with available literature data. In particular, the computational approach allows comparisons of two competing hypotheses regarding the regulation of ENaC. The results strongly suggest that the regulation of ENaC is primarily exerted through the control of PI(4,5)P_2_ production by type-I phosphatidylinositol-4-phosphate 5-kinase (PIP5KI), which in turn is controlled by phosphatidic acid (PA), the product of the DGK reaction.

## Introduction

Mutations in the cystic fibrosis transmembrane conductance regulator (CFTR), a chloride and bicarbonate membrane channel, can cause problems in several organs and lead to cystic fibrosis (CF). In the lungs, the production of thick, dehydrated mucus associated with these mutations leads to recurrent infections and frequent inflammation events that eventually compromise organ function. Life expectancy for subjects with CF has improved considerably^1^, and promising new drugs like lumacaftor and ivacaftor that correct the mutated protein were recently brought to the market^2^. Despite these advancements, a complete cure for CF has not yet been achieved, in part due to mutations that are not treatable with the available drugs.

While CF is directly caused by mutations in the CFTR protein, other ion channels, like the Epithelium Sodium Channel (ENaC), are affected as well. It has been hypothesized that the lack of CFTR in CF lungs causes ENaC function to increase. Consequently, large amounts of sodium and water are absorbed, implying that this channel may contribute to the thick dehydrated mucus that characterizes the disease.

The reasons for ENaC’s upregulation in CF are not clear, but there is no shortage of hypotheses^3–9^. Among these, Tarran and colleagues^10–12^ advanced the idea that short palate lung and nasal epithelial clone 1 (SPLUNC1) is deactivated in the absence of CFTR, either by increased acidity in the specific environment of the CF lung or due to an increased presence of proteases^13^. The inactive SPLUNC1 does not fulfill its role of inducing the channel disassembly and removal of ENaC α and γ subunits from the plasma membrane^9^. As a consequence, ENaC channels have a lower probability of being removed from the plasma membrane and, ultimately, ENaC function is up-regulated. We adopted this view because it was at least partially validated by results obtained under physiologically and clinically relevant conditions^14^.

Phosphoinositides are relatively rare membrane lipids with various signaling functions. They are characteristic for different types of cell membranes and play a dynamic role in cell process control as second messengers and precursors of other messengers. As a result, phosphoinositides are important in a myriad of cell functions like cytoskeleton formation, chemotaxis, cell polarization, T cell activation and cytokinesis^15,16^. Here, we are particularly interested in their function as docking sites for proteins to the cell membrane and as membrane protein regulators.

**Figure 1:**
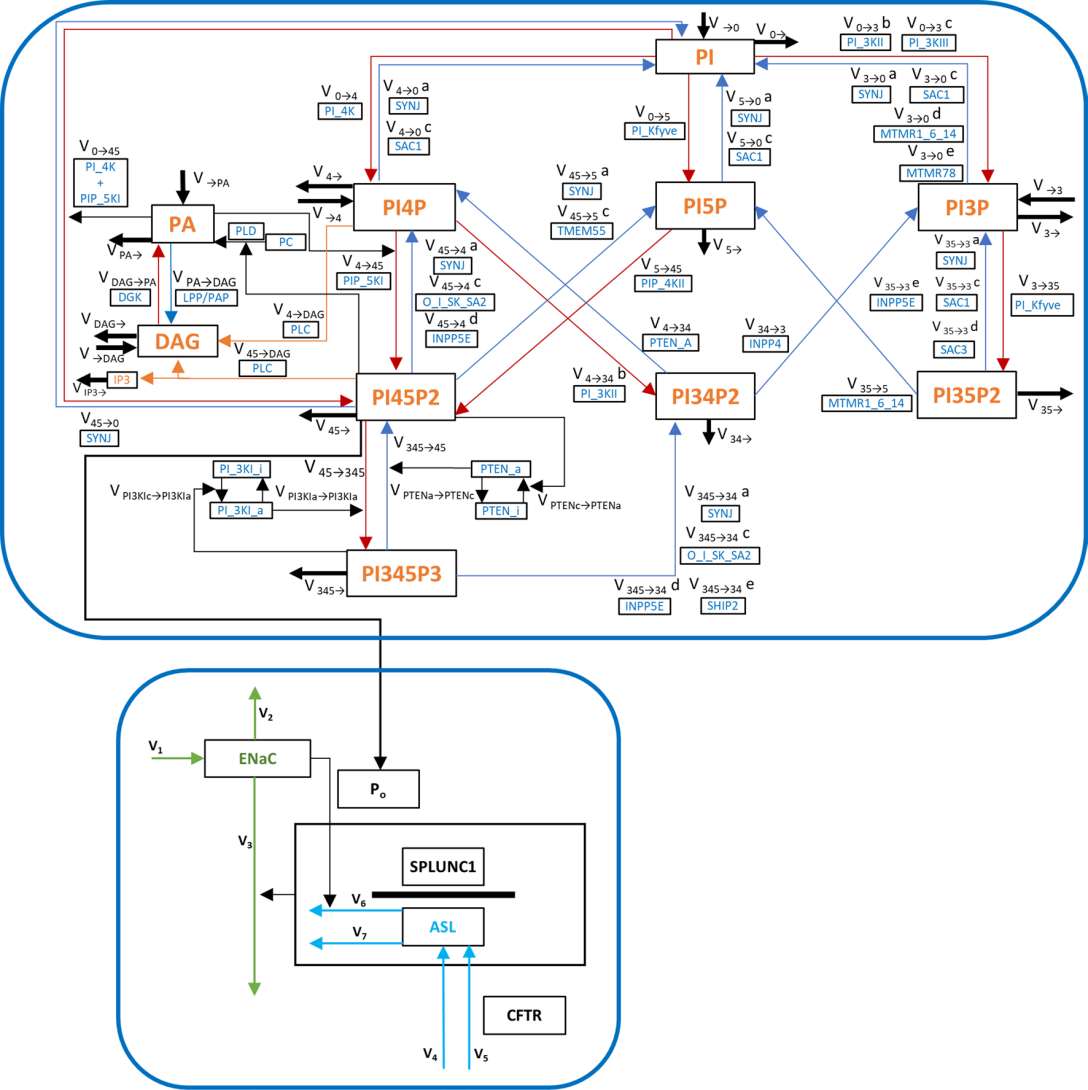
Diagram of the integrated model. Upper box: Phosphoinositide model: Many components were already represented in our previous model^24^, while the components associated with PA and DAG (left) are new extensions. The arrow from PI(4,5)P_2_ to ENaC represents the control that the lipid exerts over the channel and constitutes the link between the two component models. Thick black arrows represent influxes and effluxes of material entering and leaving the system. Red and blue arrows represent fluxes of phosphorylation and hydrolysis, respectively. Thin black arrows represent regulations. For each flux, the name (v_i→j_) and the group of enzymes that catalyze the reaction are shown. Orange arrows represent phospholipase fluxes. PTEN and PI3KI have an active (a) and inactive (i) state. O_I_SK_SA2 is a group of phosphatases, consisting of OCRL1, INPP5 B/J, SAC2 and SKIP. PI4K + PIP5KI + DVL denotes a complex formed by the three proteins. Proteins separated by commas catalyze the same reaction. Lower box: ENaC—ASL model diagram. Green arrows represent influx and efflux of ENaC channels. Cyan arrows represent influxes and effluxes of material for ASL. Black arrows depict regulation. The value of PI(4,5)P2 regulates the open probability of ENaC. For equations and parameter values please see our previous paper^29^. Abbreviations: V_1_—ENaC influx. V_2_—ASL independent ENaC efflux. V_3_—ASL dependent ENaC efflux. V_4_—CFTR independent ASL influx. V_5_—CFTR dependent ASL influx. V_6_—ENaC dependent ASL efflux. V_7_—ENaC independent ASL efflux.

Several studies have shown that two of these lipids, phosphatidylinositol 4,5-bisphosphate (PI(4,5)P_2_) and phosphatidylinositol 3,4,5-triphosphate (PI(3,4,5)P_3_), have an effect on ENaC^17–19^. Their key precursor, phosphatidylinositol (PI), is created in the ER from phosphatidic acid (PA) and transported to the plasma membrane, where it is phosphorylated into other phosphoinositide species (Fig. 1, upper box). PI(4,5)P_2_ is cleaved by phospholipase C (PLC) into inositol triphosphate (IP3) and diacylglycerol (DAG) and transformed into PA, which is transported back to the ER, closing a functional cycle.

Kota et al.^20^ offered an explanation linking phosphoinositides to ENaC control. They found that when the intracellular N-termini of ENaC connect to phosphoinositides, a conformational change occurs that exposes the extracellular loops of the channel to proteases. Severing these loops leads to an increase in the probability P_o_ that ENaC is open. Thus, if the levels of PI(4,5)P_2_ or PI(3,4,5)P_3_ are decreased, proteases do not cut ENaC’s extracellular loops as often, and the channel activity is reduced.

Diacylglycerol kinase (DGK) transforms DAG into PA. Almaça et al.^21^ found that inhibiting DGK causes a moderation of ENaC activity and normalizes the increased sodium channel activity in CF. The authors hypothesized that inhibiting DGK might bring the recycling of phosphoinositides to a halt, which in turn would decrease the levels of PI(4,5)P_2_ and PI(3,4,5)P_3_ and cause the observed ENaC moderation. However, DGK is active in the plasma membrane, while phosphoinositide synthesis occurs in the ER. Transport of lipids between membranes of different compartments is typically mediated by vesicles or specialized proteins, but quantitative details about the dynamics of this transport in the CF lung, if it occurs, are lacking. Thus, many open questions remain unanswered. Crucially, the dynamics of the control of ENaC by DGK is still unclear, and it remains to be explored whether and how DGK could potentially be used as a therapy in situations where ENaC function is dysregulated.

**Figure 2:**
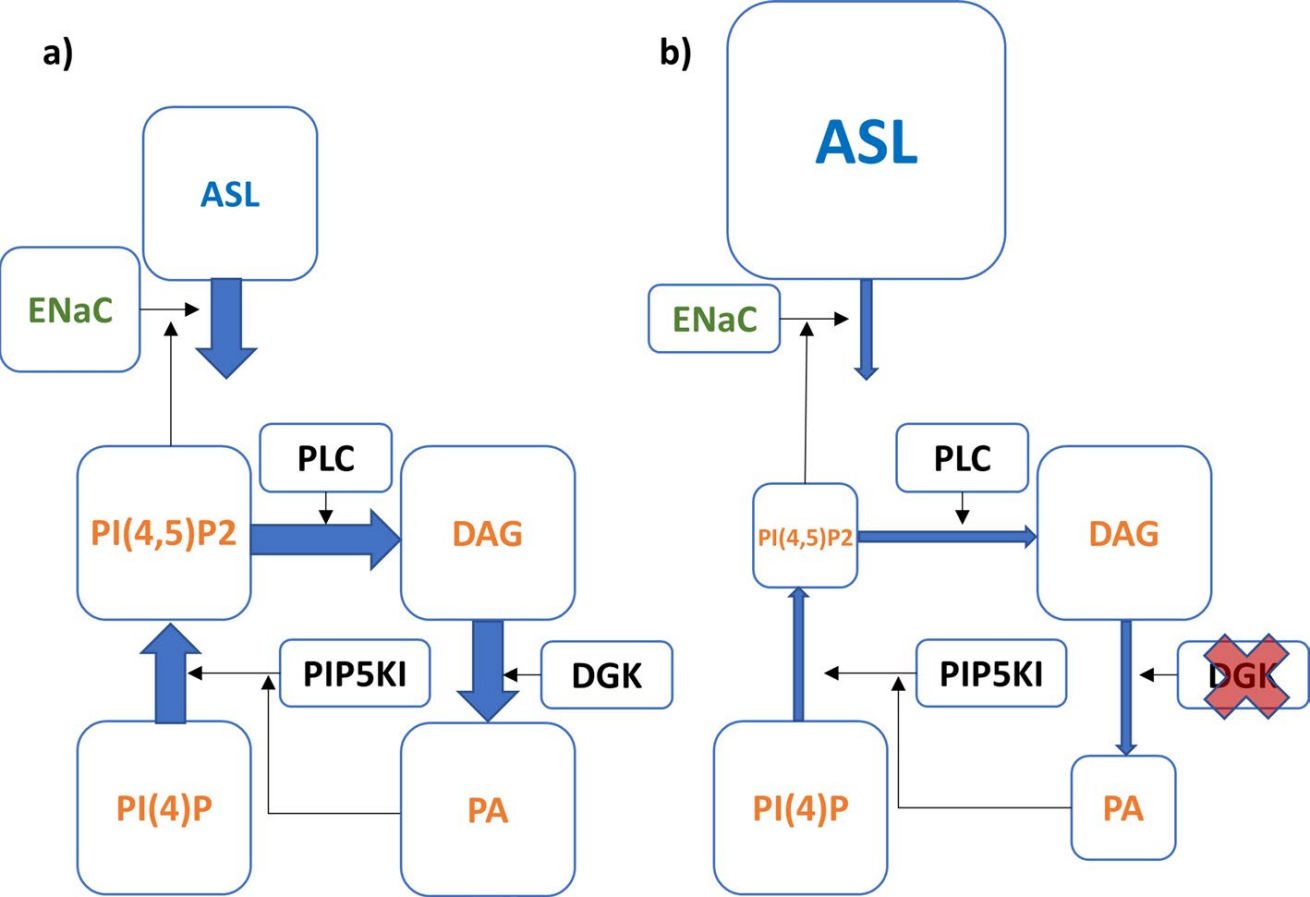
Consequences of inhibiting DGK for phosphoinositide metabolism, ENaC and ASL, visualized with sizes of boxes and arrows. Panel a: Normal state. Panel b: DGK inhibition reduces the production of PA which, in turn, reduces the production of PI(4,5)P_2_, which is catalyzed by PIP5KI. Low levels of PI(4,5)P_2_ reduce ENaC activity and the absorption of ASL stimulated by this channel, which consequently leads to an increase in ASL thickness.

In this work, we investigate ENaC control by DGK. As an alternative to Almaça’s hypothesis, we proffer that ENaC is regulated by PI(4,5)P_2_, which is produced by type-I phosphatidylinositol-4-phosphate 5-kinase (PIP5KI) under the control of PA, which in turn is produced from DAG under the control of DGK (Fig. 2).

We furthermore suggest that PI(4,5)P_2_ specifically influences the probability P_o_ that ENaC is open (“open-probability”) rather than the number N of channels in the membrane, in agreement with Ma et al.^22^. N itself is influenced by ubiquitination by NEDD4-2, the ***N***eural precursor cell ***E***xpressed ***D***evelopmentally ***D***own-regulated protein **4–2**^23^ and an interaction with SPLUNC1, the protein ***S***hort ***P***alate ***L***ung and ***N***asal epithelial ***C***lone **1**, which is abundant in the airways^9^.

Our core objective in this work is to test these hypotheses. Our strategy of computational modeling is specifically designed to yield a deeper understanding of how DGK and phosphoinositides control ENaC activity. The proposed model consists of two modules, adapted from our previous work, that are embedded in an appropriate context and explain the regulation of ENaC function. One module addresses the phosphoinositide pathway, while the other captures the regulation of ENaC and the airway surface liquid (ASL) (Fig. 1). The merging of these modules allows, for the first time, a detailed assessment of the dynamics of ENaC regulation by phosphoinositides. Our results show that the combined model matches data from several sources remarkably well. In particular, testing our model against the observations of Almaça et al. yields good agreement. This agreement suggests that the hypothesis of ENaC regulation by DGK, accomplished though PA activation of PIP5KI, is valid and can be used to explore therapeutic interventions in clinical conditions where ENaC is thought to be relevant.

## Results

### Model expansion, parameterization and validation

The phosphoinositide pathway module was adapted from our recent work^24^ and expanded with processes that are fundamental to the regulation of the pathway. These expansions allow us to account for: (1) competition among enzymes for the same substrates^25^; (2) regulation among phosphatase and the tensin homolog (PTEN), phosphoinositide 3-kinase (PI3KI), PI(4,5)P_2_ and PI(3,4,5)P_3_; (3) the cleavage of PI(4,5)P_2_ into DAG and IP3 by phospholipase C (PLC); (4) the production of PA by the phosphorylation of DAG by DGK; (5) hydrolysis of PA back to DAG by phosphatidate phosphatases (LLP’s); (6) replenishment of the PA pool from phosphatidylcholine (PC) by phospholipase D (PLD); and (7) activation of PIP5KI, the enzyme that transforms phosphatidylinositol 4-phosphate (PI(4)P) into PI(4,5)P_2_, by PA, as previously described in the literature^26–28^.

The map for the expanded phosphoinositide pathway module is depicted in the upper box of Fig. 1. Fluxes and equations are presented in Table 1 and parameters and initial values are given in Table 2. References supporting the parameter values of the model can be found in Table S2 and in the publications underlying the original models^24,29^.

**Table 1:** Details of the integrated model.

*Rate equations*
V_→0_ = γ_→0_
V_→4_ = γ_→4_
V_→3_ = γ_→3_
V_0→3b_ = γ_0→3b_ × PI^f_0→3b_PI_ × PI3KII
V_0→3c_ = γ_0→3c_ × PI^f_0→3c_PI_ × PI(4)P^ f_0→3c_PI(4)P_ × PI3KIII
V_3→0a_ = γ_3→0a_ × PI(3)P^f_3→0a_PI(3)P_ × PI(4)P^f_3_0a_PI(4)P_ × PI(5)P^f_3_0a_PI(5)P_ × PI(3,5)P_2_^f_3_0a_PI(3,5)P2_ × PI(4,5)P_2_^(f_3_0a_PI(4,5)P2_PI_ + f_3_0a_PI(4,5)P2_PI(4)P_ + f_3_0a_PI(4,5)P2_PI(5)P_) × PI(3,4,5)P_3_^f_3_0a_PI(3,4,5)P3_ × SYNJ
V_3→0c_ = γ_3→0c_ × PI(3)P^f_3→0c_PI(3)P_ × PI(4)P^f_3_0c_PI(4)P_ × PI(5)P^f_3_0c_PI(5)P_ × PI(3,5)P_2_^f_3_0c_PI(3,5)P2_ × SAC1
V_3→0d_ = γ_3→0d_ × PI(3)P^f_3→0d_PI(3)P_ × PI(3,5)P_2_^f_3_0d_PI(3,5)P2_ × MTMR1_6_14
V_3→0e_ = γ_3→0e_ × PI(3)P^f_3→0e_PI(3)P_ × MTMR78
V_0→4_ = γ_0→4_ × PI^f_0→4_PI_ × PI4K
V_4→0a_ = γ_4→0a_ × PI(4)P^f_4→0a_PI(4)P_ × PI(3)P^f_4_0a_PI(3)P_ × PI(5)P^f_4_0a_PI(5)P_ × PI(3,5)P_2_^f_4_0a_PI(3,5)P2_ × PI(4,5)P_2_^(f_4_0a_PI(4,5)P2_PI(4)P_ + f_4_0a_PI(4,5)P2_PI(5)P_ + f_4_0a_PI(4,5)P2_PI_) PI(3,4,5)P_3_^f_4_0a_PI(3,4,5)P3_ × SYNJ
V_4→0c_ = γ_4→0c_ × PI(4)P^f_4→0c_PI(4)P_ × PI(3)P^f_4_0c_PI(3)P_ × PI(5)P^f_4_0c_PI(5)P_ × PI(3,5)P_2_^f_4_0c_PI(3,5)P2_ × SAC1
V_0→5_ = γ_0→5_ × PI^f_0→5_PI_ × PI(3)P^f_0_5_PI(3)P_ × PIKfyve
V_5→0a_ = γ_5→0a_ × PI(5)P^f_5_0a_PI(5)P_ × PI(3)P^f_5_0a_PI(3)P_ × PI(4)P^f_5_0a_PI(4)P_ × PI(3,5)P_2_^f_5_0a_PI(3,5)P2_ × PI(4,5)P_2_^(f_5_0a_PI(4,5)P2_PI(4)P_ + f_5_0a_PI(4,5)P2_PI(5)P_ + f_5_0a_PI(4,5)P2_PI_) PI(3,4,5)P_3_^f_5_0a_PI(3,4,5)P3_ × SYNJ
V_5→0c_ = γ_5→0c_ × PI(5)P^f_5_0c_PI(5)P_ × PI(3)P^f_5_0c_PI(3)P_ × PI(4)P^f_5_0c_PI(4)P_ × PI(3,5)P_2_^f_5_0c_PI(3,5)P2_ × SAC1
V_3→35_ = γ_3→35_ × PI(3)P^f_3→35_PI(3)P_ × PIKfyve
V_35→3a_ = γ_35→3a_ × PI(3,5)P_2_^f_35_3a_PI(3,5)P2_ × PI(3)P^f_35_3a_PI(3)P_ × PI(4)P^f_35_3a_PI(4)P_ × PI(5)P^f_35_3a_PI(5)P_ × PI(4,5)P_2_^f_35_3a_PI(4,5)P2_PI(4)P_ + f_35_3a_PI(4,5)P2_PI(5)P_ + f_35_3a_PI(4,5)P2_PI_) PI(3,4,5)P_3_^f_35_3a_PI(3,4,5)P3_ × SYNJ
V_35→3c_ = γ_35→3c_ × PI(3,5)P_2_^f_35_3c_PI(3,5)P2_ × PI(3)P^f_35_3c_PI(3)P_ × PI(4)P^f_35_3c_PI(4)P_ × PI(5)P^f_35_3c_PI(5)P_ × SAC1
V_35→3d_ = γ_35→3d_ × PI(3,5)P_2_^f_35→3d_PI(3,5)P2_ × SAC3
V_35→3e_ = γ_35→3e_ × PI(3,5)P_2_^f_35_3e_PI(3,5)P2_ × PI(4,5)P_2_^f_35_3e_PI(4,5)P2_ × PI(3,4,5)P_3_^f_35_3e_PI(3,4,5)P3_ × INPP5E
V_4→45_ = γ_4→45_ × PI(4)P^f_4→45_PI(4)P_ × PIP5KI × HS(PA) × PI(4,5)P_2_^f_4_45_PI(4,5)P2_
V_45→4a_ = γ_45→4a_ × PI(4,5)P_2_^f_45_4a_PI(4,5)P2_PI(4)P_ × PI(3)P^f_45_4a_PI(3)P_ × PI(4)P^f_45_4a_PI(4)P_ × PI(5)P^f_45_4a_PI(5)P_ × PI(3,5)P2^f_45_4a_PI(3,5)P2_ × PI(4,5)P_2_^(f_45_4a_PI(4,5)P2_PI(5)P_ + f_45_4a_PI(4,5)P2_PI_) × PI(3,4,5)P_3_^f_45_4a_PI(3,4,5)P3_ × SYNJ
V_45→4c_ = γ_45→4c_ × PI(4,5)P_2_^f_45_4c_PI(4,5)P2_ × PI(3,4,5)P_3_^f_45_4c_PI(3,4,5)P3_ × (ORCL1 + INPP5BJ + SKIP + SAC2)
V_45→4d_ = γ_45→4d_ × PI(4,5)P_2_^f_45_4d_PI(4,5)P2_ × PI(3,5)P_2_^f_45_4d_PI(3,5)P2_ × PI(3,4,5)P_3_^f_45_4d_PI(3,4,5)P3_ × INPP5E
V_5→45_ = γ_5→45_ × PI(5)P^f_5→45_PI(5)P_ × PIP4KII
V_45→5a_ = γ_45→5a_ × PI(4,5)P_2_^f_45_5a_PI(4,5)P2_PI(5)P_ × PI(3)P^f_45_5a_PI(3)P_ × PI(4)P^f_45_5a_PI(4)P_ × PI(5)P^f_45_5a_PI(5)P_ × PI(3,5)P_2_^f_45_5a_PI(3,5)P2_ × PI(4,5)P_2_^(f_45_5a_PI(4,5)P2_PI(4)P_ + f_45_5a_PI(4,5)P2_PI_) × PI(3,4,5)P_3_^f_45_5a_PI(3,4,5)P3_ × SYNJ
V_45→5c_ = γ_45→5c_ × PI(4,5)P_2_^f_45→5c_PI(4,5)P2_ × TMEM55
V_45→345_ = γ_45→345_ × PI(4,5)P_2_^f_45→345_PI(4,5)P2_ × PI3KI_a
V_345→45_ = γ_345→45_ × PI(3,4,5)P_3_^f_345→45_PI(3,4,5)P3_ × PI(3,4)P_2_^f_345_45_PI(3,4)P2_ × PTEN_a
V_35→5_ = γ_35→5_ × PI(3,5)P_2_^f_35→5_PI(3,5)P2_ × PI(3)P^f_35_5_PI(3)P_ × MTMR1_6_14
V_34→3_ = γ_34→3_ × PI(3,4)P_2_^f_34→3_PI(3,4)P2_ × INPP4
V_345→34a_ = γ_345→34a_ × PI(3,4,5)P_3_^f_345_34a_PI(3,4,5)P3_ × PI(3)P^f_345_34a_PI(3)P_ × PI(4)P^f_345_34a_PI(4)P_ × PI(5)P^f_345_34a_PI(5)P_ × PI(3,5)P_2_^f_345_34a_PI(3,5)P2_ × PI(4,5)P_2_^(f_345_34a_PI(4,5)P2_PI(4)P_ + f_345_34a_PI(4,5)P2_PI(5)P_ + f_345_34a_PI(4,5)P2_PI_) × SYNJ
V_345→34c_ = γ_345→34c_ × PI(3,4,5)P_3_^f_345→34c_PI(3,4,5)P3_ × PI(4,5)P_2_^f_345_34c_PI(4,5)P2_ × (ORCL1 + INPP5BJ + SKIP + SAC2)
V_345→34d_ = γ_345→34d_ × PI(3,4,5)P_3_^f_345→34d_PI(3,4,5)P2_ × PI(3,5)P_2_^f_345_34d_PI(3,5)P2_ × PI(4,5)P_2_^f_345_34d_PI(4,5)P2_ × INPP5E
V_345→34e_ = γ_345→34e_ × PI(3,4,5)P_3_^f_345→34e_PI(3,4,5)P3_ × SHIP2
V_45→_ = γ_i→_ × PI(4,5)P_2_
V_0→_ = γ_i→_ × PI
V_4→_ = γ_i→_ × PI(4)P
V_345→_ = γ_i→_ × PI(3,4,5)P_3_
V_3→_ = γ_i→_ × PI(3)P
V_35→_ = γ_i→_ × PI(3,5)P_2_
V_5→_ = γ_i→_ × PI(5)P
V_34→_ = γ_i→_ × PI(3,4)P_2_
V_0→45_ = γ_0→45_ × PI^f_0→45_PI_ × (PI4K_PIP5KI) × HS(PA) × PI(4,5)P_2_^f_0_45_PI(4,5)P2_
V_45→0_ = γ_45→0_ × PI(4,5)P_2_^f_45_0_PI(4,5)P2_PI_ × PI(3)P^f_45_0_PI(3)P_ × PI(4)P^f_45_0_PI(4)P_ × PI(5)P^f_45_0_PI(5)P_ × PI(3,5)P_2_^f_45_0_PI(3,5)P2_ × PI(4,5)P_2_^f_45_0_PI(4,5)P2_PI(4)P_ × PI(4,5)P_2_^f_45_0_PI(4,5)P2_PI(5)P_ × PI(3,4,5)P_3_^f_45_0_PI(3,4,5)P3_ × SYNJ
V_4→34a_ = γ_4→34a_ × PI(4)P^f_4→34a_PI(4)P_ × PI3KI_a
V_4→34b_ = γ_4→34b_ × PI(4)P^f_4→34b_PI(4)P_ × PI^f_4_34b_PI_ × PI3KII
V_34→4_ = γ_34→4_ × PI(3,4)P_2_^f_34→4_PI(3,4)P2_ × PI(3,4,5)P_3_^f_34_4_PI(3,4,5)P3_ × PTEN_a
PI3KI_c = PI3KI–PI3KI_a
V_PI3KIc→PI3KIa_ = γ_PI3KIc→PI3KIa_ × (PI3KI–PI3KI_a) ^ f_PI3KIc→PI3KIa_PI3KI__c × PI(3,4,5)P_3_ ^ f_PI3KIc→PI3KIa_PI(3,4,5)P3_
V_PI3KIa→PI3KIc_ = γ_PI3KIa→PI3KIc_ × PI3KI_a
PTEN_c = PTEN–PTEN_a
V_PTENc→PTENa_ = γ_PTENc→PTENa_ × (PTEN–PTEN_a) ^ f_PTENc→PTENa_PTEN_c_ × PI(4,5)P_2_ ^ f_PTENc→PTENa_PI(4,5)P2_
V_PTENa→PTENc_ = γ_PTENa→PTENc_ × PTEN_a ^ f_PTENa→PTENc_PTEN_a_
V_45→DAG_ = γ_45→DAG_ × PI(4,5)P_2_^f_45→DAG_PI(4,5)P2_ × PI(4)P^f_45→DAG_PI(4)P_ × PS^f_45→DAG_PS_ × PLC
V_PA→DAG_ = γ_PA→DAG_ × PA^f_PA→DAG_PA_ × LPP
V_DAG→PA_ = γ_DAG→PA_ × DAG^f_DAG→PA_DAG_ × DGK
V_→DAG_ = γ_→DAG_
V_DAG→_ = γ_DAG→_ × DAG^f _DAG→_DAG_
V_→PA_ = γ_→PA_
V_PA→_ = γ_PA→_ × PA^f _PA→__PA
V_PC→PA_ = γ_PC→PA_ × PI(4,5)P_2_^f_PC_PA_PI(4,5)P2_
V_IP3→_ = γ_IP3→_ × IP3^f _IP3→_IP3_
V_PI4P→DAG_ = γ_4→DAG_ × PI(4)P^f_4→DAG_PI4P_ × PI(4,5)P_2_^f_4_DAG_PI(4,5)P2_ × PS^f_4→DAG_PS_ × PLC
V_1_ = γ_1_
V_2_ = γ_2_ × ENaC
V_3_ = γ_3_ × ENaC × (SPLUNC1/ASL)
V_4_ = γ_4_
V_5_ = γ_5_
V_6_ = γ_6_ × ASL × (ENaC × ENaC_op(PI45P2))
V_7_ = γ_7_ × ASL
*Differential equations governing the expanded model*
dPI/dt = V_→0_ + V_3→0c_ + V_3→0d_ + V_3→0e_ + V_4→0a_ + V_4→0c_ + V_5→0a_ + V_5→0c_ + V_45→0_ − V_0→3a_ − V_0→3b_ − V_0→3c_ − V_0→4_ − V_0→5_ − V_0→45_ − V_0→_
dPI3P/dt = V_→3_ + V_0→3a_ + V_0→3b_ + V_0→3c_ + V_35→3a_ + V_35→3c_ + V_35→3d_ + V_35→3e_ + V_34→3_ − V_3→0c_ − V_3→0d_ − V_3→0e_ − V_3→35_ − V_3→_
dPI4P/dt = V_→4_ + V_0→4_ + V_45→4a_ + V_45→4c_ + V_45→4d_ + V_34→4_ − V_4→0a_ − V_4→0c_ − V_4→45_ − V_4→34a_ − V_4→34b_ − V_PI(4)P→DAG_ − V_4→_
dPI5P/dt = V_0→5_ + V_35→5_ + V_45→5a_ + V_45→5c_ − V_5→0a_ − V_5→0c_ − V_5→45_ − V_5→_
dPI35P2/dt = V_3→35_ − V_35→3a_ − V_35→3c_ − V_35→3d_ − V_35→3e_ − V_35→5_ − V_35→_
dPI45P2/dt = V_4→45_ + V_5→45_ + V_345→45_ + V_0→45_ − V_45→4a_ − V_45→4c_ − V_45→4d_ − V_45→5a_ − V_45→5c_ − V_45→345_ − V_45→0_ − V_45→_ − V_45→DAG_
dPI34P2/dt = V_345→34a_ + V_345→34c_ + V_345→34d_ + V_345→34e_ + V_4→34a_ + V_4→34b_ − V_34→4_ − V_34→3_ − V_34→_
dPI345P3/dt = V_45→345_ − V_345→45_ − V_345→34a_ − V_345→34c_ − V_345→34d_ − V_345→34e_ − V_345→_
dDAG/dt = V_→DAG_ + V_PA→DAG_ + V_4→DAG_ + V_45→DAG_ − V_DAG→PA_ − V_DAG→_
dIP3/dt = V_45→DAG_ − V_IP3→_
dPA/dt = V_→PA_ + V_DAG→PA_ + V_PC→PA_ − V_PA→DAG_ − V_PA→_
dpi_3KI_a/dt = V_PI3KIc→PI3KIa_ − V_PI3KIa→PI3KIc_
dPTEN_a/dt = V_PTENc→PTENa_ − V_PTENa→PTENc_
dENaC/dt = V_1_ − V_2_ − V_3_
dASL/dt = V_4_ + V_5_ − V_6_ − V_7_

**Table 2:** Model parameters and initial values.

Initial values					
Units are molecules/μm^2^ except for ASL which is μm					
PI	290,115.435	PI3P	94.624	PI4P	11,095.701
PI5P	97.177	PI35P2	21.342	PI45P2	9,851.421
PI34P2	40.358	PI345P3	1.128	pi_3KI_a	5.827
PTEN_a	14.551	DAG	5,787.001	IP3	499.14
PA	8,025.198	ENaC	35	ASL	7

Enzyme concentrations					
Units are mg except for PS which is molecules/μm^2^					
PI3KI	2.046E−13*	SYNJ	9.227E−15*	TMEM55	9.452E−16*
PI3KII	2.253E−14*	SAC1	1.112E−14*	ORCL1	5.189E−16*
PI3KIII	8.429E−16*	SAC2	2.132E−16*	SKIP	4.240E−16*
PI4K	7.285E−14*	SAC3	8.602E−16*	SHIP2	2.301E−16*
PIP5KI	3.596E−14*	MTMR1_6_14	7.313E−15*	PLC	7.339E−16^#^
PI4K_PIP5KI	9.492E−14*	MTMR78	2.266E−15*	DGK	9.711E−15^#^
PIP4KII	6.588E−14*	INPP4	8.910E−15*	LPP	5.407E−15^#^
PIKfyve	1.889E−14*	INPP5BJ	1.606E−16*	PS	150,000^#^
PTEN	3.915E−15*	INPP5E	1.165E−16*		

Rate constants (γ) and kinetic orders (f)					
Units for γ are molecules^1−g^ μm^2g^/min or μm^2^/min/mg where g is the kinetic order of the corresponding variable. f_i→j_ is dimensionless.					
γ_→0_	1.500E+04*	f_35→3a_PI5P_	− 4.400E−04*	f_345→34a_PI45P2_PI5P_	− 2.383E−01*
γ_→4_	1.500E+02*	f_35→3a_PI35P2_	9.998E−01*	f_345→34a_PI345P3_	9.983E−01*
γ_→3_	1.000E+01*	f_35→3a_PI45P2_PI4P_	− 4.385E−02*	f_345→34a_PI45P2_PI_	− 1.096E−02*
γ_0→3b_	8.810E+14*	f_35→3a_PI45P2_PI5P_	− 2.383E−01*	γ_345→34c_	2.790E+11*
f_0→3b_PI_	1.761E−01*	f_35→3a_PI345P3_	− 1.670E−03*	f_345→34c_PI345P3_	9.976E−01*
f_0→3b_PI4P_	− 7.030E−02*	f_35→3a_PI45P2_PI_	− 1.096E−02*	f_345→34c_PI45P2_	− 6.214E−02*
γ_0→3c_	5.460E+14*	γ_35→3c_	5.100E+12*	γ_345→34d_	2.790E+11*
f_0→3c_PI_	1.138E−01*	f_35→3c_PI35P2_	9.997E−01*	f_345→34d_PI345P3_	9.976E−01*
γ_3→0a_	4.560E+13*	f_35→3c_PI3P_	− 6.200E−04*	f_345→34d_PI35P2_	− 3.100E−04*
f_3→0a_PI3P_	9.996E−01*	f_35→3c_PI4P_	− 6.219E−02*	f_345→34d_PI45P2_	− 6.212E−02*
f_3→0a_PI4P_	− 4.385E− 02*	f_35→3c_PI5P_	− 6.200E− 04*	γ_345→34e_	1.680E+11*
f_3→0a_PI5P_	− 4.385E−04*	γ_35→3d_	3.060E+12*	f_345→34e_PI345P3_	9.982E−01*
f_3→0a_PI35P2_	− 2.193E−04*	f_35→3d_PI35P2_	9.999E−01*	γ_i→_	4.500E−02*
f_3→0a_PI45P2_PI4P_	− 4.385E−02*	γ_35→3e_	5.140E+12*	γ_0→45_	2.670E+14
f_3→0a_PI45P2_PI5P_	− 2.383E−01*	f_35→3e_PI35P2_	9.997E−01*	f_0→45_PI_	2.865E−01*
f_3→0a_PI345P3_	− 1.668E−03*	f_35→3e_PI45P2_	− 6.212E−02*	f_0→45_PA_	2.000E−01^#^
f_3→0a_PI45P2_PI_	− 1.096E−02*	f_35→3e_PI345P3_	− 2.360E−03*	f_0→45_PI45P2_	− 5.000E−02^#^
γ_3→0c_	5.100E+12*	γ_4→45_	8.490E+15	γ_4→34b_	5.640E+14*
f_3→0c_PI3P_	9.994E−01*	f_4→45_PI4P_	4.596E−02	f_4→34b_PI4P_	9.297E−01*
f_3→0c_PI4P_	− 6.219E−02*	f_4→45_PA_	2.000E−01^#^	f_4→34b_PI_	− 8.239E−01*
f_3→0c_PI5P_	− 6.219E−04*	f_4→45_PI45P2_	− 5.000E−02^#^	γ_34→4_	5.040E+11*
f_3→0c_PI35P2_	− 3.110E−04*	γ_45→4a_	4.560E+13*	f_34→4_PI34P2_	9.999E−01*
γ_3→0d_	3.060E+12*	f_45→4a_PI3P_	− 4.400E−04*	f_34→4_PI345P3_	− 4.443E−02*
f_3→0d_PI3P_	9.993E−01*	f_45→4a_PI4P_	− 4.385E−02*	γ_45→0_	1.140E+12*
f_3→0d_PI35P2_	− 3.318E−04*	f_45→4a_PI5P_	− 4.400E−04*	f_45→0_PI3P_	9.996E−01*
γ_3→0e_	3.070E+12*	f_45→4a_PI35P2_	− 2.200E−04*	f_45→0_PI4P_	− 4.385E−02*
f_3→0e_PI3P_	9.993E−01*	f_45→4a_PI45P2_PI4P_	9.561E−01*	f_45→0_PI5P_	− 4.400E−04*
γ_0→4_	5.100E+14	f_45→4a_PI45P2_PI5P_	− 2.383E−01*	f_45→0_PI35P2_	− 2.200E−04*
f_0→4_PI_	2.865E−01	f_45→4a_PI345P3_	− 1.670E−03*	f_45→0_PI45P2_PI4P_	− 4.385E−02*
γ_4→0a_	4.560E+13*	f_45→4a_PI45P2_PI_	− 1.096E−02*	f_45→0_PI45P2_PI5P_	− 2.383E−01*
f_4→0a_PI3P_	− 4.385E−04*	γ_45→4c_	5.130E+12*	f_45→0_PI345P3_	− 1.670E−03*
f_4→0a_PI4P_	9.561E−01*	f_45→4c_PI45P2_	9.379E−01*	f_45→0_PI45P2_PI_	− 1.096E−02*
f_4→0a_PI5P_	− 4.385E−04*	f_45→4c_PI345P3_	− 2.360E−03*	γ_PI3KIc→PI3KIa_	3.350E−06^#^
f_4→0a_PI35P2_	− 2.193E−04*	γ_45→4d_	5.140E+12*	f_PI3KIc→PI3KIa_pi_3KI_c_	1.000E+00^#^
f_4→0a_PI45P2_PI4P_	− 4.385E−02*	f_45→4d_PI45P2_	9.379E−01*	f_PI3KIc→PI3KIa_PI345P3_	7.000E−01^#^
f_4→0a_PI45P2_PI5P_	− 2.383E−01*	f_45→4d_PI35P2_	− 3.100E−04*	γ_PI3KIa→PI3KIc_	6.220E−04^#^
f_4→0a_PI345P3_	− 1.668E−03*	f_45→4d_PI345P3_	− 2.360E−03*	f_PI3KIa→PI3KIc_pi_3KI_a_	1.000E+00^#^
f_4→0a_PI45P2_PI_	− 1.096E−02*	γ_5→45_	2.950E+13	γ_PTENc→PTENa_	1.250E−07^#^
γ_4→0c_	5.100E+12*	f_5→45_PI5P_	8.784E−01	f_PTENc→PTENa_PTEN_c_	1.000E+00^#^
f_4→0c_PI4P_	9.378E−01*	γ_45→5a_	3.240E+12*	f_PTENc→PTENa_PI45P2_	1.000E+00^#^
f_4→0c_PI3P_	− 6.219E−04*	f_45→5a_PI3P_	− 4.400E−04*	γ_PTENa→PTENc_	3.000E−03^#^
f_4→0c_PI5P_	− 6.219E−04*	f_45→5a_PI4P_	− 4.385E−02*	f_PTENa→PTENc_PTEN_a_	1.000E+00^#^
f_4→0c_PI35P2_	− 3.110E−04*	f_45→5a_PI5P_	− 4.400E−04*	f_PTENa→PTENc_PI345P3_	1.000E+00^#^
γ_0→5_	4.700E+11*	f_45→5a_PI35P2_	− 2.200E−04*	γ_45→DAG_	1.880E+19^#^
f_0→5_PI_	5.465E−01*	f_45→5a_PI45P2_PI4P_	− 4.385E−02*	f_45→DAG_PI45P2_	9.770E−01^#^
f_0→5_PI3P_	− 3.779E−04*	f_45→5a_PI45P2_PI5P_	7.617E−01*	f_45→DAG_PI4P_	− 1.260E−02^#^
γ_5→0a_	4.560E+13*	f_45→5a_PI345P3_	− 1.670E−03*	f_45→DAG_PS_	− 9.642E−01^#^
f_5→0a_PI3P_	− 4.385E−04*	f_45→5a_PI45P2_PI_	− 1.096E−02*	γ_PA→DAG_	5.540E+11^#^
f_5→0a_PI4P_	− 4.385E−02*	γ_45→5c_	4.130E+12*	f_PA→DAG_PA_	9.653E−01^#^
f_5→0a_PI5P_	9.996E−01*	f_45→5c_PI45P2_	6.487E−01*	γ_DAG→PA_	1.650E+13^#^
f_5→0a_PI35P2_	− 2.200E−04*	γ_45→345_	1.890E+14	f_DAG→PA_DAG_	9.476E−01^#^
f_5→0a_PI45P2_PI4P_	− 4.385E−02*	f_45→345_PI45P2_	3.063E−01	γ_→DAG_	1.000E−05^#^
f_5→0a_PI45P2_PI5P_	− 2.383E−01*	γ_345→45_	2.900E+15*	γ_DAG→_	1.000E−01^#^
f_5→0a_PI345P3_	− 1.670E−03*	f_345→45_PI345P3_	9.556E−01*	f_DAG→_DAG_	1.000E+00^#^
f_5→0a_PI45P2_PI_	− 1.096E−02*	f_345→45_PI34P2_	− 1.100E−04*	γ_→PA_	4.000E+00^#^
γ _5→0c_	5.100E+12*	γ_35→5_	6.060E+14*	γ_PA→_	1.000E−01^#^
f_5→0c_PI5P_	9.994E−01*	f_35→5_PI35P2_	9.997E−01*	f _PA→_PA_	1.000E+00^#^
f_5→0c_PI3P_	− 6.200E−04*	f_35→5_PI3P_	− 6.600E−04*	γ_PC→PA_	1.436E+01^#^
f_5→0c_PI4P_	− 6.219E−02*	γ_34→3_	1.330E+12	f_PC→PA_PI45P2_	3.000E−01^#^
f_5→0c_PI35P2_	− 3.100E−04*	f_34→3_PI34P2_	9.982E−01	γ_IP3→_	2.000E+00^#^
γ_3→35_	1.620E+16*	γ_345→34a_	2.480E+12*	f _IP3→_IP3_	1.000E+00^#^
f_3→35_PI3P_	9.996E−01*	f_345→34a_PI3P_	− 4.400E−04*	γ_4→DAG_	2.550E+18^#^
f_3→35_PI_	− 4.535E−01*	f_345→34a_PI4P_	− 4.385E−02*	f_4→DAG_PI4P_	9.874E−01^#^
γ_35→3a_	4.560E+13*	f_345→34a_PI5P_	− 4.400E−04*	f_4→DAG_PI45P2_	− 2.296E−02^#^
f_35→3a_PI3P_	− 4.400E−04*	f_345→34a_PI35P2_	− 2.200E−04*	f_4→DAG_PS_	− 9.642E−01^#^
f_35→3a_PI4P_	− 4.385E−02*	f_345→34a_PI45P2_PI4P_	− 4.385E−02*		

Parameters for the ENaC/ASL model					
Units for γ are molecules^1−g^ μm^2g^/min or μm^2^/min/mg where g is the kinetic order of the corresponding variable. SPLUNC1 is in molecules/μm^2^					
γ_1_	0.0173	γ_2_	0.000217	γ_3_	0.000000247
γ_4_	0.0617	γ_5_	0.0177	γ_6_	0.00039
γ_7_	0.00803	SPLUNC1	7,890		

Values with * refer to parameters that were altered and values with ^#^ refer to new parameters. All other parameter values were taken, unchanged, from the previous models^24,29^.

**Figure 3:**
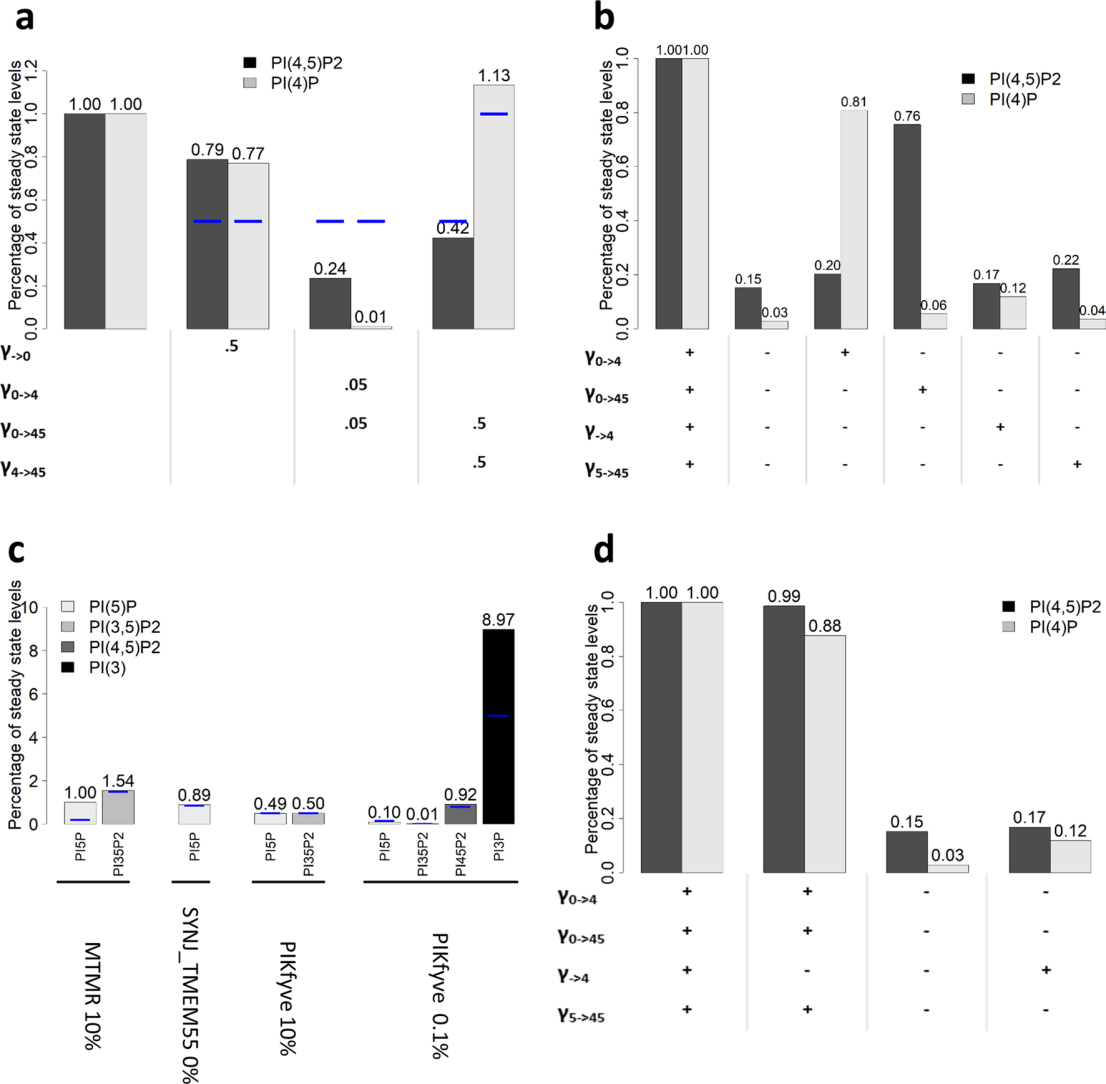
Perturbations to the phosphoinositide pathway. Blue lines represent experimental observations and bars represent model predictions. (**a**) Perturbation of PI levels and PI4K and PI5KI activities and resulting effects on PI(4,5)P_2_ and PI(4)P. γ_→0_ is reduced to 50% to trigger a decrease of 50% in PI. (**b**) Perturbation of input fluxes into the pools of PI(4)P and PI(4,5)P_2_. After stopping all inputs into PI(4)P and PI(4,5)P_2_, the inputs are re-activated, one at a time, to test if they are sufficient to restore PI(4,5)P_2_ levels. Enzyme knockouts were simulated by setting the rate constant of the corresponding fluxes to zero, except for γ_0→4_, which was decreased to 20% of its original value, in order to avoid numerical errors in the simulation due to very small levels of PI(4)P. (**c**) Perturbations to MTMR, SYNJ_TMEM55 and PIKfyve that were used to fit the model to the behavior of phosphoinositides with small pools: PI5P, phosphatidylinositol 3,5-bisphosphate (PI(3,5)P_2_) and phosphatidylinositol 3-phosphate (PI(3)P). (**d**) Consequences of Golgi PI(4)P input (γ_→4_) for the levels of PI(4)P and PI(4,5)P_2_ pools. Golgi PI(4)P has a significant impact on the PI(4)P pool but barely affects the PI(4,5)P_2_ pool. γ_a→b_ denotes the rate constant of the flux that transforms a into b. γ_→0_ denotes an influx of material into the system, in this case into the PI pool. γ_4→_ denotes an efflux of material out of the system, in this case from the PI(4)P pool. Some examples: γ_→0_ denotes the rate constant of the influx of material to the PI pool from the exterior of the system, γ_0→4_ denotes the rate constant of the flux that represents the transformation of PI into PI(4)P, γ_5→45_ denotes the rate constant of the flux that represents the transformation of PI(5)P into PI(4,5)P_2_.

The earlier phosphoinositide pathway model was successfully tested against a long list of phenomena reported in the literature^24^. Corresponding results for the extended phosphoinositide model are summarized in Fig. 3. As is to be expected, the new modules and extensions slightly affect the model fits in comparison to the previous sub-model, but the combined phosphoinositide-ENaC model yields fits to the data that are similar. In addition, the combined model generates genuinely new results, which are summarized in Table S1 and detailed in the following, along with reports from the literature.

Figure 3a, c show model results as bar plots, while the blue lines represent data. When phosphatidylinositol (PI) is depleted, the decreases in PI(4)P and PI(4,5)P_2_ are now slightly closer to 50%, whereas the results of perturbations in PI4K and PI4P5KI are somewhat inferior (Fig. 3a). Perturbations that affect the lipids to a smaller degree produce similar results (Fig. 3c).

Figure 3b indicates that we can still create conditions where PI(4,5)P_2_ levels are maintained with low levels of PI(4)P, but the PI(4,5)P_2_ pool is much more dependent on the V_0→45_ flux. In the new model, the contribution of phosphatidylinositol 5-phosphate (PI(5)P) to the PI(4,5)P_2_ pool is small. Figure 3c, d show that PI(4)P is also more dependent on the V_0→4_ flux.

Several other datasets were retrieved from the literature and used to fit and validate the extended phosphoinositide pathway module. These results are described in the Supplements (Sections 1 and 3), where also further details about the phosphoinositide model expansion can be found.

The second module captures the dynamics of ENaC and ASL regulation by SPLUNC1 and PI(4,5)P_2_ (Fig. 1, lower box); it was fully described in our previous work^29^. This module simulates the number of ENaCs, which are regulated by SPLUNC1 and ASL thickness. ASL thickness, in turn, is regulated by CFTR and ENaC activities. The latter is the product of the number of ENaCs and their collective open probability (P_o_), which is controlled by PI(4,5)P_2_ levels. The ENaC—ASL model simulates both healthy and CF lungs through the change of CFTR activity.

The phosphoinositide pathway and ENaC—ASL modules were constructed to simulate the dynamics of regulation in a 1 µm thick patch of cell membrane in human bronchial epithelial cells. Fluxes and equations are presented in Table 1, and parameters and initial values are given in Table 2.

The key connection between the two modules is PI(4,5)P_2_. The phosphoinositide pathway module determines the PI(4,5)P_2_ levels, while the ENaC—ASL module translates these levels into ENaC activity. Therefore, the combined model permits novel explorations of the interactions between phosphoinositides and the dynamics of ENaC and ASL (Fig. 1).

### The combined model with PIP5KI activation by PA replicates the effects of a DGK knockdown

One hypothesis to be tested with the proposed model is that the DGK knockdown effect on ENaC activity is mediated by PI(4,5)P_2_ through PA control of PIP5KI. The work of Antonescu et al.^30^ demonstrated that inhibition of DGK causes a decrease of about 50% in the level of PA. If our hypothesis regarding the role of DGK is valid, the reduction in PA should decrease PI(4,5)P_2_ production by PIP5KI. Indeed, the model exhibits a decrease of 52% in PA when DGK is inhibited 75%. The same perturbation reduces PI(4,5)P_2_ by 28%.

Almaça et al.^21^ performed siRNA screens to identify modulators of ENaC activity and observed that DGK inhibition reduces ENaC activity to WT basal levels in CF F508del cells, but has no significant effect in WT cells. In CF, when PLC is inhibited, DGK inhibition has no effect on ENaC. When PLC is activated, ENaC function drops to WT basal levels, and inhibiting DGK causes an additional small decrease in ENaC function.

**Figure 4:**
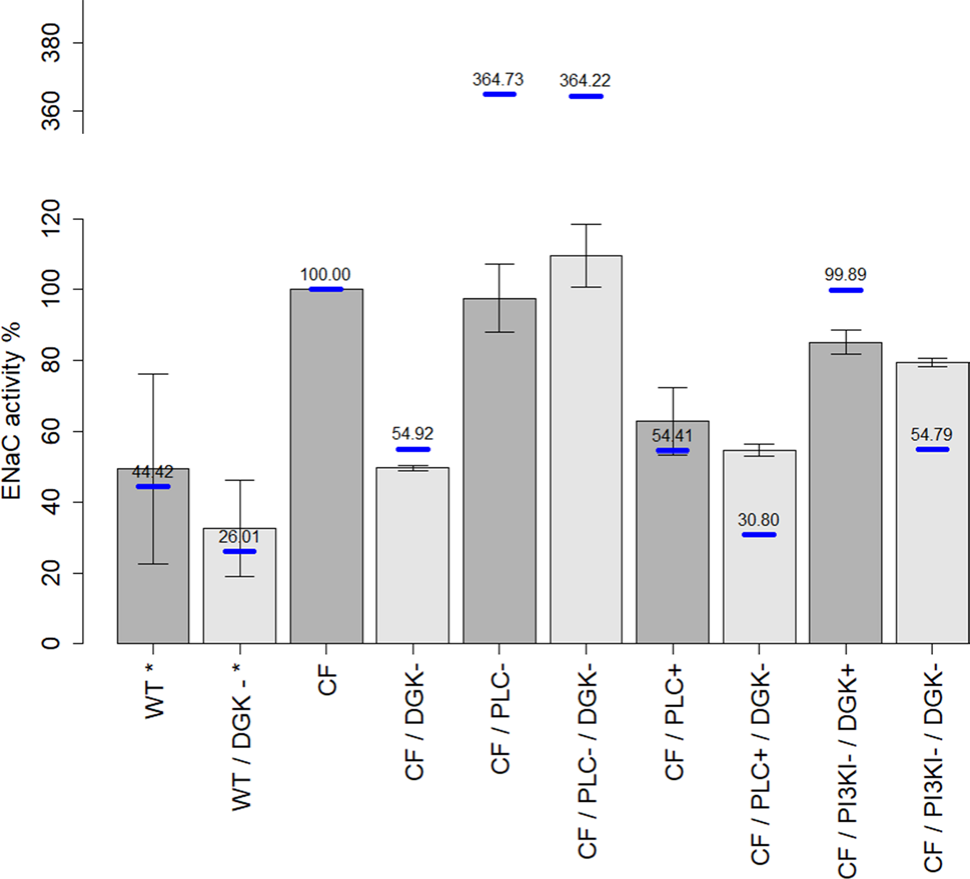
Model results demonstrating consequences of DGK inhibition on ENaC activity in healthy subjects (WT) and patients with CF, CF with PLC inhibition, CF with PLC activation, and CF with PI3KI inhibition of ENaC activity. Bars and confidence intervals represent data, as reported by Almaça et al.^21^. The dark grey bars correspond to basal levels of DGK activity, the light grey bars correspond to 25% inhibition of DGK (DGK−). Blue lines represent model results. (1^st^ to 4^th^ bars) DGK inhibition in CF (CF/DGK−) reduces ENaC’s activity to a similar level as the channel activity in WT, as described by Almaça et al.^21^. (5^th^ and 6^th^ bars) With PLC inhibition, a decrease in PI(4,5)P_2_ caused by DGK inhibition does not affect ENaC’s action, as proposed by Almaça et al.^21^. The discrepancy between the data and the model results are probably due to saturation of the florescence reporter used. (7^th^ and 8^th^ bars) When PLC is activated, ENaC activity is reduced. DGK inhibition further reduces the channel activity. The data provided by Almaça et al. suggest a decrease after DGK inhibition, which however is not significant. *Data for WT and WT/DGK− were collected using transepithelial voltage measurements; otherwise, a voltage dependent FMP assay was used (*cf.* Almaça et al.^21^ for further details).

A comparison between model results and data is illustrated in Fig. 4. Given the good qualitative (and semi-quantitative) agreement between the data and the results from our model analyses, we can cautiously conclude that our hypothesis regarding the mechanisms of ENaC regulation provides a good explanation for the activity of ENaC in WT and CF. Our combined model tests the effect of the activation of PIP5KI by PA, but, in this implementation, cannot test the PI recycling hypothesis, as it focuses on a plasma membrane patch. Further details about a pertinent model simulation can be found in the Supplements (Section 2.1).

Some discrepancies persist when PLC is perturbed. In the case of PLC inhibition (CF\PLC-), Almaça’s data indicate the same magnitude of ENaC activity as in the unperturbed CF state (CF), while the model predicts a much higher value. This discrepancy is presumably due to the fact that Almaça et al. used a fluorescence essay where the marker was saturated for values around 100% of ENaC activity. In the case of PLC activation (CF\PLC+), the model replicates the case without DGK perturbation but when the kinase is inhibited, a slightly more pronounced reduction in ENaC activity is predicted by the model.

### The combined model with phosphoinositide recycling, but without PIP5KI activation by PA, does not replicate the effects of a DGK knockdown

Phosphoinositides are synthesized in the ER from PA and transported to the plasma membrane where they are phosphorylated, cleaved by PLC into IP3 and DAG and transformed back to PA, which is transported back to the ER, thereby closing a functional cycle. Almaça et al.^21^ hypothesized that inhibiting DGK would bring the recycling of phosphoinositides to a halt, which in turn would decrease PI(4,5)P_2_ and consequently reduce ENaC action.

To test the hypothesis of Almaça and colleagues, we temporarily altered our model by removing the regulation of PIP5KI by PA and allowing the efflux from the PA pool, V_PA→_, to supply the PI pool with material, which simulates the transformation of PA into PI in the ER. When DGK is inhibited in this configuration, it no longer influences ENaC activity (Figure S7a). This result happens because the efflux of PA is only 5% of the influx of PI. Furthermore, the influx of PI, which is independent of the plasma membrane PA efflux (V_→0_) that represents PA created de novo in the ER, is enough to sustain the PI pool when DGK is inhibited.

ENaC can be made sensitive to DGK if the much larger PI influx is made exactly proportional to the PA efflux, which however is unrealistic (details in the Supplements (Section 2.2) and Figure S7c).

### Suratekar’s phosphoinositide cycle model replicates DGK knockdown effects only if PIP5KI is activated by PA

Suratekar et al.^31^ recently modeled the phosphoinositide cycle in the plasma membrane and in the ER. Coupling our ENaC—ASL module with their model allowed us to test Almaça’s hypothesis in a different, almost independent manner.

**Figure 5:**
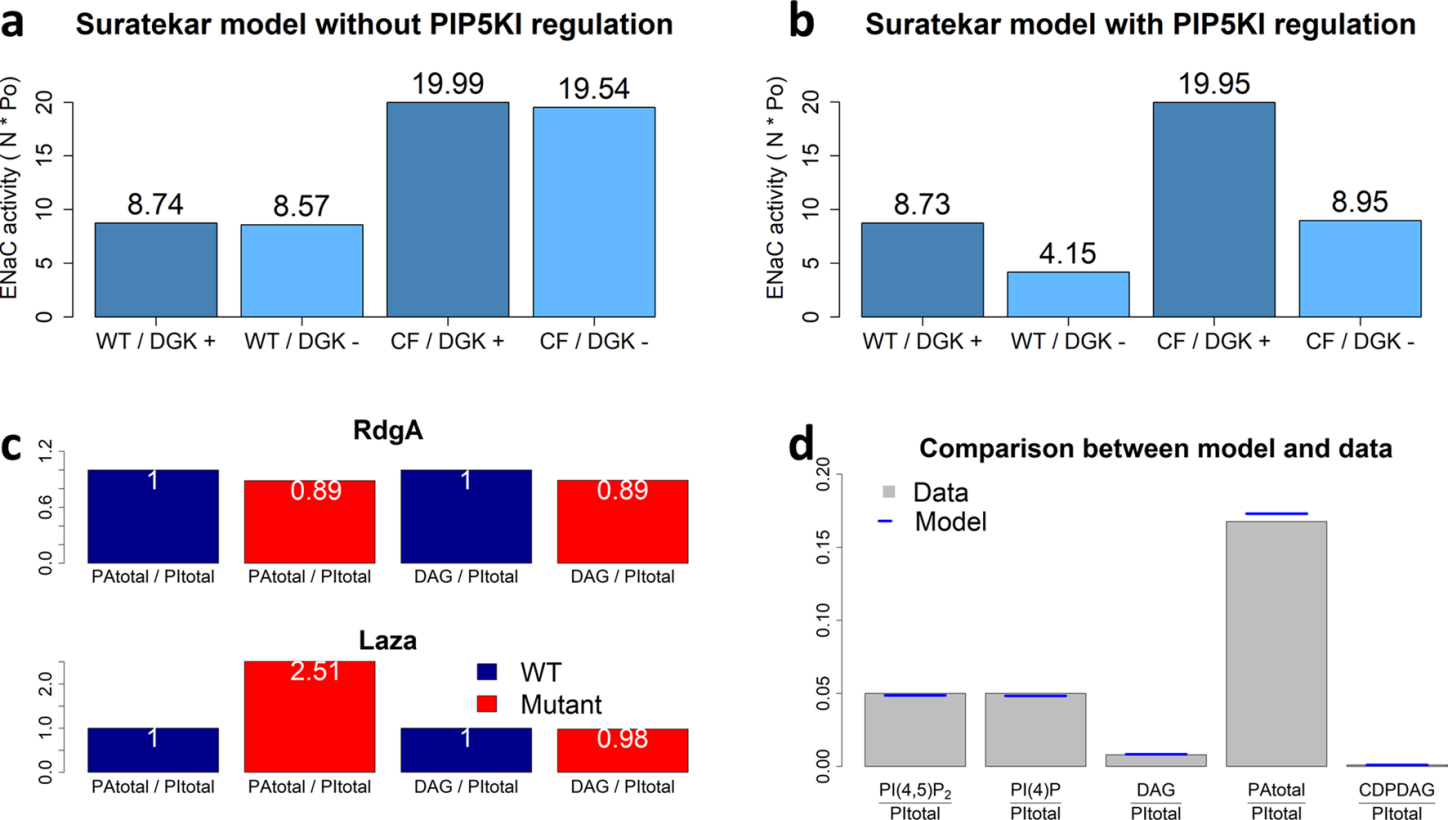
Suratekar’s model of phosphoinositide recycling by itself does not replicate Almaça’s observations of ENaC moderation when DGK is inhibited. The results presented here were obtained with a model combining the Suratekar model with our ENaC—ASL model. Panels (**a**) and (**b**) represent ENaC activity under healthy (WT) and CF conditions. (**a**) Data from the Suratekar model fail to replicate experimental observations. (**b**) If Suratekar’s model of phosphoinositide recycling is modified to include PA regulation of PIP5KI and an efflux from the PI(4)P pool, the resulting model *does* replicate Suratekar’s data and Almaça’s observations of DGK control of ENaC. DGK inhibition causes a moderation of ENaC’s action. In CF, this moderation brings the ENaC activity close to the WT ENaC activity. (**c**) Plots represent simulated lipid ratios in *Drosophila melanogaster* photoreceptor cell mutants relative to WT lipid ratios presented in panel** d**.

We used the version of the Suratekar model that the authors showed to be consistent with all data available. This version uses Michaelis–Menten kinetics to represent an open cycle with influx of PA into the ER and efflux out of DAG into the plasma membrane; it does not include regulation of PIP5KI by PA. This representation turned out to be inconsistent with Almaça’s observations of ENaC moderation under DGK inhibition (Fig. 5a). Suratekar’s model contains an influx of PA into the ER that is independent of plasma membrane PA. When we inhibit DGK, which severely reduces PA in the plasma membrane, this influx suffices to maintain the necessary phosphoinositide levels.

When DGK is inhibited, plasma membrane PA decreases by 90%. This effect suggests that the inclusion of regulation of PIP5KI by PA in the plasma membrane might enable Suratekar’s model to replicate Almaça’s observations. We implemented this regulation in two ways: first, by changing the *V*_*max*_ of PIP5KI from a constant into a Hill function of plasma membrane PA and, second, by making the *K*_*M*_ of PIP5KI inversely proportional to plasma membrane PA. In both cases, the inhibition of DGK reduces PA in the membrane, and this reduction is transmitted to PIP5KI, resulting in a decreased rate of PI(4,5)P_2_ production. But, as PIP5KI loses efficiency, PI(4)P accumulates rapidly. This accumulation continues until the amount of PI(4)P compensates for the reduced efficiency of PIP5KI, thereby establishing a new steady state with a very elevated PI(4)P level that restores the levels of PI(4,5)P_2_.

Assigning a small rate constant (0.08) to the efflux from the pool of PI(4)P creates an “escape valve” that prevents PI(4)P from unduly accumulating when DGK is inhibited. This setting very slightly alters the steady state, but is easily balanced by increasing the endoplasmic reticulum PA source by 1.1%, which compensates for the new loss of material from the system. These settings lead to a model that is consistent with Suratekar’s data and replicates Almaça’s observations (Fig. 5b). More details on this matter can be found in Section 2.4 of the supplements.

Figures 5c, d permit an easy comparison with the analogous figures in the paper of Suratekar et al.^31^ (namely Figures S6, S8 and S9 in the supplements and Table 2 in the main text of their paper). Thus, one can readily confirm that the altered model satisfies the conditions that were required to validate the original model.

Taking all results together gives us confidence that our hypothesis of DGK moderating ENaC through PA control of PIP5KI convincingly explains the available data.

## Discussion and Conclusions

The proposed work combines two models: one representing the dynamics of phosphoinositides and the other accounting for the regulation of ENaC and ASL. This combination allowed a detailed study of the intricate interactions between DGK and ENaC and yielded good consistency with available data.

Several results in the literature support our hypothesis that PIP5KI regulation by PA explains the moderation of ENaC activity induced by DGK knockdown, as Almaça et al.^21^ had observed. From the work of Moritz^26^, Jarquin-Pardo^28^ and Jenkins^27^ we know that reducing PA downregulates PIP5KI in vitro. The inhibition of DGK affecting PIP5KI is supported by in vitro data presented in our previous paper^24^ and by Luo, Prescot and Topham^32^ in Zebrafish, although this finding was contested by Jones, Sanjuan and Mérida^33^.

To the best of our knowledge, only Sandefur and colleagues^34^ made an attempt to study EnaC regulation by phosphoinositides. However, these authors did not consider SPLUNC1 and practically ignored phosphoinositides, only referring to them as mediators of P2Y_2_ purinoreceptor signaling, which is activated by extracellular adenosine triphosphate (ATP). This simplification has the crucial disadvantage that it becomes difficult to study the regulation of PIP5KI by DGK, which could be a promising drug target.

As mentioned before, several studies showed that PI(4,5)P_2_ and PI(3,4,5)P_3_ both affect ENaC, but we only focused on PI(4,5)P_2._ The rationale is that we are interested in human pulmonary epithelial cells, which are polarized, with a cell membrane that has different characteristics in different locations. So far, the data of highest quality point to a uniform distribution of PI(4,5)P2 throughout the plasma membrane and absence of PI(3,4,5)P_3_ in the apical part of polarized cells^35^, where CFTR and ENaC are located.

The past years have witnessed numerous discoveries regarding lipid transfer proteins (LPTs) (e.g.,^36^) and it is conceivable that a so-far unknown cellular mechanism could create the high sensitivity of PA production in the endoplasmic reticulum to the levels of PA in the plasma membrane, which would validate the hypothesis of Almaça et al. However, extensive, targeted research over several decades has not identified such a mechanism. It therefore appears that our hypothesis regarding ENaC regulation by DGK has a higher likelihood of being correct than the earlier hypothesis of Almaça and colleagues. According to this new hypothesis, which is supported by our computational analyses, the regulation of ENaC is primarily exerted through the control of PI(4,5)P_2_ production by PIP5KI, which in turn is controlled by PA, the product of the DGK reaction.

ENaC is composed of three subunits, which are usually called α, β and γ^37^. Channels with alternate stoichiometries have been reported to have very low activity^38^, so that some ENaC channels could be considered constitutively closed. Also, not all ENaC channels are necessarily equal, due to variability introduced by alternative splicing, alternative folding, glycosylation and ubiquitination. Thus, one might expect a range of ENaCs where, at one end, some channels are open even if ENaC controllers are signaling a closed configuration, and where the opposite is true at the other end. If there are indeed constitutively open and closed ENaC channels, the open probability function would have a higher minimum than the reported value, which is about 0.02^39^. By the same token, as a result of constitutively closed channels, the maximum should be lower than reported. To explore this situation, we tested an alternative ENaC P_o_ function with a minimum of 0.12, a basal ENaC P_o_ of 0.22, and maximum of 0.72. With this P_o_ function, the model exhibit results that are similarly good as previous findings but yields much improved results with respect to perturbations in PLC activation. This alteration would likely make the activity of ENaC less sensitive to DGK inhibition and yield model results that are more similar to observations by Almaça and colleagues. Unfortunately, there are no data supporting this strategy.

**Figure 6:**
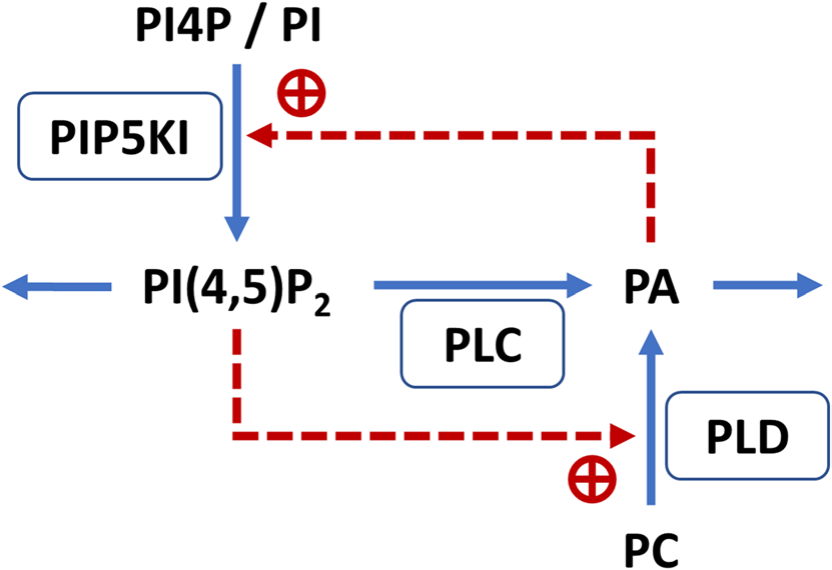
Hypothetical positive feedback regulation of PI(4,5)P_2_ and PA. In the plasma membrane, PA may be created from PC by PLD and by cleavage of PI(4,5)P_2_ by PLC. When PLC displays low activity, PA is mainly produced by PLD. When PLC activity increases, more PA is produced via degradation of PI(4,5)P_2_, which lowers the pool of PI(4,5)P_2_, secondarily reduces the activation of PLD, and ultimately leads to less PA production from PC. This dual activation mechanism could alter the ratio between PA coming from PLC and from PLD and contribute to the stability of PA and PI(4,5)P_2_ levels; however, confirmation of this mechanism will require further experimental investigation.

It would be beneficial to study in greater depth the positive feedback loop between PI(4,5)P_2_, PLC, PA and PLD (Fig. 6), which was previously identified by van der Bout and Divecha^40^. Not only would it be interesting to see the regulatory capabilities of this functional arrangement, but one could also study intriguing observations like the one made by Antonescu et al.^30^, where the knock-down of an PLD isoform led to increased PA levels. At current count, there are ten DGK isoforms^41,42^, thirteen PLC isoforms^43^, two PLD isoforms^44^ and nine protein kinase C (PKC) isoforms. We did not account for this detail, but the diversity could be important for shedding light on some of the so far unexplained observations in the field. At the same time, the subcellular locations of PLD are still a matter of controversy and PLD may not be abundant in the plasma membrane^45^. Also, PLC activation is currently represented in a rather simplified manner, because the model does not include calcium (Ca^2+^) release. Thus, discrepancies concerning PLC are likely caused by a missing or so-far unknown mechanism that accounts for Ca^2+^ release and its feedback effect on PLC. Also, the time scale of our model is in minutes and much of the pertinent phenomena are better visualized in seconds, as can be seen in supplement Figure S4 and in other studies^46,47^. This aspect could be the topic of a possible refinement to be taken into account in the future. PI(4,5)P2 recovery after PLC activation is a complicated subject that has caused other researchers similar difficulties^48,49^, and the present model is also not able to explain it convincingly. However, when the PLC activation in our model is artificially sped up in a manner similar to what was proposed in the literature^48,49^, discrepancies with respect to PLC activation disappear. Further details are presented in the Supplement subsection on PI(4,5)P_2_ dynamics in PLC perturbations (1.3). Importantly, with or without this speed-up, the discrepancy has no real bearing on the main focus of our work, the regulation of ENaC by DGK.

Our computational results point to the conclusion that DGK inhibition and the consequent decrease in PI(4,5)P_2_ levels moderate ENaC gain of function in CF by compensating for the higher number of ENaC channels induced by ASL dysregulation. Also, there is strong indication that this regulatory mechanism is mediated through the regulation of PIP5KI by PA. While altering the levels of PI(4,5)P_2_ could seem to be an interesting therapeutic target, caution is necessary as such alterations will probably have unpredictable and possibly undesirable side effects. For instance, Balla’s work^15^ shows that PI(4,5)P_2_ influences many proteins in the cell. In addition, alterations in the levels of PI(4,5)P_2_ obviously lead to changes in other phosphoinositide levels, such as PI(3,4,5)P_3_, which could have further ramifications, such as alterations in the AKT signaling pathway, to name just one. As an alternative, our work suggests that controlling PIP5KI activity or PA levels could achieve therapeutic benefits.

A particularly promising aspect of this research is the recent evidence that phosphoinositides act locally^15,50^ and are present in different pools with different functions^35,51^. This fact could possibly be exploited to create safe and effective therapies for CF and other diseases. Of course, before new therapeutic candidates are pursued, we need to understand far better how these local activities interact and how they are controlled. Such a deeper understanding will probably be gained through targeted combinations of future experimentation and modeling.

## Methods

The equations and parameters of the ENaC—ASL module used here are exactly the same as those presented in^29^. The way in which the phosphoinositide model was designed is flexible enough to simulate membrane patches from different compartments if supporting data are available. For example, it is easy to block or greatly slow down a given reaction a priori if we know that it is not present in a particular compartment. Alternately, one would obtain very low enzyme activities for some reactions as a direct result of the parameter optimization to fit experimental observations. The expansion of the phosphoinositide pathway model introduced some new parameters that were not used in the earlier model^24,29^. For some of these new parameters it was possible to retrieve values from public databases (Table S2). The remaining parameters were adjusted to fit phosphoinositide steady-state values and other phenomena observed experimentally (described in^24^ and Table S1). The adjustment was performed gradually, first with manual adjustments, followed by Nelder-Mead optimization for each dataset separately, and finally using a hybrid genetic algorithm to identify the parameter set with the best fit for all calibration datasets [details in Supplements (Section 1)]. The extended phosphoinositide model was validated by comparing simulations with different datasets [Supplements (Secttion 1); Figures S3, S4, S5 and S6]. The combined model was validated using data from Almaça et al.^21^ on the activity of ENaC with or without DGK inhibition (Fig. 4).

### Mathematical framework

A dynamical model of phosphoinositide metabolism was recently designed within the framework of Biochemical Systems Theory (BST)^52–58^, using ordinary differential equations (ODEs) in the format of a generalized mass action (GMA) system. In this approach, each ODE describes the dynamics of a dependent variable *X*_*i*_, which is formulated as a sum of all fluxes that are directly related to this variable; furthermore, each flux v_i→j_ is formulated as a power-law function, as shown in Eq. (1).

1 $$ \begin{aligned} \frac{{dX_{i} }}{dt} = \sum\limits_{s} {n_{s \to i} .v_{s \to i} - \sum\limits_{p} {m_{i \to p} .v_{i \to p} } } \hfill \\ v_{i \to j} = \gamma_{i \to j} \cdot E_{i \to j} \cdot X_{i}^{{f_{i \to j} }} \hfill \\ \end{aligned} $$

Each quantity *γ*_*i*→*j*_ or *E*_*i*→*j*_ represents the rate constant or enzyme activity for a given flux, respectively, *f*_*i→j*_ is the kinetic order, and *n*_*s*→*i*_ and *m*_*i*→*p*_ are the stoichiometric coefficients for the influxes and effluxes. Each function *v* could contain additional modulators, again as power-law functions.

### Model design, equations and parameters estimation

The model proposed here is a functional integration of two sub-models. The first in an extension of a phosphoinositide pathway model that was recently published, along with pertinent information regarding equations and parameter values^24^. The phosphoinositide model was mostly calibrated with information from the BRENDA^59^ and GENECARDS^60^ databases. As is typical, not all parameter values were available in these databases or the literature, and the missing values were therefore determined with a genetic algorithm such that the optimized model replicated data retrieved from the literature (cf. complete description in^24^). Influxes were associated with pools that have known sources outside the plasma membrane. For instance, PI is supplied from the ER via Nir2^61^, and PI(4)P is delivered by vesicles from the Golgi and PI3P from vesicles from endosomes. The extension of the model furthermore includes influxes into PA from PLD and DAG. In the case of DAG, the PLC independent influx was necessary to maintain the levels of this lipid when PLC was not active.

The second sub-model captures the dynamics of ENaC—ASL and is described in^29^. All but two parameters of the ENaC—ASL regulation model where found by constraining the system using biological data from the literature. The two remaining undetermined parameters were determined through optimization^1^. Both models were designed within the framework of Biochemical Systems Theory (BST)^52,54–56,62,63^.

The main coupling point between the two sub-models is PI(4,5)P_2_, which functionally connects the phosphoinositide pathway with the dynamics of ENaC. The coupling suggests slight modifications to the previous phosphoinositide model^24^ which, in the final form, is depicted in the upper box of Fig. 1; the fluxes and equations are presented in Table 1, and parameters and initial values are given in Table 2. Table S1 of the Supplements summarizes experimental findings, which we used for parameter estimation, and references for parameters can be found in Table S2, as well as in our previous papers^24,29^.

In total, the two combined models have 15 dependent variables and 231 parameters and independent variables. We account for 25 enzymes, including three additions to the model (PLC, DGK and phosphatidate phosphatase). Phosphatidylserine (PS) was added as an independent variable. These new additions added 33 new parameters. Four new parameters were added to account for PIP5KI regulation (f_4→45_PA_, f_4→45_PI45P2_, f_0→45_PA_, f_0→45_PI45P2_). The ENaC/ASL model accounts for 2 dependent variables and 8 parameters.

### Modified Suretekar’s phosphoinositide pathway model

To explore the feasibility of Almaça’s hypothesis regarding the regulation of ENaC by DGK, we used a model that in some sense represents a coarse alternative to our combined model. This model was proposed by Suratekar and colleagues^31^ and uses data from photoreceptor cells of *Drosophila melanogaster*. One must caution that this model, although addressing the same phosphoinositide pathway, may have features that are not entirely representative of human cells.

Suratekar and colleagues tested many versions of their phosphoinositide pathway model and ultimately decided on one that was in accordance with all data available to them. It contains an open cycle with influx of PA into the ER and efflux of DAG from the plasma membrane, but does not include PA regulation of PIP5KI. We used this version, implemented with Michaelis–Menten kinetics, as proposed by the authors.

In the original publication^31^, Suratekar’s model was considered to be sufficient to explain experimental observations if it satisfied the following five criteria: 1. in the wild-type steady state, the levels of the lipid intermediates (relative to PI_total_) lie within 15% of the values given in Fig. 5d; 2. in the case of ten-fold under-expression of DAGK, corresponding to the rdgA3 mutant, the steady-state DAG/PI_total_ ratio should change by less than 15% compared to the wildtype value of this ratio; 3. under ten-fold underexpression of LAZA, corresponding to the laza22 mutant, the steady-state DAG/PI_total_ ratio should change by less than 15% compared to the wildtype value of this ratio; 4. under ten-fold underexpression of DAGK, the steady-state PA_total_/PI_total_ ratio should change by less than 15% compared to the wild-type value of this ratio; 5. under ten-fold underexpression of LAZA, the steady-state PA_total_/PI_total_ ratio should increase by 2.5-fold (± 15%) compared to the wild-type value of this ratio.

The steady-state levels of our model and Suratekar’s do not completely agree. In order to successfully link Suratekar’s and our ENaC-ASL model, we therefore divided the PI(4,5)P_2_ level by its steady-state value and multiplied it by 10,000. In this way, the steady state of PI(4,5)P_2_ becomes similar to the one considered by our model.

For the model to be able to replicate Almaça’s observations of DGK regulation of ENaC, we must make three alterations. The first is an implementation of the enzyme activity of PA where *V*_*max*_ for PIP5KI is substituted with a Hill function (Eq. 2) which, in effect, makes the production of PI(4,5)P_2_ dependent on the levels of PA.

2 $$ \begin{aligned} V_{\max ,pip5KI} & = 0.3 + \frac{{V_{\max ,pip5KI*} \times PMPA^{2} }}{{K_{{M,pip5KI^{*} }}^{2} + PMPA^{2} }} \\ & = 0.3 + \frac{{1.51 \times PMPA^{2} }}{{{0}{\text{.01672982}}^{2} + PMPA^{2} }} \\ \end{aligned} $$

The shift constant 0.3 guarantees that PIP5KI is still active without PA, *K*_*M*_*,*_pip5KI*_ corresponds to the steady-state level of PMPA (Plasma Membrane PA) and *V*_max,pip5KI*_ was calculated for *V*_*max,pip5KI*_ to have the same value as in the original model at the steady state of PMPA. We substituted *V*_*max,pip5KI*_ in the flux equation for PIP5KI by this new expression.

Because this alteration would cause an explosive increase of PI(4)P when DGK is inhibited, we added an efflux from the PI(4)P pool. The efflux is shown in Eq. (3) and included in the differential equation for PI(4)P as a negative term.

3 $$ {\text{V}}_{PI4P\_exit} = 0.08 \times{\text{PI}}\left( {4} \right){\text{P}} $$

The value for the rate constant in this very small flux was obtained by trial and error until the model exhibited a behavior consistent with experimental observations. We are not aware of any direct biological evidence of a significant efflux of this type but it seems reasonable to assume that every phosphoinositide pool should have some efflux representing the phospholipids that exit the plasma membrane by vesicle or non-vesicle transport. It is not clear how relevant this postulated efflux is for the phosphoinositide pools, but we have implemented effluxes in our phosphoinositide pathway model.

Finally, although the steady-state levels of the model are not drastically perturbed by these alterations, we compensated for the new exit of material from the system by increasing the ER PA source flux by 1.1%. Again, trial and error were used to determine this parameter.

One should note that we are not trying to find an optimized set of parameter values for the model. Our objective is solely to test whether Suratekar’s model can qualitatively replicate Almaça’s observations when regulation of PIP5KI by PA is included.

### Model implementation

The model was implemented in the programming language R v3.1.0^64^ together with the package deSolve^65^. We used the ODE integration function with the LSODA method. The code used to implement the models is available in the Github repository with the URL “https://github.com/dolivenca/PI_ENaC_model”.

## Supplementary information

Supplementary information
