# Supplementary material for: ENaC regulation by phospholipids and DGK explained through mathematical modeling
Source: Sci Rep. 2020 Aug 18;10:13952. doi: 10.1038/s41598-020-70630-w (PMC7435262; doi:10.1038/s41598-020-70630-w)
Supplement: Supplementary file 1 — Supplementary information [file 41598_2020_70630_MOESM1_ESM.pdf]

<u>**Supplements**</u>

ENaC regulation by phospholipids and DGK explained through mathematical modeling

Daniel V. Olivença, Eberhard O. Voit, Francisco R. Pinto

1. Expansion of the Phosphoinositide Pathway Model

1.1. Motivation

Information pertaining to our original phosphoinositide pathway model (Figure S1) was presented in [1]. The model mainly addresses concentrations of phosphoinositides and changes in their amounts in response to numerous perturbations of the pathway. These perturbations usually affected enzymes and their activators and inhibitors or consisted of alterations to influxes or effluxes of the system. The results of these experiments are summarized in the supplementary information of [1]. It reproduces several phenomena described in the literature that characterize the pathway, even for phosphoinositides present in small quantities. Notably, however, this model does not account for ENaC or other elements that are needed to study the regulation of this channel.

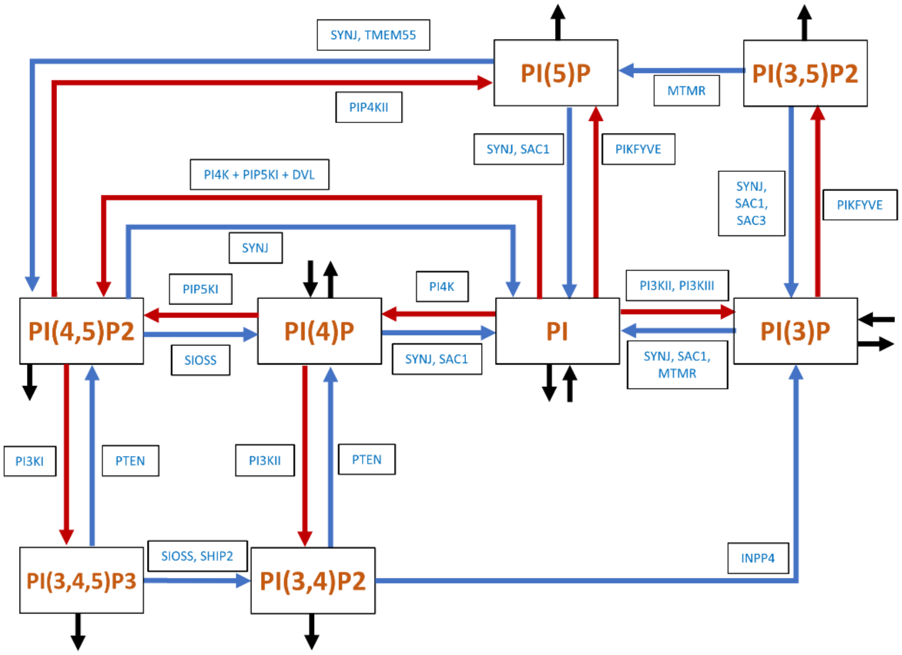

Figure S1. Map of the phosphoinositide pathway. Red arrows represent fluxes of phosphorylation and blue arrows fluxes of hydrolysis. Black arrows represent influxes and effluxes of material entering and leaving the system. SIOSS is a group of phosphatases, consisting of SYNJ 1/2, INPP5 B/J/E, OCRL1, SAC2 and SKIP. PI4K + PIP5KI + DVL denotes a complex formed by the three proteins. Proteins separated by commas catalyze the same reaction. SYNJ: Synaptojanins; INPP5: Inositol polyphosphate 5-phosphatases; OCRL1: Lowe Oculocerebrorenal Syndrome Protein; SAC2: Suppressor of actin; SKIP: Skeletal muscle and kidney enriched inositol polyphosphate phosphatase.

The fluxes $V_{0\rightarrow45}$ and $V_{45\rightarrow0}$ were originally unknown but included to allow the model to replicate the observation that PI(4,5)P_2 levels can be maintained even with low PI(4)P levels (Balla, 2013; Hammond et al., 2012). However, there is now biological evidence for the existence of both, as explained in our original paper about the phosphoinositide pathway model (Olivença et al., 2018).

The new additions to the model result in data fits of a similar quality as for the simpler version of the model, but allow us to assess a host of new features. In particular, they permit assessments of the effects of alterations in phosphoinositides on the dynamics of ENaC, which provide further details on the interactions between PI, PI(4)P, PI(5)P, PI(4,5)P_2, PI4K and PIP5KI. Thus, we connect this model to a module containing all biochemical components that are necessary for studying ENaC regulation by PI(4,5)P_2. Specifically, we add phospholipase C (PLC), which cleaves PI(4,5)P_2 into DAG and inositol triphosphate (IP_3), and allows for competitive inhibition of this phospholipase by PI(4)P and PS [2]. In addition to DAG and IP_3, we explicitly define the enzyme DGK, which facilitates the transformation of DAG into PA. We also add phosphatidate phosphatases (LLPs) that hydrolyze PA back to DAG.

A new input flux of material to the PA pool now accounts for the transformation of PC into PA by phospholipase D (PLD). We opted for this implementation because PC exists in abundance in the cell membrane, with approximately 3,000,000 molecules/μm^2, which corresponds to approximately 25% of the cell membrane [3], and will therefore never be significantly depleted by the action of PLD. It also has a structural role in the cell membrane, and severe depletion of PC causes the cell to lyse. The flux rate constant is adjusted to include the contribution of PC.

We also include the observed activation of PLD by PI(4,5)P_2 [4,5] by letting the levels of phosphoinositide influence the conversion of PC into PA. This activation creates a positive feedback between PI(4,5)P_2 and the production of PA by PLD, which enables the system very elegantly to increase the sensitivity of PI(4,5)P_2 production to alterations in PA when PLC is active (Figure 6). This mechanism appears to do the following: PA has two main sources. It may be created from PC by PLD, or it may be derived from the cleavage of PI(4,5)P_2 by PLC. When PLC displays low activity, a good amount of PA is being produced by PLD. However, when PLC is activated, more PA is produced via degradation of PI(4,5)P_2. Less PI(4,5)P_2 reduces the activation of PLD and leads to lower PA production from PC. Not only does this dual mechanism contribute to the stability of PA levels, but it also alters the ratio between PA coming from PLC and PA coming from PLD.

The combined model moreover accounts for the activation of PIP5KI by PA (Figures 1, 2 and 6 in the main text), a regulation frequently described in the literature [6–8]. This regulation renders PI(4,5)P_2 production dependent on DGK and allows us to study the activity of ENaC under different DGK levels of activation.

In addition to including these mechanisms, we introduce three minor refinements. First, we separate some enzymes catalyzing the same reaction into different fluxes when there is sufficient information to do so. For instance, we define two groups of the myotubularin phosphatase family: MTMR_1_6_14, which corresponds to myotubularins 1 to 6 plus 14, and MTMR78, which contains myotubularins 7 and 8. We cannot really separate these groups further into individual enzymes because no information is available for specific differences among MTMR's. For the same reason, ORCL1, INPP5 B/J, SKIP and SAC2 are grouped into O_I_SK_SA2. Outside these cases, the enzyme separation allows a better understanding of the mechanics in the model and a more accurate replication of experimental perturbations to the pathway, especially with respect to explorations that involve the inhibition or activation of enzymes.

Finally, we account for substrate competition in accordance to observations from wet lab experiments that reported the existence of multiple substrates, especially in the case of phosphatases [9]. The regulation

among PTEN, PI3KI, $PI(4,5)P_2$ and $PI(3,4,5)P_3$ is implemented by creating pools of active (PTENa and PI3KIa) and inactive (PTENc and PI3KIc) PI3KI and PTEN and allowing the products of these enzymes to activate the enzymes. Parameters for this part of the model were found by fitting the model behavior to data reported by Feng *et al.* [10], which were not used in the earlier phosphoinositide model.

As for the earlier models, rate constants and kinetic orders are derived from enzyme kinetic information provided by the BRENDA database [11] or from the literature, after appropriate unit transformations. In this model, K_M values are given in molecules/μm^3, specific activities in molecules/min/mg of enzyme and enzymes in mg. Details can be found in Table ST4 of our previous paper [1]. The kinetic parameters of enzymes used only in the combined model are presented in Table S2. Enzyme activities and transport fluxes were manually set to approximate reported phosphoinositide steady-state values. In some cases, the rate constants were estimated based on data fits.

The parameters were derived from different data sources. Initially, we manually adjusted the parameters to fit the data coarsely. Next, for each dataset, a local, general-purpose optimization function implemented in the R language was used to search for the best fit; specifically, the Nelder–Mead method was used. In the final step, a hybrid genetic algorithm was implemented in the GA package to search for the global minimum.

1.2. PIP5KI activation by PA

PIP5KI is the enzyme responsible for phosphorylating the fifth carbon on the PI(4)P inositol ring and transforming it into $PI(4,5)P_2$. It has two identified binding sites for PA in the C-terminal domain [8]. PIP5KI also has two binding sites for its substrate, PI(4)P, and these have different functions. One allows inhibition of the enzyme, whereas the other is a catalytic site that phosphorylates PI(4)P into $PI(4,5)P_2$. According to Jarquin-Pardo *et al.*, [8], the catalytic site has a K_M of 134 µM in the absence of PA, which is much higher than the inhibitory site with 2.4 µM. Consequently, the enzyme is inactive or in a reduced activity state if the concentration of PA is low. In the presence of PA, the situation is reversed: the K_M for the catalytic site is 2 µM and the K_M for the inhibitory site is 4.2 µM. The values of the k_{cat} for both these cases are 16.4 and 17 min^{-1}. They suggest that PA will mainly alter the affinity of the catalytic site for PI(4)P.

To identify a function that describes the PIP5KI activation by PA, we consulted three papers containing data on $PI(4,5)P_2$ production under different levels of PA [6,8,12]. These articles were described before, and we focus here specifically on PIP5KI activation. Unfortunately, all three studies used Triton X-100, which Jones *et al.* [13] describe as greatly inhibiting the basal lipid kinase activity. We explored different means of compensating for this inhibition. In Moritz' case, we altered the basal activity of PIP5KI from 1 to 1.5 molecules/min/unit of PIP5KI and scaled the data in such a manner that the maximum activation was 3, as reported by Jenkins and colleagues [7]. We achieved this scaling by multiplying the values of $PI(4,5)P_2$ production with 3/20. For Jarquin-Pardo's data, we performed two transformations of the data. First, we altered the basal activity of PIP5KI from 0.2 to 1.5 molecules/min/unit of PIP5KI. Second, we scaled the data so that the maximum activation was again 3. In this case, we achieved this scaling by multiplying the values of $PI(4,5)P_2$ production with 3/30. Finally, for Jenkins' data, we altered the basal activity of PIP5KI to 1.5 molecules/min/unit of PIP5KI and scaled the data so that the maximum activation was again 3. In this case, we multiplied the values of $PI(4,5)P_2$ production with 2.2/13.

Creating a PIP5KI PA activation function

Because data presented by Moritz and Jarquin-Pardo both suggest a sigmoid function for the activation of PIP5KI by PA, we chose a shifted Hill function. This function (Eq. 1) has a K_M of 10,000, a Hill coefficient of 2 and a maximal fold-activation of PIP5KI of 2; it is furthermore shifted by 1.

$$\text{PIP5KI\_activation}\left(PA\right) = 1 + \frac{2 \times PA^2}{10000^2 + PA^2} \qquad (1)$$

These settings ensure that the maximum activation of the PIP5KI enzyme is 3-fold (4.5 molecules/min/unit of PIP5KI), which is in line with the range of 2.2 and 3-fold activation reported by Jones *et al.* [13] and Jenkins *et al.* [7]. When PA has a concentration of 10,000 molecules/μm^3, the enzyme is activated at half the maximum rate, and this value is within the physiological range for PA [3,6,14]. The Hill coefficient of 2 agrees well with the two reported binding sites for PA [8].

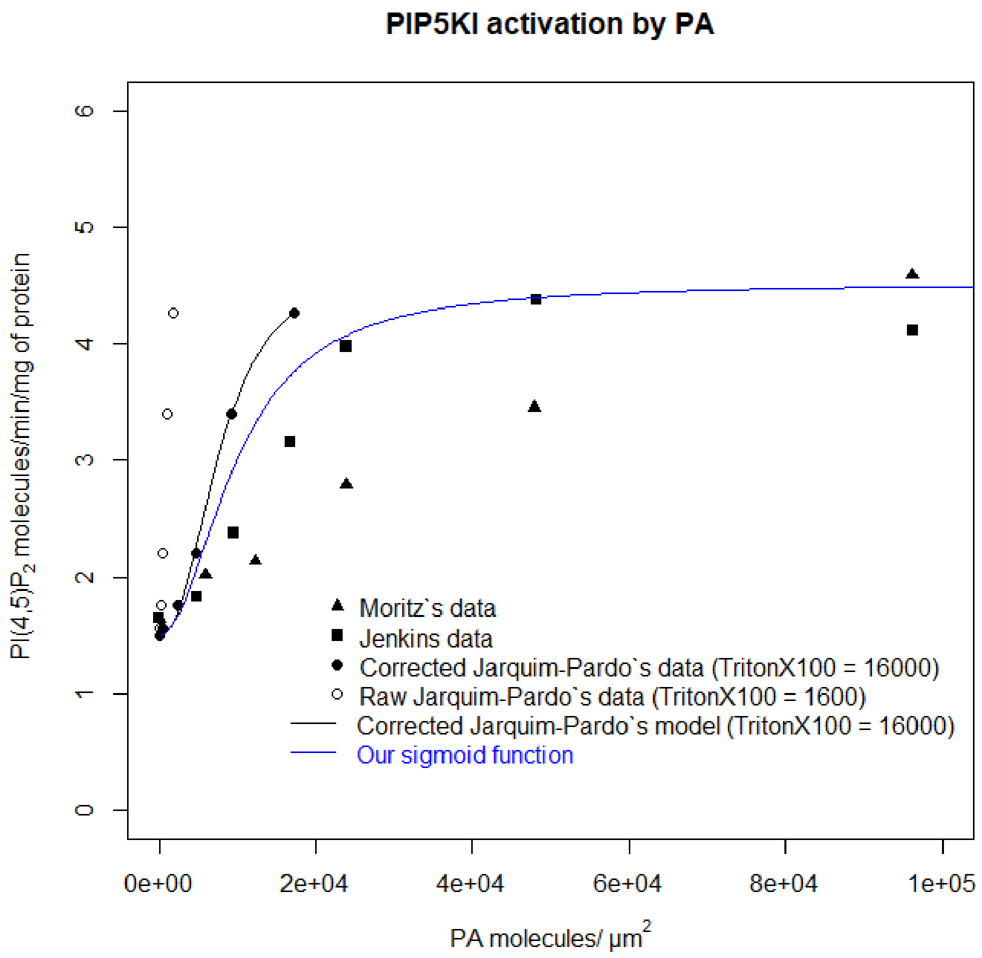

Figure S2. Data and model results regarding PIP5KI activation by PA. Model results are shown as lines and points represent data. Black symbols represent data used to estimate the sigmoid function in the model, which is shown in blue. Grey points represent Jarquin-Pardo's data with the estimated correction for the use of Triton X-100.

Moritz' data

Moritz *et al.* [6] studied PIP5KI activation as a function of PA concentration (Figure S2) using Triton X-100 which, as Jones *et al.* [13] report, greatly inhibits the basal PIP5KI activity. This inhibition causes the reported basal activity to be lower and the apparent fold change activation to be increased. The authors report a basal activity of 1 pmol/min and a maximum activation of the enzyme of 20-fold. We adjusted for the effects of the detergent, as described below.

Jarquin-Pardo's data

Jarquin-Pardo and colleagues [8] reported on an impressive amount of work addressing PIP5KI kinetics and its activation by PA. Unfortunately, they also used Triton X-100. Their data on PIP5KI activation as a function of PA concentration can be seen in Figure S2. The authors report a basal activity of 0.2 molecule/min/unit of PIP5KI and a maximum activation of the enzyme of 30-fold.

To determine the range of PA concentrations in Jarquin-Pardo's experiments, we estimated the total concentration of Triton X-100 they used. Our calculations suggested that a pure Triton X-100 solution at 0.1% volume will have a concentration of 1,600 µM. Because the Triton X-100 was in a mixture, we searched for a number that would produce a better agreement between Jarquin-Pardo's and Moritz' data. We found that a Triton X-100 concentration of 35,000 µM would produce good agreement between the two datasets and was close to the calculated value. Furthermore, although we do not know the exact figure for PA levels, different sources report them as close to the levels of PI(4)P and PI(4,5)P_2 [3,6,14], which is in good agreement with Moritz' data.

Jenkins' data

Jenkins *et al.* [12] studied PIP5KI activation by PA with and without Triton X-100. Unfortunately, they only showed the data for the experiment with the detergent. The authors report a maximum activation of the enzyme of 8, 15 and in some cases even 50-fold with Triton X-100. For the experiment without the detergent they report that the maximum activation was around 3-fold, which agrees with reports by Jones and colleagues. The data points attributed to Jerkins *et al.* in Figure S2 are the data points of the experiment with Triton X-100 scaled down such that the maximum activation of PIP5KI corresponds to the maximum activation observed in the experiment without Triton X-100.

Other pertinent data

Pochynyuk *et al.* [15] provided information about the concentrations of PI(4,5)P_2 under different levels of PLC activity and measured 15 minutes after the perturbations occurred. We used this information, as well as the levels of DAG, PA and IP_3, for the parameterization of PLC activity in the model.

The paper by Sampaio *et al.* [3] contains values for PA and DAG levels that correspond to 0.5% – 1% of total cell lipids in polarizing MDCK cells. This level makes them similarly rare as PI(4)P and PI(4,5)P_2, which are found at around 10,000 molecules/µm^2 in our models, which replicate a 1 µm^2 cell membrane patch [1,16]. PA and DAG values were set in the model to 5,600 and 7,800 molecules/µm^2, respectively.

Studies on platelets [14,17] suggest that the levels of IP_3 are between 300 to 700 molecules/µm^3; the model uses a value of 500 molecules/µm^3. This value was achieved by enabling fast removal of IP_3 from the system, which is in agreement with the fact that IP_3 is a soluble molecule.

Some components of the system display a large dispersion of parameter values, especially in specific enzyme activity. For instance, according to James *et al.* [18], PLC is inhibited by many detergents commonly used in laboratory experiments, specially Triton X-100. James *et al.* used dodecylmaltoside, which was shown not to affect the activity of PLC. They report values between 0.11 and 0.18 µM for the K_M and between 31.3 and 38.9 µmol/min/mg for the specific activity. We decided to use these parameter values but deemed the activity of PLC too strong. As a correction, we considered that PI(4)P competes for PLC, as shown by Ginger, Seifert and others [19,20] and that PLC is inhibited by PS [20]. Values for PS were taken from Sampaio *et al.* [3].

Parameter values from BRENDA

Several papers cited in the BRENDA database [11] suggest very different values for the kinetic parameters of PLC. In particular, values for the K_M of human PLC are listed between 0.0123 and 0.0391 mM, and values for specific activity range between 0.5784 and 200 µmol/min/mg. For *Rattus norvegicus* the ranges are also quite large, namely, between 0.006 and 0.182 mM for K_M and between 3.1 and 204.1 µmol/min/mg for specific activity.

Similarly, it is not clear which parameters for PI3KI that are listed in BRENDA are most appropriate here, and we are therefore not fully confident about the representation of the function of this enzyme. The large amount of enzyme necessary to balance the powerful action of PTEN could be an indication that the values retrieved from BRENDA are not entirely suited for our purposes.

For our model calibration, we ensured that all pertinent values fell into the ranges provided by BRENDA and obtained specific values through various fitting procedures.

1.3. Validation of the Expanded Phosphoinositide Pathway Model

Several datasets were retrieved from the literature and used to fit and validate the extended phosphoinositide pathway module. The same tests used in the previous version of the phosphoinositide pathway model [1] were also applied to the new version and the results are summarized in the first section of Results of the main text and in Figure 3.

PI(4,5)P_2 dynamics in PLC perturbations

Xu *et al.* [21], Gericke *et al.* [22] and Purvis *et al.* [14] state that PI(4,5)P_2 recovers rapidly from an initially steep decrease in response to activation of PLC. The activation of PIP5KI by PA contributes to this recovery [6–8]. These reports do not quantify the magnitude of the recovery or the magnitude of PLC activation that allows a recovery, but it is known that a strong activation of PLC causes PI(4,5)P_2 depletion [23].

The model confirms PI(4,5)P_2 recovery right after PLC activation. Figure S3 exhibits the time course of PI(4,5)P_2 after a two-fold activation of PLC and the subsequent recovery. The original parameter set allows only a modest recovery while PLC is active, but a combination of stronger PA / PIP5KI activation and an adequate activation of PLC produces very clear PI(4,5)P_2 recovery.

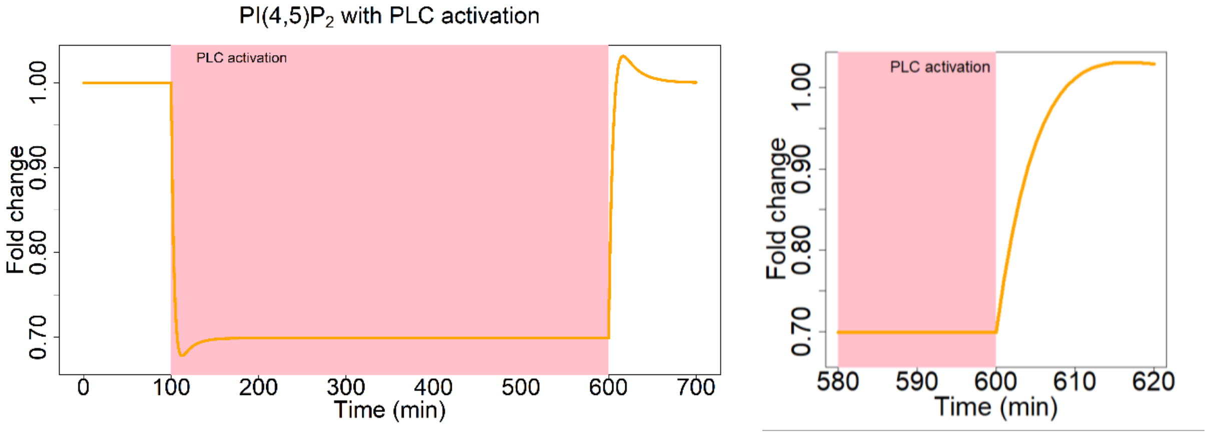

Figure S3. PI(4,5)P_2 recovery after two-fold activation of PLC. Left panel: PLC cleaves PI(4,5)P_2 into DAG and IP_3. Several authors report a recovery of PI(4,5)P_2 for different magnitudes of PLC activation [14,21,22]. The model shows recovery of PI(4,5)P_2 after a two-fold increase in PLC action (shaded). Right panel: Recovery of PI(4,5)P_2 after termination of PLC activation. PLC was activated 2-fold and caused a 30% reduction on PI(4,5)P_2. At time 600 min PLC activation ended, and PI(4,5)P_2 took approximately 10 minutes to return to steady state level.

The recovery of PI(4,5)P_2 after termination of PLC activation is much slower than reported in the literature. Specifically, Figure S3 suggests that the recovery of PI(4,5)P_2 after the end of a two-fold activation of PLC takes approximately 10 minutes (reaching half of the recovery in 2.5 minutes), according to our model. To resolve the discrepancy between the model and the data, we followed Falkenburger *et al.* [2] and Xu *et al.* [21] , who accelerated the production of PI(4,5)P_2, without actually mentioning a particular mechanism or rationale. Nevertheless, this speed-up resolved the discrepancy in time scale in their models.

The creation of PI(4,5)P_2 can follow alternative routes, and we chose to accelerate the direct transformation of PI into PI(4,5)P_2. To maintain the level of the lipid at the observed steady state value, we compensated by very slightly increasing the efflux of the PI(4,5)P_2 pool.

In the top panel in Figure S4, with the previously mentioned changes, the model can now simulate PI(4,5)P_2 recovery during PLC activation (black dots in the top panel in Figure S4 represent data from Chang *et al.* [24]), with fast recovery after the end of PLC activation. Even with this accelerated PI(4,5)P_2 production, the model faithfully replicates the data from Almaça *et al.*, namely, the normalization of ENaC activity when DGK is inhibited (Figure S4 bottom).

Unfortunately, these parameter alterations compromise the fit of model simulations with other experimental observations. PLC consumes PI(4,5)P_2, so our intuition assumes that if we activate PLC, PI(4,5)P_2 should decrease. It is possible to set PLC activations that are small enough so that the consumed PI(4,5)P_2 will be less than the PI(4,5)P_2 produced by the increase in PA and its activation of PIP5KI. As a consequence, small PLC activations cause the level of PI(4,5)P2 to increase. Because of this secondary effect, we did not use the PI(4,5)P_2 production acceleration. Importantly, the speed of recovery is not important for the main focus of our analysis.

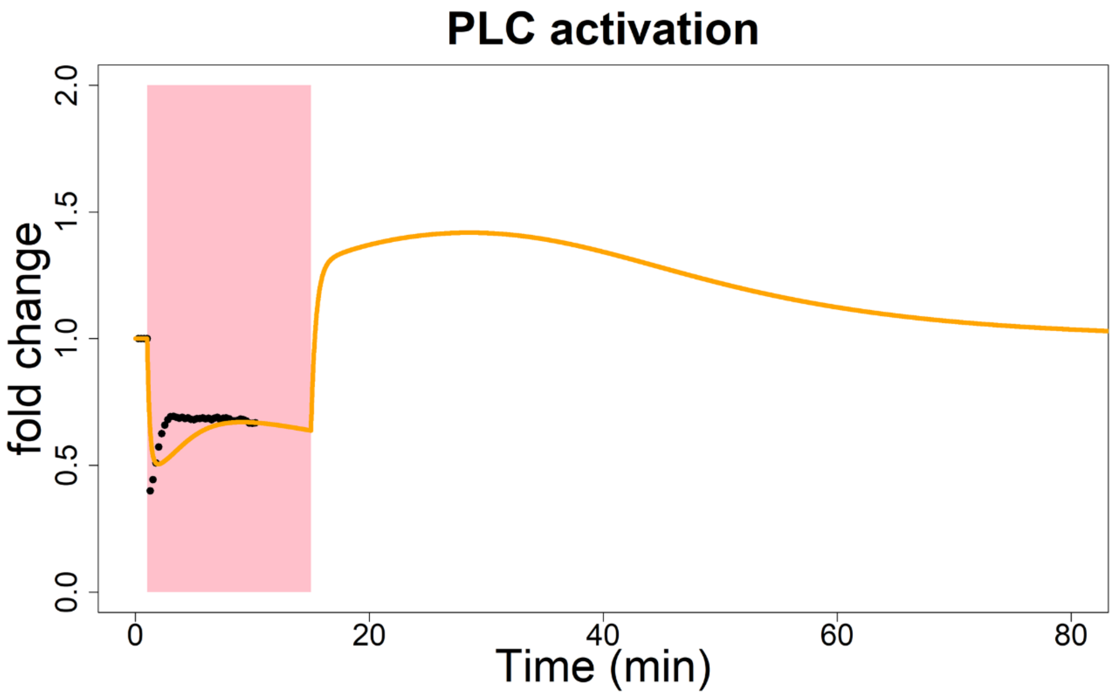

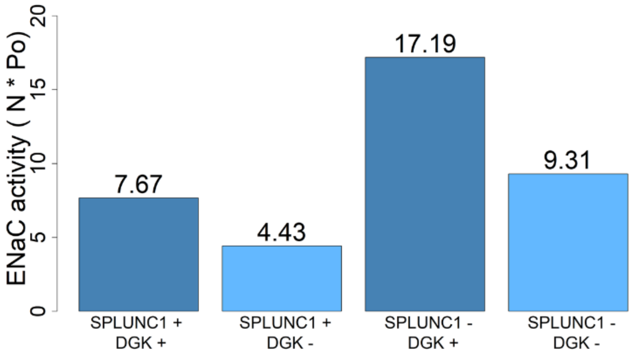

Figure S4. PI(4,5)P_2 recovery with PLC activation in the model where PI(4,5)P_2 production is accelerated. (Top) PLC is activated in the period highlighted in pink. With acceleration of the PI(4,5)P_2 production, the recovery during and after PLC activation is similar to records in the literature: the black dots are data from Chang *et al.* [24]. (Bottom) The model with acceleration of PI(4,5)P_2 production still replicates Almaça et al. ENaC / DGK data.

To the best of our knowledge, there are no reports in the literature about this behavior. It is possible that the model actually predicts the effects of PLC on PI(4,5)P_2 correctly but that researchers did not report them due to the small scale of the lipid fluctuations, considered them as artifacts produced by their experimental setup, or hesitated to mention them because the observations were contradictory to their intuition. Also, IP_3 causes the release of Ca^{2+} from the ER reservoir, which in turn activates PLC, which secondarily increases the production of IP_3. We did not add Ca^{2+} to the model because it would bring

considerable complexity to this work and distract from our main message. Yet, it is possible that the missing components exert regulation suppressing these artifacts.

Interestingly, Antonescu *et al.* [25] reported a similar behavior between PLD and PA. They explain it by suggesting a negative feedback mechanism where the PA produced by PLD could negatively regulate the more efficient DGK PA production. They also highlight the fact that certain isoforms of DGK are negatively regulated by PA.

Both versions of the model replicate Almaça's observations of ENaC activity with DGK inhibition. Therefore, this discrepancy in the time scale of PI(4,5)P_2 recovery after a PLC activity pulse does not affect our conclusions about the relevance of the PA/PIP5KI activation for the impact of DGK inhibition on ENaC activity.

If the activation of PIP5KI by PA is sufficiently strong, the model system becomes bistable, and the levels of the dependent variables do not return to the initial steady state, even if PLC activation is returned to its initial level. Instead, the system becomes locked into a state of high PA levels that increase PI(4,5)P_2 production so much that the basal activity of PLC produces enough PA to keep the high PA levels and maintain the new steady state. This model result could be an artifact because PLC saturation would prevent high DAG and PA production only as a consequence of a great increase in PI(4,5)P_2. However, if this bistability could be demonstrated in reality, it could be an effective way to maintain the signal after the initial stimulus has ceased. Interestingly, Purvis *et al.* [14] encountered the same phenomenon with their model.

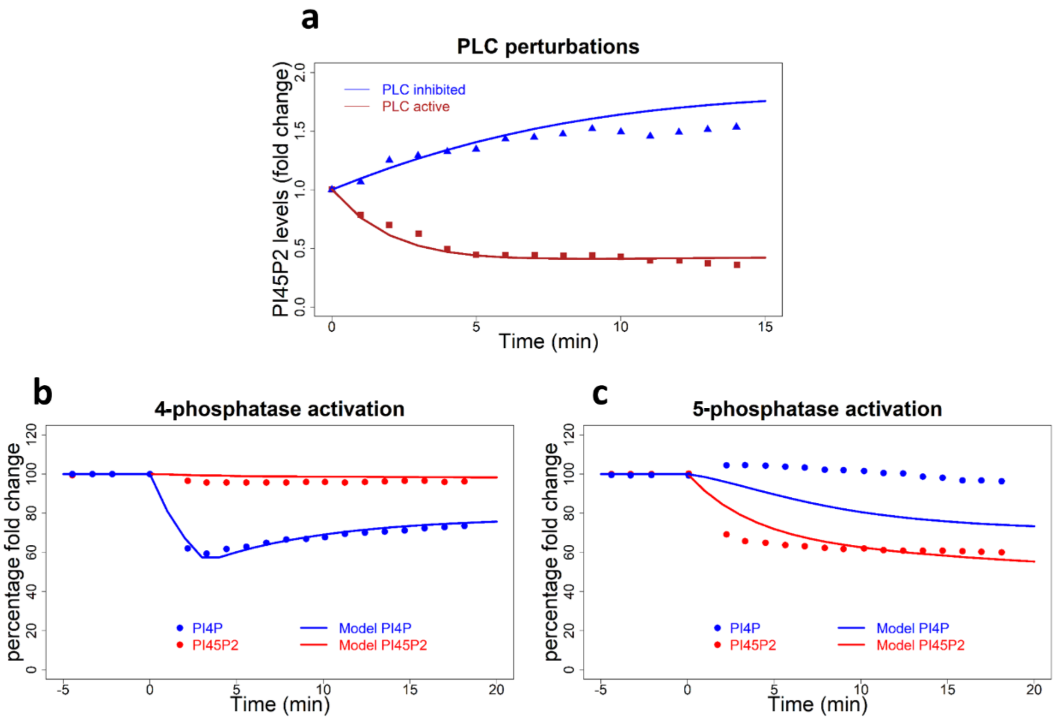

Figure S5. Perturbations that affect PI(4)P and PI(4,5)P_2. a) PLC was inhibited (blue) or activated (red) and levels of PI(4,5)P_2 were measured. Triangles and squares represent data points [15] and lines the model time courses where PLC was inhibited to 1% or activated to 4-fold of the enzyme's basal activity. **b)** PI(4)P and PI(4,5)P_2 time course in a 4-phosphatase activation. Points represent data [26] and lines show model results. **c)** PI(4)P and PI(4,5)P_2 time courses following 5-phosphatase activation. Points represent data [26] and lines show model results.

Pochynyuk *et al.* [15] measured the levels of PI(4,5)P_2 for the first 15 minutes after altering PLC activity. The data and model results can be seen in Figure S5a. The model presents a good fit to these data. Furthermore, while no data exist for later time points, the simulation can be extended and shows that, after about 500 minutes, PI(4,5)P_2 reaches a steady state that is increased to 1.6-fold for PLC inhibition and decreased to 0.4-fold for a 4-fold activation of PLC activity in comparison to the basal level.

Degradation of PI(4)P and PI(4,5)P_2

Várnai *et al.* [26] presented data using reporters for PI(4)P and PI(4,5)P_2 that derived from two experiments: one where SAC1 4-phosphatase was used to degrade PI(4)P and another where the INPP5E 5-phosphatase was used to degrade PI(4,5)P_2. For the SAC1 experiment, our model produced a good fit (Figure S5b) with an initial 8-fold increase in the model's SAC1 activity followed by attenuation to a 2-fold activation after 4 minutes.

Our model was initially unable to reproduce the INPP5E results when we used perturbations of a reasonable magnitude. This failure was probably due to the fact that we had underestimated the amount of enzyme in the cell membrane or the enzyme activity. Raising the activity of the enzyme 30-fold, we obtain noticeable alterations to the PI(4)P and PI(4,5)P_2 levels. Furthermore, the shape of the data seems to suggest that the chimera INPP5E used by Várnai *et al.* was, in addition to its 5-phosphatase ability, able to hydrolyze the 4-phosphate of PI(4,5)P_2. To test this speculation, we increased the activity of the SYNJ phosphatase, which is able to hydrolyze the 4th and 5th position of the inositol ring in PI(4,5)P_2. With this alteration, the model led to a qualitatively reasonable time course (Figure S5c). Zhong *et al.* [27] showed that Sac1 and other phosphates can have an allosteric effect which can essentially increase/decrease the Sac1 activity based on substrate concentration. The model results suggest that this may also be the case for INPP5E.

Perturbations in PTEN, PI3KI, PI(4,5)P_2 and PI(3,4,5)P_3

For completeness, we discuss fitting the earlier phosphoinositide model to data reported by Feng *et al.* [10] and Pochynyuk *et al.* [28], which indicated that the specific activity of PTEN must be greater than the value retrieved from the enzyme database BRENDA [11]. This discrepancy is in line not only with Feng's conclusions, but also with reports by McConnachie *et al.* [29] and by Johnston and Raines [30]. We proceeded by using the values presented by Johnston and Raines [30] and also adjusted the amounts of total and active PI3KI to obtain an adequate quantity of PI(3,4,5)P_3.

The data fits from our model are not particularly good, which is at least partially due to the fact that the system we are interested in is the *apical* part of the plasma membrane of bronchial epithelial cells, which is characterized by a lack of PI(3,4,5)P_3. Nonetheless, we show the results of this simulation.

Feng *et al.* [10] presented two time courses that resulted from first activating PI3KI with rCD1 at time point t=5 min and then inhibiting the enzyme with FK506 at time point t=40 min. We simulated these perturbations with a 28-fold increase in the activation flux of PI3KI at t=5 min and a return of PI3KI to its steady-state values at t=40 min (Figure S6a). Figure S6b shows results of blocking PTEN activity with H_2O_2 at t=2 min and inhibiting PI3KI with FK506 at t=8 min. We simulated these perturbations with a decrease of 50% in PTEN at t=2 min and a decrease of PI3KI of 30% values at t=8 min. The simulation results reflect the observations quite well.

Pochynyuk *et al.* [31] present a PI(3,4,5)P_3 time course where PI3KI is inhibited with LY294002. We simulated this perturbation with a 90% decrease in PI3KI (Figure S6 c). Again, our model was able to reproduce this experimental observation.

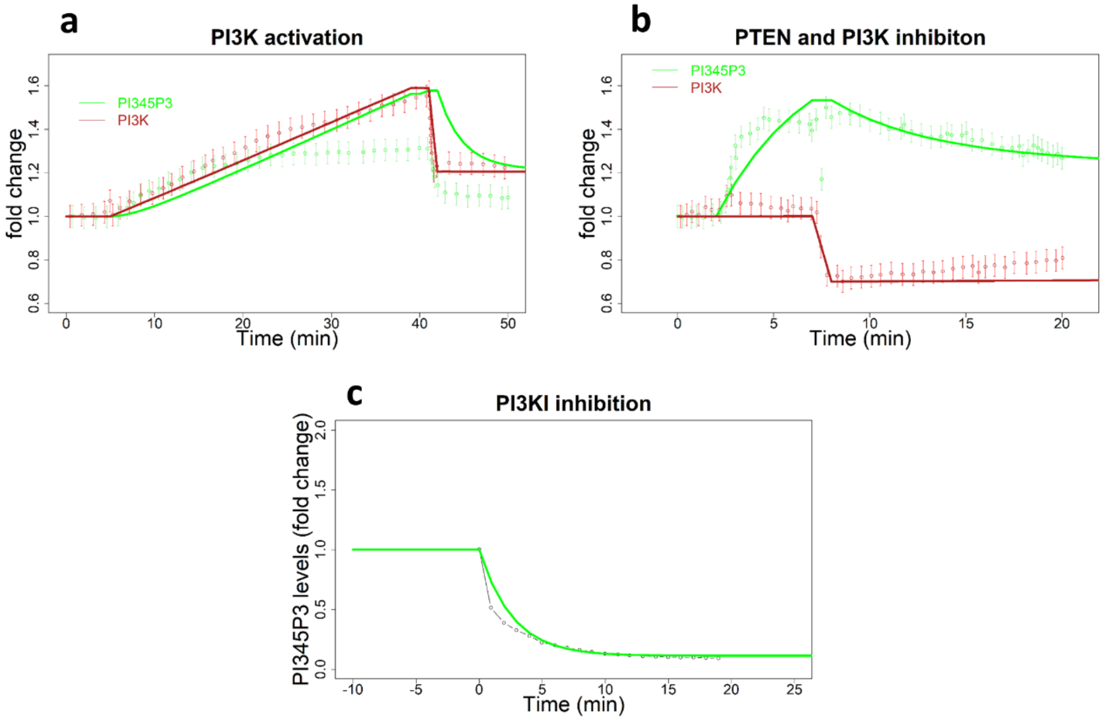

Figure S6. Perturbations that affect PI(3,4,5)P_3. Panel a: PI3KI and PI(3,4,5)P_3 time courses in response to enzyme activation at t = 5 and inhibition at t = 40. Circles and intervals represent data [10], lines are model results. **Panel b:** PI3KI and PI(3,4,5)P_3 time courses in response to PTEN inhibition at t = 3 and PI3KI inhibition at t = 8. Circles and intervals represent data [10], lines are model results. **Panel c:** PI(3,4,5)P_3 time courses in a PI3KI inhibition. Black circles represent data [31] and green line shows model results.

While the fit in Figure S6a is not particularly good, Figures S6b and 6c are much better. It is true that the data suggest the existence of a mechanism that limits the increase in concentration of PI(3,4,5)P_3 when it reaches about 120% of its normal level, but we are unaware of what this mechanism could be. Even so, we considered it pertinent to show Figure S6a to convey an impression of the limitations of the model. One should note that PI(3,4,5)P_3 is not fundamental for our system which addresses the apical part of the plasma membrane of bronchial epithelial cells, which is characterized by a lack of PI(3,4,5)P_3. In a future work, we intend to pay attention to the study of the regulation of ENaC by PI(3,4,5)P_3, which is expected to improve the fit of the model to these particular data.

1.4. Previous modelling efforts to simulate the Phosphoinositide pathway.

Other models have been proposed to simulate the phosphoinositide pathway or at least parts of it. Falkenburger, Dickson and Hille [2,23] studied the kinetics of PI(4)P, PI(4,5)P_2, PLC, DAG, IP_3 and calcium. They showed that an acceleration of PI(4,5)P_2 production during PLC activation is necessary. If PI(4,5)P_2 production is not accelerated, PLC action will fade or stop. This study did not account for the activation of

PIP5KI by PA, although it considered an increase in PI4K function when PLC was activated, which has a similar effect.

Narang *et al.* [32] developed a mathematical model that takes the activation of PIP5KI by PA into account. However, this model oversimplifies the phosphoinositide pathway by representing PI, PI(4)P, PI(4,5)P_2 and PI(3,4,5)P_3 with just one variable and implementing PA activation of PIP5KI with an auto-activation of the phosphoinositides.

Purvis *et al.* [14] built a model of the pathway in platelets. While generally impressive, this model does not account for all pertinent phosphoinositide species and does not include the activation of PIP5KI by PA.

PI is phosphorylated twice to become PI(4,5)P_2: first by PI4K and afterwards by PIP5KI. The limiting rate of this process seems to be PI4K phosphorylation, which is 20 times slower than the phosphorylation of PIP5KI. In an attempt to explain this difference, Falkenburger, Dickson and Hille [2,23] suggested that an acceleration of the production of PI(4,5)P_2, which would compensate for the depletion caused by PLC, should impact PI4K. We cannot fully agree with this rationale because of the following observations. PIP5KI is inhibited by its substrate, PI(4)P, which Falkenburger's argument ignores. Specifically, Jarquin-Pardo *et al.* [8] state that PIP5KI has an inhibitor as well as an activator binding site for PI(4)P and that, in the absence of PA, the K_M for the inhibitor site is 50-fold smaller than the K_M for the activator site. These authors furthermore state that "the addition of PA increases the affinity with which the active site of mPIP5K-Ib binds PI(4)P by 67-fold." Furthermore, Moritz *et al.* [6] report that the PIP5KI activation by PA is inhibited by PI(4,5)P_2. These two pieces of evidence suggest that PIP5KI only functions fully in the presence of PA and with reduced levels of PI(4,5)P_2. Finally, PI4K has PI as its substrate, which is 300 times more abundant than PI(4)P, the substrate for PIP5KI. These observations suggest that PIP5KI is the rate limiting component of PI(4,5)P_2 production.

Falkenburger *et al.* [2] used in their model an amount of PI(4)P that is less than a third of the quantity of PI(4,5)P_2 and assumed that two thirds of PI(4,5)P_2 are in a bound state while all PI(4)P is free. We kept the amounts of these two phosphoinositides similar, in accordance with what is commonly accepted [33,34].

2. Extended Results

2.1 The combined model with PIP5KI activation by PA replicates the effects of DGK knockdown.

ENaC control by DGK

Almaça *et al.* [35] performed siRNA screens to identify modulators of ENaC activity and noticed that DGK inhibition reduces ENaC activity to WT basal levels in CF F508del cells, but does not have a significant effect in healthy (WT) cells. Also, in CF, DGK has no effect on ENaC when PLC is activated or inhibited.

To explore these findings with our model, we started by studying the effects of SPLUNC1 and DGK perturbations. We simulated CF conditions by setting SPLUNC1 and the CFTR dependent flux (V_5) equal to zero. As a result, the number of ENaC channels (*N*) increases 2.25 times relatively to the WT, which is close to the value reported by Tarran's group [36]. Concerning ENaC activity, ENaC is projected to be 2.25 more active in CF than in WT (Figure 4). It should be noted that, in the model, the increase of ENaC activity under CF conditions is due to an increase in the number of channels. If we inhibit DGK in the CF setup, *N* is not affected but, due to the drop in P_o, the activity of ENaC decreases to about half. This finding suggests that the moderation of ENaC by DGK inhibition is obtained by a different mechanism, such a s alteration

of the channel-open probability, rather than the one being affected in the disease, namely the number of channels.

Comparison between WT conditions with and without DGK inhibition

Almaça *et al.* [35] showed that there are no significant differences between WT conditions with and without DGK inhibition.

To estimate the magnitude of DGK inhibition we used the information in the first paragraph of Almaça el *al.* [35]. On page 1394 they say: "First, we showed that a chemical blocker of DGK (DGKinh, 25 mM) led to 50% ENaC inhibition in A549 cells (Figures 4A and 4B)." We used this information to estimate the level of DGK inhibition and tested it in WT simulations of our model. The results can be seen in Fig. 4 of the main text. We choose 25% as a good substitute for values with several decimal places that would have yielded perfect 50% inhibition of ENaC.

The model, by contrast, indicates a clear drop when DGK is inhibited, which reduces the activity of ENaC by roughly 40%. Even so, these model predictions fall well within the confidence intervals of the data (Figure 4). This effect is caused by a decrease in P_o and a slight increase in *N*; the estimated drop in ENaC's activity is mainly due to the reduction in P_o.

Comparison between WT and CF with DGK inhibition

Almaça *et al.* [35] report that inhibiting DGK in CF normalizes ENaC activity, *i.e.*, the ENaC activity in these situations is similar to WT. The model yields some differences between WT, WT \ DGK- and CF \ DGK- cases that should have roughly the same value (Figure 4). However, the original data also show a non-significant difference between WT \ DGK- and the other two cases and all results are well within the confidence intervals in the data, so that we may consider these model results as an adequate reflection of the data. Overall, the model predicts a DGK-induced reduction of ENaC activity under CF conditions that is similar to the basal activity in WT.

Comparison between ENaC activity with and without DGK inhibition in CF, while accounting for inhibition of PLC

Almaça *et al.* [35] found no significant differences for these cases. Confirming this result, the corresponding model settings produce the same value for the two situations (Figure 4). Specifically, the model replicates a 1 μm^2 cell membrane patch where $PI(4,5)P_2$ basal levels are around 10,000 molecules/μm^2 [1,16]. When PLC is inhibited, the estimated levels of $PI(4,5)P_2$ are around 16,000 molecules/μm^2, which corresponds to an increase of approximately 50% over the basal level. The levels of PA, and even more so of DAG, are greatly reduced to 2,600 and 42 molecules/μm^2, which corresponds to 33% and 1% of their basal levels, respectively. The severe reduction of DAG explains the lack of effect of DGK's inhibition over ENaC activity. With such small amount of substrate, the level of enzyme activity becomes irrelevant.

In the data from Almaça *et al.*, the value for the PLC- scenario has the same magnitude as the unperturbed CF state (CF). This discrepancy is presumably due to the fact that Almaça *et al.* used a fluorescence essay where the marker was probably saturated for values higher than in the case of CF.

Some discrepancies remain when PLC is perturbed. In the case of PLC inhibition (CF\PLC-), Almaça's data indicate the same magnitude as in the unperturbed CF state (CF), while the model predicts a much higher value. This discrepancy is again presumably due to the fact that Almaça *et al.* used a fluorescence essay where the marker was probably saturated for values around 100% of ENaC activity. Nonetheless, the model is able to replicate the data associated with PLC inhibition at least qualitatively.

Comparison between CF with and without inhibition of DGK, while accounting for activation of PLC

According to Almaça's results, CF / DGK-, CF / PLC+ and CF / PLC+ / DGK- have similar values of about 50% ENaC activity in comparison with the unperturbed case of CF. These results are similar to those from the model (Figure 4). The model is able to replicate the CF / PLC+ case quantitatively; however, when the kinase is inhibited (CF / PLC+ / DGK-), a slightly more pronounced reduction in ENaC activity is predicted by the model. This reduction is probably due to missing regulation that is not yet described in the literature and therefore not implemented in the model, which suggests the need of more detailed studies of PLC and DGK. Notwithstanding this slight discrepancy, the model captures the qualitative behavior of the biological system quite well.

2.2. The combined model with phosphoinositide recycling, but without PIP5KI activation by PA, does not replicate the effects of a DGK knockdown.

The model allows us to test the hypothesis of Almaça and colleagues that the moderation of ENaC activity by DGK is caused by a general decrease in phosphoinositides, due to their reduced recycling. To assess this hypothesis, we alter the extended phosphoinositide model in two ways. First, we allow the efflux from the PA pool, $V_{PA\rightarrow}$, to supply the PI pool with material, which simulates the transformation of PA into PI in the ER. This additional, simplified step in a crude way closes the circle of phosphoinositide recycling. Because the efflux of PA is only 5% of the influx of PI, we maintain the already present influx into the PI pool, which is now reduced by an amount equal to the PA efflux. The flux $V_{\rightarrow 0}$ in this model extension can be interpreted biologically as the amount of PI produced in the ER from PA that did not originate in the plasma membrane; expressed differently, it represents PA created *de novo*. Second, we remove the regulation of PIP5KI by PA.

With these presumably reasonable settings, the model does not replicate the observation by Almaça and colleagues: if DGK is inhibited, it no longer affects ENaC activity, whether in WT or CF (Figure S7 a). The reason is that the DGK independent $V_{\rightarrow 0}$ flux is much greater than that DGK dependent flux $V_{PA\rightarrow}$.

To remedy the situation, it would be necessary to balance the input into PI and the efflux out of PA. Specifically, the input into PI would have to be an exact multiple of the efflux $V_{PA\rightarrow}$ out of PA such that it would be equal to the original PI influx. Such a coupling would make the PI pool dependent on the PA efflux. In a simulation with these settings, the ENaC activity is more sensitive to DGK inhibition, but the decrease in the activity is very small (Figure S7 b).

If the inhibition of DGK in the model is made stronger, this configuration of the model can actually replicate the data reported by Almaça and colleagues (Figure S7c). However, to achieve this configuration, PI influx would have to change proportionally to the PA efflux, which is 20 times smaller. Another possible explanation could be that the levels of PA and PI in the plasma membrane would somehow have to be tightly coordinated. There is no evidence of this degree of coupling between the PI influx and the PA efflux, and while there are no exact measurements for these lipids in the plasma membrane, all existing cell measurements suggest that PI is 10 times more abundant than PA [3,37]. Moreover, it is known that PA is produced *de novo* in the ER, and this production affects the sensitivity of PI to plasma membrane PA. Thus, the model does not support the hypothesis of Almaça *et al.* [35] that inhibiting DGK brings the recycling of the phosphoinositides to a halt, which in turn decreases $PI(4,5)P_2$ and $PI(3,4,5)P_3$ and causes reduced ENaC activity.

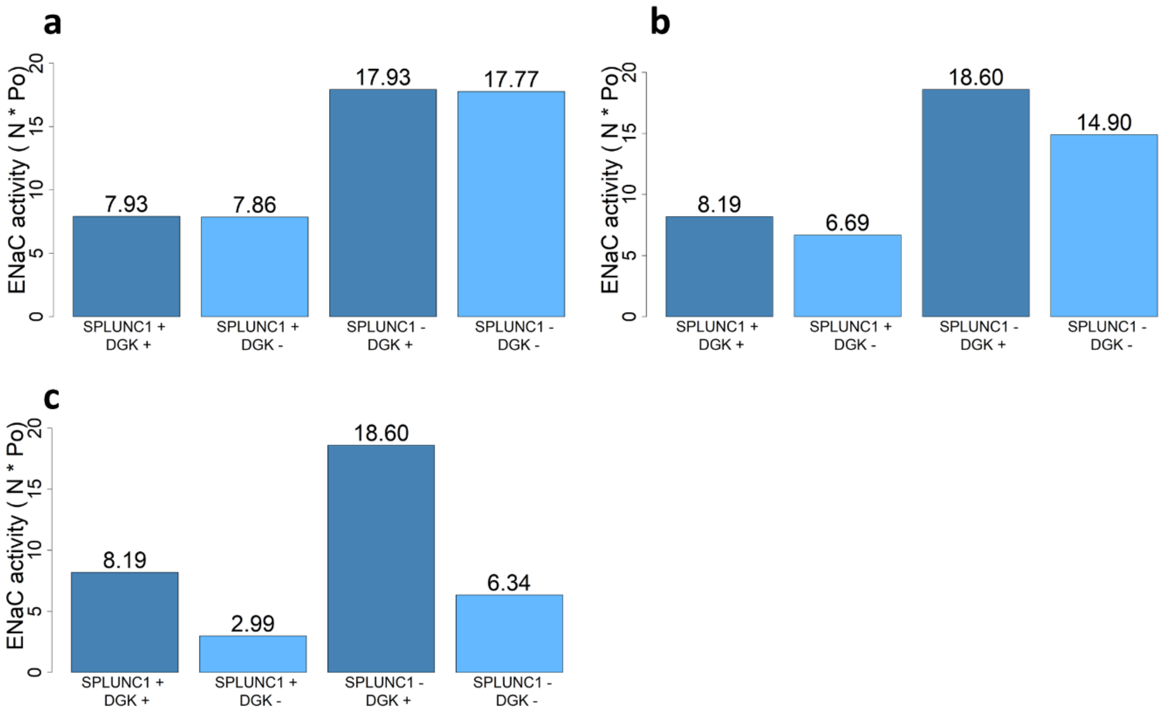

Figure S7. Extended phosphoinositide model, allowing simulations of PI recycling without PIP5KI regulation by PA. Plots represent ENaC activity in WT (SPLUNC1 +) and CF (SPLUNC1 -) conditions, with and without DGK inhibition. To test Almaça's hypothesis, we altered the extended phosphoinositide model to simulate phosphoinositide recycling and removed the regulation of PIP5KI by PA. **Panel a:** Under these conditions, the model does not replicate the observation of ENaC moderation when DGK is inhibited, as reported by Almaça and colleagues. **Panel b:** Making the influx of PI a multiple of the PA efflux, the model does not replicate the observation of Almaça and colleagues either. **Panel c:** With the same conditions as in b), and additional 75% inhibition by DGK instead of the usual 25%, the model does replicate the observation of Almaça and colleagues. However, this degree of sensitivity of PI to plasma membrane PA efflux is contrary to empirical evidence.

2.3 Sensitivity Analysis

The results of a comprehensive sensitivity analysis are presented in Table S3. The box at the lower right corner of this Table displays the sensitivities of ENaC and ASL to changes in independent variables from the ENaC / ASL model presented in Olivença *et al.* (2019) [16]. As expected, these values are similar because this model was adopted without alterations.

Many of the high sensitivities are associated with the new additions to the model (PLC, DAG , PA), which is consistent with the observation that signaling systems may present high sensitivities [38].

As in Olivenca *et al.* (2018), we found high sensitivities with respect to the $V_{0 \rightarrow 45}$ flux parameters which, by affecting $PI(4,5)P_2$, affect all other variables that are heavily dependent on lipids, including ENaC and ASL. In this new model, but not in the previous version, we also found high sensitivities with respect to the $V_{45 \rightarrow 0}$ parameters.

The presented results are not sensitive to alterations in the initial conditions. The steady state is stable and given enough time, the system returns to this steady state. ENaC, ASL and PI(3,4,5)P_3 are slower to recover.

2.4 Considerations about PI(4)P accumulation in Suratekar *et al.* model

In the fourth section of Results in the main text we link our ENaC / ASL model to the model of the recycling of phosphoinositides created by Suratekar et al. When DGK was inhibited, it caused a sizable increase in PI(4)P. To mitigate this we created a small efflux in the PI(4)P pool that function much like as escape valve, and prevent the dramatic increase. Here we discuss this situation further.

There was the possibility that a PIP5KI isoform insensitive to PA could function as the PI(4)P escape valve. But according to Sasaki *et al.* (2009), all PIP5KI isoforms are activated by PA. The phosphatidylinositol phosphate kinases type II (PIP4KII) transform PI(5)P into PI(4,5)P2. It is possible that a PA insensitive phosphorylation of PI(4)P occurs but there is no evidence for this insensitivity in the literature.

However, as a thought experiment, let's imagine the suggested PA-insensitive phosphorylation of PI(4)P does exist. To simulate this situation, we transform the PI(4)P escape valve flux into a flux from PI(4)P to PI(4,5)P_2 that is insensitive to PA, thereby creating two PI(4,5)P_2 influxes, one sensitive and the other insensitive to PA. The steady state is very slightly altered but the difference is at the order of 10^-4. Let's perturb the system. Reducing the Vmax of DGK to 20% does yield a similar dynamics in comparison to the system with the PI(4)P escape valve. But can this system show a dynamics like the one reported by Almaça *et al.*. Namely, will the value of PI(4,5)P_2 drop and inhibit ENaC activity, when DGK is inhibited? The answer is no (Figure S8). The extra PI(4,5)P_2 produced by the PA-insensitive phosphorylation of PI(4)P is enough to maintain ENaC activity. Thus, a system with PA-insensitive phosphorylation of PI(4)P does not replicate the data from Almaça *et al.*

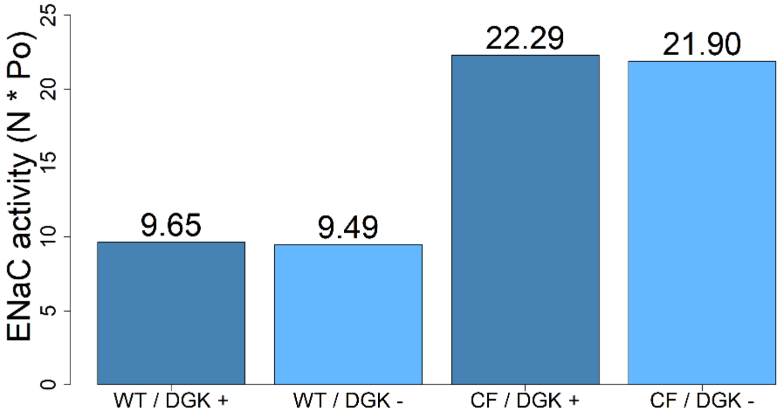

Figure S8. Creating a parallel PI(4)P to PI(4,5)P2 flux that is insensitive to PA will not reproduce the data reported by Almaça *et al.* that ENaC activity will decrease when DGK is inhibited.

Nonetheless, these results made us revisit our implementation of Suratekar's model. We realized that the escape valve flux works so well because it follows a first-order kinetics, and therefore does not saturate. What happens if the process saturates? Assuming a Michaelis-Menten representation, the answer depends

on the Vmax of that flux. If it is high enough it prevents the system from being flooded with PI(4)P. Because an escape valve with an inadequate Vmax would not accomplish its task, we can assume that Vmax is relatively high.

What happens if the PA-insensitive phosphorylation of PI(4)P saturates as well? Let's assume the PA-insensitive phosphorylation of PI4P flux is similar to the PI(4)P escape valve at steady state, that a Michaelis-Menten model is a good representation of its dynamics and that the Km is equal to that of the PA sensitive phosphorylation flux of PI(4)P. If so, the results are again a very similar to the steady states of the original system. As before, the extra PI(4,5)P_2 produced is sufficient to maintain the levels of ENaC activity when DGK is inhibited.

These results are not favorable to the hypothesis of the existence of a PA-insensitive phosphorylation of PI(4)P. However, we should be aware that other possibilities can cause the PI(4)P overload, like Sac1 activity or additional regulators of PIP5KI, just to name a few.

3. Supplementary Tables

Table S1 : Observed experimental phenomena used to calibrate and validate the model performance

	Phenomenon	Model
1	In the apical part of the cell membrane, inhibiting PLC should cause an increase of 50 % in $PI(4,5)P_2$ in the first 15 min. [15]	Figure S5 a, blue line and points.
2	In the apical part of the cell membrane, PLC activation will decrease $PI(4,5)P_2$ by 50% in the first 15 min. [15]	Figure S5 a, red line and points.
3	Intense activation of PLC can lead to 75% [22] or 90% [39] of $PI(4,5)P_2$ depletion.	Increasing 7 times the basal activity of PLC will deplete $PI(4,5)P_2$ by 73%, but PLC must be increased 20 times to deplete $PI(4,5)P_2$ by 90%.
4	Inhibition of class I DGK in BSC-1 cells results in a 50% reduction in total PA levels, indicating the majority of cellular PA is synthesized by type I DGKs. [25]	Inhibiting DGK by 75% will reduce PA in 52%. Knockout of DGK will reduce PA by 75%.
5	Time series data for activation and inhibition of PI3KI and PI(3,4,5)P3 dynamics. [10]	Figure S6 a, b.
6	Time series data on PI3KI inhibition with LY294002 and readings of PI(3,4,5)P3 levels in a cell-attached patch made on a principal cell from a collecting duct freshly isolated from a salt restricted rat. [31]	Figure S6 c.

Table S2 : Enzymes added to the phosphoinositide model and their characteristics

Model enzyme	Enzymes	Flux	Values	Reference
DGK	DGK	$V_{DAG \rightarrow PA}$	K_m = 0.25<br>SA = 2.207	[40,41]
LPP	phosphatidate phosphatase	$V_{PA \rightarrow DAG}$	K_m = 0.277<br>SA = 0.1175	[42,43]
PLC	PLC β/γ/δ/ε/η	$V_{45 \rightarrow DAG}$	K_m = 0.00014<br>SA = 35	[8,13]
		$V_{4 \rightarrow DAG}$	K_m = 0.000255<br>SA = 12.7	[8,13]
PLD	PLD I/II	$V_{PC \rightarrow PA}$	*estimated*	

With regard to the ENaC model, our previous paper explored two models: one where PI(4,5)P_2 protected ENaC from ubiquitination and another where this was not the case. As we did not detect significant differences in results, we chose here the simpler model, where PI(4,5)P_2 does not protect ENaC from ubiquitination. The equations, parameters and initial values for this model are presented in [16]. While most of the information regarding the two sub-models was presented somewhere, the connection of the two models requires additional effort. Most of this effort concerns the dynamics of PI(4,5)P_2.

Table S3 : Sensitivity Analysis

	PI	PI3P	PI4P	PI5P	PI35P2	PI45P2	PI34P2	PI345P3	pi_3KI_a	PTEN_a	DAG	IP_3	PA	ENaC	ASL
PI3KI	-7.80E-05	2.14E-05	-8.84E-05	-2.64E-04	7.32E-05	6.79E-04	2.33E-03	2.22E+00	2.12E+00	4.66E-04	5.76E-04	6.65E-04	4.40E-04	-4.80E-04	-1.25E-03
PI3KII	-8.02E-03	8.85E-01	-1.84E-04	8.09E-01	8.89E-01	6.71E-02	1.01E+00	-6.69E-02	-3.78E-02	4.75E-02	5.79E-02	6.55E-02	4.51E-02	-6.18E-02	-1.20E-01
PI3KIII	-1.62E-04	1.79E-02	3.60E-05	1.64E-02	1.80E-02	1.36E-03	1.55E-04	-1.36E-03	-7.70E-04	9.63E-04	1.18E-03	1.33E-03	9.08E-04	-1.26E-03	-2.44E-03
PI4K	-6.32E-02	-9.57E-02	1.09E+00	-5.63E-02	-6.63E-02	9.14E-02	1.06E+00	-9.11E-02	-5.14E-02	6.48E-02	2.13E-01	7.56E-02	1.52E-01	-8.42E-02	-1.63E-01
PIP5KI	-2.66E-02	9.94E-03	-4.12E-01	6.48E-02	2.23E-02	4.31E-01	-3.64E-01	-4.30E-01	-2.43E-01	3.05E-01	3.22E-01	4.26E-01	2.55E-01	-3.99E-01	-7.69E-01
PI4K_PIP5KI	-1.01E-01	-5.42E-02	2.05E-02	8.48E-02	-7.19E-03	9.25E-01	9.49E-02	-9.18E-01	-5.19E-01	6.54E-01	8.02E-01	9.03E-01	6.23E-01	-8.61E-01	-1.65E+00
PIP4KII	-5.72E-04	-3.14E-04	1.09E-04	-1.03E+00	-5.05E-05	8.40E-03	5.03E-04	-8.44E-03	-4.77E-03	5.96E-03	7.28E-03	8.21E-03	5.65E-03	-7.75E-03	-1.50E-02
PIKfyve	-1.44E-03	-8.83E-01	2.91E-04	1.82E-01	1.09E-01	1.52E-02	1.33E-03	-1.53E-02	-8.63E-03	1.08E-02	1.32E-02	1.49E-02	1.02E-02	-1.40E-02	-2.72E-02
PTEN	9.26E-05	-6.92E-05	1.25E-04	2.64E-04	-1.30E-04	-8.05E-04	-1.26E-02	-2.51E+00	-1.44E+00	9.99E-01	-6.81E-04	-7.88E-04	-5.38E-04	6.15E-04	1.45E-03
SYNJ	1.93E-02	-1.49E-03	-2.26E-02	7.85E-02	-1.46E-02	-1.68E-01	-3.50E-02	1.69E-01	9.52E-02	-1.20E-01	-1.48E-01	-1.64E-01	-1.15E-01	1.55E-01	3.01E-01
SAC1	1.67E-02	3.19E-03	-2.79E-01	-4.12E-02	-1.16E-02	-2.81E-02	-2.73E-01	2.81E-02	1.58E-02	-1.99E-02	-5.84E-02	-2.39E-02	-4.22E-02	2.58E-02	5.01E-02
SAC2	3.07E-04	-1.13E-04	4.74E-03	-7.47E-04	-2.56E-04	-4.97E-03	4.21E-03	4.96E-03	2.80E-03	-3.52E-03	-3.71E-03	-4.91E-03	-2.95E-03	4.57E-03	8.86E-03
SAC3	9.38E-07	5.20E-04	1.60E-06	-5.92E-05	-6.40E-05	-1.07E-05	8.05E-07	1.07E-05	6.01E-06	-7.60E-06	-9.19E-06	-1.05E-05	-1.51E-05	1.32E-06	5.49E-06
MTMR1_6_14	1.62E-04	-2.99E-02	-8.34E-05	-7.67E-03	-9.99E-01	-6.68E-04	-2.06E-04	6.69E-04	3.77E-04	-4.74E-04	-5.88E-04	-6.52E-04	-4.64E-04	6.06E-04	1.18E-03
MTMR78	5.58E-05	-6.10E-03	-9.99E-06	-5.58E-03	-6.13E-03	-4.70E-04	-5.13E-05	4.72E-04	2.66E-04	-3.33E-04	-4.08E-04	-4.59E-04	-3.25E-04	4.24E-04	8.26E-04
INPP4	-3.85E-06	3.53E-03	1.38E-05	3.22E-03	3.53E-03	2.70E-04	-2.05E-01	-3.30E-04	-1.87E-04	1.91E-04	2.35E-04	2.63E-04	1.74E-04	-2.57E-04	-4.95E-04
INPP5BJ	2.31E-04	-8.55E-05	3.57E-03	-5.63E-04	-1.93E-04	-3.75E-03	3.17E-03	3.74E-03	2.11E-03	-2.66E-03	-2.80E-03	-3.70E-03	-2.23E-03	3.44E-03	6.67E-03
INPP5E	1.68E-04	4.70E-06	2.59E-03	-4.16E-04	-1.48E-04	-2.72E-03	2.30E-03	2.72E-03	1.53E-03	-1.93E-03	-2.03E-03	-2.69E-03	-1.62E-03	2.50E-03	4.84E-03
TMEM55	8.69E-06	4.65E-06	1.36E-07	1.46E-02	6.51E-07	-1.25E-04	-5.99E-06	1.26E-04	7.11E-05	-8.88E-05	-1.08E-04	-1.22E-04	-9.23E-05	1.07E-04	2.10E-04
ORCL1	7.46E-04	-2.77E-04	1.15E-02	-1.82E-03	-6.23E-04	-1.21E-02	1.03E-02	1.21E-02	6.82E-03	-8.57E-03	-9.04E-03	-1.20E-02	-7.17E-03	1.11E-02	2.16E-02
SKIP	6.10E-04	-2.26E-04	9.43E-03	-1.49E-03	-5.09E-04	-9.88E-03	8.38E-03	9.87E-03	5.57E-03	-7.00E-03	-7.38E-03	-9.77E-03	-5.86E-03	9.09E-03	1.76E-02
SHIP2	5.51E-07	2.74E-07	1.68E-06	-5.67E-07	1.81E-08	-5.86E-06	1.99E-05	-2.48E-05	-1.45E-05	-4.16E-06	-4.98E-06	-5.75E-06	-1.18E-05	-3.16E-06	-3.18E-06
PLC	-2.77E-02	6.26E-04	-3.14E-01	-3.19E-02	1.25E-02	-4.12E-01	-2.65E-01	4.17E-01	2.35E-01	-2.93E-01	6.23E-01	5.97E-01	3.91E-01	3.78E-01	7.36E-01
DGK	-2.51E-02	-8.70E-03	-7.68E-02	2.94E-02	2.96E-03	2.66E-01	-5.29E-02	-2.66E-01	-1.50E-01	1.89E-01	-2.91E-01	2.61E-01	5.41E-01	-2.46E-01	-4.75E-01
LPP	7.53E-04	2.61E-04	2.30E-03	-8.83E-04	-8.87E-05	-7.99E-03	1.59E-03	8.02E-03	4.53E-03	-5.66E-03	8.68E-03	-7.83E-03	-1.62E-02	7.34E-03	1.42E-02
PS	2.67E-02	-5.38E-04	3.02E-01	3.06E-02	-1.20E-02	3.96E-01	2.55E-01	-3.96E-01	-2.24E-01	2.81E-01	-6.00E-01	-5.75E-01	-3.76E-01	-3.67E-01	-7.08E-01
$\gamma_{\rightarrow 0}$	1.09E+00	5.89E-01	3.66E-01	1.69E-01	9.50E-02	3.47E-01	-5.57E-01	-3.46E-01	-1.95E-01	2.45E-01	3.44E-01	3.34E-01	2.63E-01	-3.20E-01	-6.19E-01
$\gamma_{\rightarrow 4}$	3.98E-03	-4.65E-03	1.23E-01	-4.53E-03	-6.37E-03	1.35E-02	1.11E-01	-1.35E-02	-7.63E-03	9.60E-03	2.68E-02	1.17E-02	1.94E-02	-1.25E-02	-2.42E-02
$\gamma_{\rightarrow 3}$	-1.13E-04	9.35E-02	4.25E-04	8.52E-02	9.36E-02	7.31E-03	4.27E-04	-7.33E-03	-4.14E-03	5.18E-03	6.37E-03	7.13E-03	4.94E-03	-6.74E-03	-1.31E-02
$\gamma_{0\rightarrow 3b}$	-7.96E-03	8.80E-01	1.68E-03	8.05E-01	8.84E-01	6.69E-02	7.56E-03	-6.71E-02	-3.79E-02	4.74E-02	5.81E-02	6.54E-02	4.51E-02	-6.17E-02	-1.20E-01
$f_{0\rightarrow 3b\_PI}$	-1.78E-02	1.97E+00	3.76E-03	1.80E+00	1.98E+00	1.50E-01	1.69E-02	-1.50E-01	-8.48E-02	1.06E-01	1.30E-01	1.46E-01	1.01E-01	-1.38E-01	-2.68E-01
$f_{0\rightarrow 3b\_PI4P}$	5.20E-03	-5.74E-01	-1.10E-03	-5.25E-01	-5.77E-01	-4.37E-02	-4.94E-03	4.39E-02	2.48E-02	-3.10E-02	-3.79E-02	-4.27E-02	-2.95E-02	4.02E-02	7.80E-02
$\gamma_{0\rightarrow 3c}$	-1.62E-04	1.79E-02	3.60E-05	1.64E-02	1.80E-02	1.36E-03	1.55E-04	-1.36E-03	-7.70E-04	9.63E-04	1.18E-03	1.33E-03	9.08E-04	-1.26E-03	-2.44E-03
$f_{0\rightarrow 3c\_PI}$	-2.33E-04	2.59E-02	5.11E-05	2.36E-02	2.60E-02	1.96E-03	2.24E-04	-1.97E-03	-1.11E-03	1.39E-03	1.70E-03	1.92E-03	1.31E-03	-1.81E-03	-3.52E-03
$\gamma_{3\rightarrow 0a}$	1.50E-04	-1.65E-02	-2.99E-05	-1.51E-02	-1.66E-02	-1.26E-03	-1.41E-04	1.27E-03	7.16E-04	-8.96E-04	-1.10E-03	-1.23E-03	-8.60E-04	1.15E-03	2.24E-03
$f_{3\rightarrow 0a\_PI3P}$	6.96E-04	-7.69E-02	-1.45E-04	-7.03E-02	-7.73E-02	-5.85E-03	-6.60E-04	5.87E-03	3.32E-03	-4.15E-03	-5.08E-03	-5.72E-03	-3.95E-03	5.38E-03	1.04E-02
$f_{3\rightarrow 0a\_PI4P}$	-6.04E-05	6.74E-03	1.46E-05	6.16E-03	6.77E-03	5.07E-04	5.91E-05	-5.09E-04	-2.87E-04	3.59E-04	4.40E-04	4.95E-04	3.34E-04	-4.75E-04	-9.19E-04
$f_{3\rightarrow 0a\_PI5P}$	2.49E-07	3.34E-05	1.74E-06	2.97E-05	3.33E-05	-3.30E-06	1.44E-06	3.23E-06	1.80E-06	-2.34E-06	-2.76E-06	-3.24E-06	-1.01E-05	-5.52E-06	-7.76E-06
$f_{3\rightarrow 0a\_PI35P2}$	4.49E-07	1.13E-05	1.70E-06	9.50E-06	1.11E-05	-4.98E-06	1.25E-06	4.92E-06	2.75E-06	-3.53E-06	-4.21E-06	-4.88E-06	-1.12E-05	-3.97E-06	-4.76E-06
$f_{3\rightarrow 0a\_PI45P2\_PI4P}$	-5.96E-05	6.66E-03	1.44E-05	6.08E-03	6.69E-03	5.00E-04	5.84E-05	-5.02E-04	-2.84E-04	3.55E-04	4.34E-04	4.89E-04	3.30E-04	-4.69E-04	-9.07E-04
$f_{3\rightarrow 0a\_PI45P2\_PI5P}$	-3.24E-04	3.59E-02	7.03E-05	3.28E-02	3.60E-02	2.72E-03	3.09E-04	-2.73E-03	-1.54E-03	1.93E-03	2.36E-03	2.66E-03	1.83E-03	-2.51E-03	-4.87E-03
$f_{3\rightarrow 0a\_PI345P3}$	5.19E-07	3.52E-06	1.69E-06	2.40E-06	3.28E-06	-5.57E-06	1.19E-06	5.51E-06	3.08E-06	-3.95E-06	-4.73E-06	-5.46E-06	-1.16E-05	-3.43E-06	-3.71E-06
$f_{3\rightarrow 0a\_PI45P2\_PI}$	-1.45E-05	1.67E-03	4.87E-06	1.52E-03	1.67E-03	1.21E-04	1.55E-05	-1.21E-04	-6.86E-05	8.57E-05	1.05E-04	1.18E-04	7.36E-05	-1.20E-04	-2.30E-04
$\gamma_{3\rightarrow 0c}$	2.52E-04	-2.78E-02	-5.14E-05	-2.54E-02	-2.79E-02	-2.12E-03	-2.38E-04	2.13E-03	1.20E-03	-1.50E-03	-1.84E-03	-2.07E-03	-1.44E-03	1.94E-03	3.77E-03
$f_{3\rightarrow 0c\_PI3P}$	1.17E-03	-1.29E-01	-2.45E-04	-1.18E-01	-1.30E-01	-9.82E-03	-1.11E-03	9.86E-03	5.57E-03	-6.96E-03	-8.52E-03	-9.59E-03	-6.63E-03	9.03E-03	1.75E-02
$f_{3\rightarrow 0c\_PI4P}$	-1.45E-04	1.60E-02	3.24E-05	1.47E-02	1.61E-02	1.21E-03	1.39E-04	-1.22E-03	-6.89E-04	8.61E-04	1.05E-03	1.19E-03	8.11E-04	-1.13E-03	-2.18E-03
$f_{3\rightarrow 0c\_PI5P}$	-1.66E-07	7.93E-05	1.83E-06	7.16E-05	7.94E-05	1.93E-07	1.84E-06	-2.68E-07	-1.80E-07	1.37E-07	2.71E-07	1.65E-07	-7.76E-06	-8.73E-06	-1.40E-05
$f_{3\rightarrow 0c\_PI35P2}$	3.10E-07	2.66E-05	1.73E-06	2.35E-05	2.65E-05	-3.81E-06	1.38E-06	3.75E-06	2.09E-06	-2.70E-06	-3.20E-06	-3.74E-06	-1.05E-05	-5.05E-06	-6.85E-06
$\gamma_{3\rightarrow 0d}$	1.78E-04	-1.96E-02	-3.59E-05	-1.79E-02	-1.97E-02	-1.50E-03	-1.68E-04	1.50E-03	8.49E-04	-1.06E-03	-1.30E-03	-1.46E-03	-1.02E-03	1.37E-03	2.66E-03
$f_{3\rightarrow 0d\_PI3P}$	8.25E-04	-9.12E-02	-1.73E-04	-8.34E-02	-9.17E-02	-6.94E-03	-7.83E-04	6.97E-03	3.94E-03	-4.92E-03	-6.02E-03	-6.78E-03	-4.69E-03	6.38E-03	1.24E-02
$f_{3\rightarrow 0d\_PI35P2}$	3.69E-07	2.01E-05	1.72E-06	1.76E-05	2.00E-05	-4.30E-06	1.33E-06	4.25E-06	2.37E-06	-3.05E-06	-3.63E-06	-4.23E-06	-1.08E-05	-4.59E-06	-5.96E-06
$\gamma_{3\rightarrow 0e}$	5.58E-05	-6.10E-03	-9.99E-06	-5.58E-03	-6.13E-03	-4.70E-04	-5.13E-05	4.72E-04	2.66E-04	-3.33E-04	-4.08E-04	-4.59E-04	-3.25E-04	4.24E-04	8.26E-04
$f_{3\rightarrow 0e\_PI3P}$	2.57E-04	-2.84E-02	-5.26E-05	-2.59E-02	-2.85E-02	-2.16E-03	-2.43E-04	2.17E-03	1.23E-03	-1.53E-03	-1.88E-03	-2.11E-03	-1.47E-03	1.98E-03	3.85E-03
$\gamma_{0\rightarrow 4}$	-6.32E-02	-9.57E-02	1.09E+00	-5.63E-02	-6.63E-02	9.14E-02	1.06E+00	-9.11E-02	-5.14E-02	6.48E-02	2.13E-01	7.56E-02	1.52E-01	-8.42E-02	-1.63E-01
$f_{0\rightarrow 4\_PI}$	-2.32E-01	-3.47E-01	4.00E+00	-2.04E-01	-2.40E-01	3.33E-01	3.91E+00	-3.31E-01	-1.87E-01	2.36E-01	7.79E-01	2.76E-01	5.58E-01	-3.08E-01	-5.95E-01
$\gamma_{4\rightarrow 0a}$	9.64E-03	1.47E-02	-1.66E-01	8.64E-03	1.02E-02	-1.40E-02	-1.62E-01	1.40E-02	7.87E-03	-9.91E-03	-3.24E-02	-1.16E-02	-2.33E-02	1.29E-02	2.49E-02
$f_{4\rightarrow 0a\_PI3P}$	-1.87E-05	-2.91E-05	3.34E-04	-1.79E-05	-2.04E-05	2.21E-05	3.25E-04	-2.21E-05	-1.25E-05	1.57E-05	5.99E-05	1.74E-05	3.47E-05	-2.89E-05	-5.31E-05
$f_{4\rightarrow 0a\_PI4P}$	8.85E-02	1.35E-01	-1.52E+00	7.99E-02	9.40E-02	-1.29E-01	-1.49E+00	1.29E-01	7.26E-02	-9.12E-02	-2.98E-01	-1.06E-01	-2.14E-01	1.18E-01	2.30E-01
$f_{4\rightarrow 0a\_PI5P}$	-1.88E-05	-2.92E-05	3.36E-04	-1.80E-05	-2.05E-05	2.23E-05	3.27E-04	-2.23E-05	-1.26E-05	1.58E-05	6.02E-05	1.75E-05	3.49E-05	-2.90E-05	-5.34E-05
$f_{4\rightarrow 0a\_PI35P2}$	-5.93E-06	-9.65E-06	1.13E-04	-6.45E-06	-6.90E-06	3.57E-06	1.10E-04	-3.60E-06	-2.06E-06	2.53E-06	1.68E-05	2.06E-06	3.82E-06	-1.18E-05	-2.00E-05
$f_{4\rightarrow 0a\_PI45P2\_PI4P}$	-3.89E-03	-5.91E-03	6.70E-02	-3.48E-03	-4.10E-03	5.63E-03	6.54E-02	-5.62E-03	-3.17E-03	3.99E-03	1.31E-02	4.65E-03	9.37E-03	-5.19E-03	-1.01E-02
$f_{4\rightarrow 0a\_PI45P2\_PI5P}$	-2.10E-02	-3.19E-02	3.62E-01	-1.88E-02	-2.21E-02	3.04E-02	3.53E-01	-3.03E-02	-1.71E-02	2.15E-02	7.06E-02	2.51E-02	5.07E-02	-2.80E-02	-5.43E-02
$f_{4\rightarrow 0a\_PI345P3}$	-1.39E-06	-2.76E-06	3.52E-05	-2.39E-06	-2.12E-06	-3.00E-06	3.39E-05	2.95E-06	1.64E-06	-2.13E-06	1.59E-06	-3.38E-06	-7.12E-06	-5.79E-06	-8.29E-06
$f_{4\rightarrow 0a\_PI45P2\_PI}$	-9.72E-04	-1.48E-03	1.68E-02	-8.72E-04	-1.03E-03	1.40E-03	1.64E-02	-1.40E-03	-7.91E-04	9.95E-04	3.27E-03	1.16E-03	2.34E-03	-1.30E-03	-2.52E-03
$\gamma_{4\rightarrow 0c}$	1.62E-02	2.46E-02	-2.79E-01	1.45E-02	1.71E-02	-2.35E-02	-2.72E-01	2.34E-02	1.32E-02	-1.66E-02	-5.44E-02	-1.94E-02	-3.91E-02	2.16E-02	4.19E-02
$f_{4\rightarrow 0c\_PI4P}$	1.44E-01	2.21E-01	-2.48E+00	1.31E-01	1.54E-01	-2.10E-01	-2.42E+00	2.10E-01	1.19E-01	-1.49E-01	-4.85E-01	-1.73E-01	-3.48E-01	1.93E-01	3.75E-01
$f_{4\rightarrow 0c\_PI3P}$	-4.53E-05	-6.95E-05	7.92E-04	-4.18E-05	-4.85E-05	6.07E-05	7.73E-04	-6.06E-05	-3.42E-05	4.30E-05	1.49E-04	4.93E-05	9.89E-05	-6.44E-05	-1.22E-04
$f_{4\rightarrow 0c\_PI5P}$	-4.56E-05	-6.99E-05	7.97E-04	-4.20E-05	-4.87E-05	6.11E-05	7.78E-04	-6.10E-05	-3.44E-05	4.33E-05	1.50E-04	4.96E-05	9.96E-05	-6.47E-05	-1.23E-04
$f_{4\rightarrow 0c\_PI35P2}$	-1.49E-05	-2.33E-05	2.68E-04	-1.45E-05	-1.63E-05	1.65E-05	2.61E-04	-1.66E-05	-9.37E-06	1.17E-05	4.70E-05	1.28E-05	2.54E-05	-2.38E-05	-4.32E-05
$\gamma_{0\rightarrow 5}$	-7.83E-04	-4.18E-04	1.62E-04	8.29E-02	-5.46E-05	6.89E-03	7.38E-04	-6.92E-03	-3.91E-03	4.89E-03	5.98E-03	6.73E-03	4.64E-03	-6.36E-03	-1.23E-02
$f_{0\rightarrow 5\_PI}$	-5.57E-03	-2.98E-03	1.14E-03	5.90E-01	-3.88E-04	4.91E-02	5.24E-03	-4.92E-02	-2.78E-02	3.48E-02	4.26E-02	4.79E-02	3.31E-02	-4.52E-02	-8.76E-02
$f_{0\rightarrow 5\_PI3P}$	1.90E-06	9.10E-07	1.40E-06	-1.43E-04	2.92E-08	-1.77E-05	-1.10E-07	1.77E-05	9.95E-06	-1.25E-05	-1.52E-05	-1.73E-05	-1.98E-05	7.73E-06	1.79E-05
$\gamma_{5\rightarrow 0a}$	1.67E-04	8.92E-05	-3.24E-05	-1.76E-02	1.15E-05	-1.47E-03	-1.55E-04	1.48E-03	8.35E-04	-1.04E-03	-1.28E-03	-1.44E-03	-1.00E-03	1.35E-03	2.62E-03
$f_{5\rightarrow 0a\_PI3P}$	2.17E-07	1.27E-08	1.75E-06	3.45E-05	-8.78E-08	-2.89E-06	1.47E-06	2.83E-06	1.57E-06	-2.05E-06	-2.41E-06	-2.85E-06	-9.84E-06	-5.89E-06	-8.48E-06
$f_{5\rightarrow 0a\_PI4P}$	-6.74E-05	-3.61E-05	1.56E-05	7.19E-03	-4.80E-06	5.92E-04	6.50E-05	-5.94E-04	-3.36E-04	4.20E-04	5.14E-04	5.78E-04	3.91E-04	-5.54E-04	-1.07E-03
$f_{5\rightarrow 0a\_PI5P}$	7.79E-04	4.16E-04	-1.58E-04	-8.24E-02	5.42E-05	-6.87E-03	-7.31E-04	6.89E-03	3.89E-03	-4.87E-03	-5.95E-03	-6.71E-03	-4.64E-03	6.31E-03	1.22E-02
$f_{5\rightarrow 0a\_PI35P2}$	4.37E-07	1.31E-07	1.70E-06	1.12E-05	-7.24E-08	-4.84E-06	1.26E-06	4.78E-06	2.67E-06	-3.43E-06	-4.09E-06	-4.75E-06	-1.12E-05	-4.10E-06	-5.01E-06
$f_{5\rightarrow 0a\_PI45P2\_PI4P}$	-6.65E-05	-3.56E-05	1.54E-05	7.09E-03	-4.74E-06	5.85E-04	6.42E-05	-5.87E-04	-3.32E-04	4.14E-04	5.07E-04	5.71E-04	3.86E-04	-5.47E-04	-1.06E-03
$f_{5\rightarrow 0a\_PI45P2\_PI5P}$	-3.61E-04	-1.93E-04	7.55E-05	3.82E-02	-2.52E-05	3.18E-03	3.41E-04	-3.19E-03	-1.80E-03	2.25E-03	2.75E-03	3.10E-03	2.13E-03	-2.93E-03	-5.68E-03
$f_{5\rightarrow 0a\_PI345P3}$	5.15E-07	1.72E-07	1.69E-06	2.91E-06	-6.70E-08	-5.52E-06	1.19E-06	5.47E-06	3.06E-06	-3.92E-06	-4.69E-06	-5.42E-06	-1.16E-05	-3.47E-06	-3.78E-06
$f_{5\rightarrow 0a\_PI45P2\_PI}$	-1.62E-05	-8.78E-06	5.11E-06	1.78E-03	-1.23E-06	1.42E-04	1.69E-05	-1.43E-04	-8.06E-05	1.01E-04	1.23E-04	1.39E-04	8.78E-05	-1.39E-04	-2.67E-04
$\gamma_{5\rightarrow 0c}$	2.80E-04	1.50E-04	-5.55E-05	-2.96E-02	1.94E-05	-2.47E-03	-2.62E-04	2.48E-03	1.40E-03	-1.75E-03	-2.14E-03	-2.41E-03	-1.67E-03	2.27E-03	4.40E-03
$f_{5\rightarrow 0c\_PI5P}$	1.31E-03	6.99E-04	-2.66E-04	-1.38E-01	9.10E-05	-1.15E-02	-1.23E-03	1.16E-02	6.53E-03	-8.17E-03	-9.99E-03	-1.13E-02	-7.77E-03	1.06E-02	2.06E-02
$f_{5\rightarrow 0c\_PI3P}$	-2.43E-07	-2.33E-07	1.84E-06	8.31E-05	-1.20E-07	1.15E-06	1.90E-06	-1.23E-06	-7.25E-07	8.17E-07	1.10E-06	1.10E-06	-7.12E-06	-9.61E-06	-1.57E-05
$f_{5\rightarrow 0c\_PI4P}$	-1.61E-04	-8.62E-05	3.47E-05	1.71E-02	-1.13E-05	1.42E-03	1.53E-04	-1.42E-03	-8.04E-04	1.01E-03	1.23E-03	1.38E-03	9.48E-04	-1.31E-03	-2.55E-03

$f_{5\to0c\_PI35P2}$	2.83E-07	4.80E-08	1.73E-06	2.75E-05	-8.32E-08	-3.48E-06	1.41E-06	3.41E-06	1.90E-06	-2.46E-06	-2.91E-06	-3.42E-06	-1.02E-05	-5.35E-06	-7.44E-06
$\gamma_{3\to35}$	-6.61E-04	-8.83E-01	1.31E-04	9.95E-02	1.09E-01	8.31E-03	5.98E-04	-8.35E-03	-4.72E-03	5.89E-03	7.20E-03	8.12E-03	5.59E-03	-7.67E-03	-1.49E-02
$f_{3\to35\_PI3P}$	-2.95E-03	-3.94E+00	5.68E-04	4.45E-01	4.85E-01	3.72E-02	2.65E-03	-3.73E-02	-2.11E-02	2.63E-02	3.22E-02	3.63E-02	2.50E-02	-3.43E-02	-6.64E-02
$f_{3\to35\_PI}$	3.90E-03	5.19E+00	-7.89E-04	-5.85E-01	-6.39E-01	-4.90E-02	-3.54E-03	4.92E-02	2.78E-02	-3.47E-02	-4.24E-02	-4.78E-02	-3.30E-02	4.51E-02	8.74E-02
$\gamma_{35\to3a}$	3.33E-06	3.71E-03	1.13E-06	-4.18E-04	-4.56E-04	-4.07E-05	-1.35E-06	4.08E-05	2.31E-05	-2.89E-05	-3.52E-05	-3.98E-05	-3.53E-05	2.90E-05	5.91E-05
$f_{35\to3a\_PI3P}$	5.43E-07	-7.20E-06	1.68E-06	1.90E-07	8.45E-07	-5.75E-06	1.16E-06	5.70E-06	3.19E-06	-4.08E-06	-4.89E-06	-5.64E-06	-1.18E-05	-3.26E-06	-3.38E-06
$f_{35\to3a\_PI4P}$	-5.83E-07	-1.51E-03	1.90E-06	1.70E-04	1.86E-04	8.42E-06	2.18E-06	-8.53E-06	-4.85E-06	5.97E-06	7.39E-06	8.20E-06	-2.23E-06	-1.63E-05	-2.87E-05
$f_{35\to3a\_PI5P}$	5.43E-07	-7.25E-06	1.68E-06	1.95E-07	8.51E-07	-5.75E-06	1.16E-06	5.70E-06	3.19E-06	-4.08E-06	-4.89E-06	-5.64E-06	-1.18E-05	-3.26E-06	-3.38E-06
$f_{35\to3a\_PI35P2}$	9.18E-06	1.15E-02	-1.93E-08	-1.30E-03	-1.42E-03	-1.14E-04	-6.63E-06	1.15E-04	6.48E-05	-8.11E-05	-9.90E-05	-1.12E-04	-8.49E-05	9.68E-05	1.91E-04
$f_{35\to3a\_PI45P2\_PI4P}$	-5.69E-07	-1.49E-03	1.90E-06	1.67E-04	1.83E-04	8.24E-06	2.17E-06	-8.35E-06	-4.75E-06	5.84E-06	7.23E-06	8.02E-06	-2.35E-06	-1.61E-05	-2.84E-05
$f_{35\to3a\_PI45P2\_PI5P}$	-5.47E-06	-8.03E-03	2.86E-06	9.05E-04	9.89E-04	6.99E-05	6.59E-06	-7.03E-05	-3.97E-05	4.96E-05	6.07E-05	6.82E-05	3.92E-05	-7.29E-05	-1.38E-04
$f_{35\to3a\_PI345P3}$	5.48E-07	-5.56E-07	1.68E-06	-5.59E-07	2.72E-08	-5.81E-06	1.16E-06	5.76E-06	3.22E-06	-4.12E-06	-4.94E-06	-5.70E-06	-1.18E-05	-3.20E-06	-3.27E-06
$f_{35\to3a\_PI45P2\_PI}$	2.69E-07	-3.73E-04	1.73E-06	4.14E-05	4.59E-05	-2.30E-06	1.41E-06	2.23E-06	1.23E-06	-1.63E-06	-1.90E-06	-2.27E-06	-9.45E-06	-6.44E-06	-9.54E-06
$\gamma_{35\to3c}$	5.22E-06	6.22E-03	7.61E-07	-7.02E-04	-7.66E-04	-6.45E-05	-3.06E-06	6.47E-05	3.65E-05	-4.57E-05	-5.58E-05	-6.30E-05	-5.13E-05	5.08E-05	1.02E-04
$f_{35\to3c\_PI35P2}$	1.50E-05	1.93E-02	-1.17E-06	-2.18E-03	-2.38E-03	-1.88E-04	-1.19E-05	1.89E-04	1.07E-04	-1.33E-04	-1.63E-04	-1.84E-04	-1.35E-04	1.65E-04	3.22E-04
$f_{35\to3c\_PI3P}$	5.36E-07	-1.74E-05	1.68E-06	1.34E-06	2.10E-06	-5.65E-06	1.17E-06	5.60E-06	3.13E-06	-4.01E-06	-4.80E-06	-5.55E-06	-1.17E-05	-3.35E-06	-3.55E-06
$f_{35\to3c\_PI4P}$	-2.15E-06	-3.60E-03	2.21E-06	4.05E-04	4.42E-04	2.81E-05	3.59E-06	-2.83E-05	-1.60E-05	1.99E-05	2.44E-05	2.74E-05	1.10E-05	-3.44E-05	-6.38E-05
$f_{35\to3c\_PI5P}$	5.36E-07	-1.75E-05	1.68E-06	1.35E-06	2.12E-06	-5.65E-06	1.17E-06	5.60E-06	3.13E-06	-4.01E-06	-4.80E-06	-5.54E-06	-1.17E-05	-3.35E-06	-3.55E-06
$\gamma_{35\to3d}$	9.38E-07	5.20E-04	1.60E-06	-5.92E-05	-6.40E-05	-1.07E-05	8.05E-07	1.07E-05	6.01E-06	-7.60E-06	-9.19E-06	-1.05E-05	-1.51E-05	1.32E-06	5.49E-06
$f_{35\to3d\_PI35P2}$	1.76E-06	1.61E-03	1.44E-06	-1.83E-04	-1.99E-04	-2.10E-05	6.42E-08	2.10E-05	1.19E-05	-1.49E-05	-1.81E-05	-2.06E-05	-2.21E-05	1.08E-05	2.39E-05
$\gamma_{35\to3e}$	5.99E-07	6.69E-05	1.67E-06	-8.16E-06	-8.27E-06	-6.45E-06	1.11E-06	6.40E-06	3.59E-06	-4.57E-06	-5.49E-06	-6.32E-06	-1.22E-05	-2.62E-06	-2.13E-06
$f_{35\to3e\_PI35P2}$	7.04E-07	2.07E-04	1.65E-06	-2.40E-05	-2.56E-05	-7.77E-06	1.02E-06	7.73E-06	4.34E-06	-5.51E-06	-6.64E-06	-7.61E-06	-1.31E-05	-1.40E-06	2.31E-07
$f_{35\to3e\_PI45P2}$	5.20E-07	-3.78E-05	1.68E-06	3.64E-06	4.61E-06	-5.46E-06	1.18E-06	5.41E-06	3.03E-06	-3.87E-06	-4.64E-06	-5.36E-06	-1.16E-05	-3.52E-06	-3.89E-06
$f_{35\to3e\_PI345P3}$	5.49E-07	1.71E-07	1.68E-06	-6.41E-07	-6.23E-08	-5.82E-06	1.16E-06	5.77E-06	3.23E-06	-4.13E-06	-4.95E-06	-5.71E-06	-1.18E-05	-3.19E-06	-3.26E-06
$\gamma_{4\to45}$	-2.66E-02	9.94E-03	-4.12E-01	6.48E-02	2.23E-02	4.31E-01	-3.64E-01	-4.30E-01	-2.43E-01	3.05E-01	3.22E-01	4.26E-01	2.55E-01	-3.99E-01	-7.69E-01
$f_{4\to45\_PI4P}$	-1.14E-02	4.24E-03	-1.76E-01	2.78E-02	9.53E-03	1.85E-01	-1.56E-01	-1.85E-01	-1.05E-01	1.31E-01	1.38E-01	1.83E-01	1.09E-01	-1.70E-01	-3.30E-01
$f_{4\to45\_PA}$	5.49E-07	1.90E-07	1.68E-06	-6.43E-07	-6.47E-08	-5.82E-06	1.16E-06	5.77E-06	3.23E-06	-4.13E-06	-4.95E-06	-5.71E-06	-1.18E-05	-3.19E-06	-3.26E-06
$f_{4\to45\_PI45P2}$	1.22E-02	-4.51E-03	1.88E-01	-2.97E-02	-1.02E-02	-1.97E-01	1.67E-01	1.99E-01	1.12E-01	-1.40E-01	-1.47E-01	-1.95E-01	-1.17E-01	1.81E-01	3.52E-01
$\gamma_{45\to4a}$	9.30E-03	-3.45E-03	1.44E-01	-2.27E-02	-7.76E-03	-1.51E-01	1.27E-01	1.52E-01	8.56E-02	-1.07E-01	-1.13E-01	-1.49E-01	-8.93E-02	1.38E-01	2.69E-01
$f_{45\to4a\_PI3P}$	-1.80E-05	7.09E-06	-2.86E-04	4.46E-05	1.54E-05	2.95E-04	-2.53E-04	-2.96E-04	-1.67E-04	2.09E-04	2.20E-04	2.92E-04	1.66E-04	-2.80E-04	-5.41E-04
$f_{45\to4a\_PI4P}$	-3.79E-03	1.41E-03	-5.88E-02	9.25E-03	3.17E-03	6.15E-02	-5.20E-02	-6.17E-02	-3.49E-02	4.36E-02	4.60E-02	6.08E-02	3.64E-02	-5.67E-02	-1.10E-01
$f_{45\to4a\_PI5P}$	-1.81E-05	7.13E-06	-2.87E-04	4.49E-05	1.55E-05	2.97E-04	-2.55E-04	-2.98E-04	-1.68E-04	2.10E-04	2.21E-04	2.94E-04	1.68E-04	-2.82E-04	-5.44E-04
$f_{45\to4a\_PI35P2}$	-5.70E-06	2.51E-06	-9.50E-05	1.46E-05	5.15E-06	9.54E-05	-8.44E-05	-9.59E-05	-5.42E-05	6.76E-05	7.07E-05	9.44E-05	4.81E-05	-9.64E-05	-1.84E-04
$f_{45\to4a\_PI45P2\_PI4P}$	8.47E-02	-3.08E-02	1.31E+00	-2.06E-01	-7.01E-02	-1.37E+00	1.16E+00	1.40E+00	7.90E-01	-9.76E-01	-1.02E+00	-1.36E+00	-8.13E-01	1.24E+00	2.44E+00
$f_{45\to4a\_PI45P2\_PI5P}$	-2.02E-02	7.55E-03	-3.13E-01	4.93E-02	1.69E-02	3.28E-01	-2.77E-01	-3.28E-01	-1.85E-01	2.32E-01	2.45E-01	3.24E-01	1.94E-01	-3.03E-01	-5.85E-01
$f_{45\to4a\_PI345P3}$	-1.33E-06	8.87E-07	-2.73E-05	3.92E-06	1.50E-06	2.46E-05	-2.45E-05	-2.47E-05	-1.40E-05	1.74E-05	1.78E-05	2.43E-05	6.18E-06	-3.12E-05	-5.75E-05
$f_{45\to4a\_PI45P2\_PI}$	-9.37E-04	3.49E-04	-1.45E-02	2.28E-03	7.84E-04	1.52E-02	-1.28E-02	-1.53E-02	-8.62E-03	1.08E-02	1.14E-02	1.50E-02	8.99E-03	-1.40E-02	-2.71E-02
$\gamma_{45\to4c}$	1.89E-03	-7.02E-04	2.93E-02	-4.61E-03	-1.58E-03	-3.07E-02	2.59E-02	3.08E-02	1.74E-02	-2.17E-02	-2.29E-02	-3.03E-02	-1.82E-02	2.82E-02	5.47E-02
$f_{45\to4c\_PI45P2}$	1.70E-02	-6.29E-03	2.63E-01	-4.14E-02	-1.42E-02	-2.75E-01	2.33E-01	2.78E-01	1.57E-01	-1.95E-01	-2.06E-01	-2.72E-01	-1.63E-01	2.53E-01	4.92E-01
$f_{45\to4c\_PI345P3}$	9.02E-09	3.91E-07	-6.68E-06	6.73E-07	3.86E-07	2.93E-06	-6.24E-06	-3.02E-06	-1.74E-06	2.08E-06	1.59E-06	2.95E-06	-6.63E-06	-1.13E-05	-1.89E-05
$\gamma_{45\to4d}$	1.68E-04	-6.20E-05	2.59E-03	-4.09E-04	-1.40E-04	-2.72E-03	2.29E-03	2.73E-03	1.54E-03	-1.93E-03	-2.03E-03	-2.69E-03	-1.62E-03	2.50E-03	4.84E-03
$f_{45\to4d\_PI45P2}$	1.51E-03	-5.60E-04	2.33E-02	-3.68E-03	-1.26E-03	-2.44E-02	2.07E-02	2.45E-02	1.39E-02	-1.73E-02	-1.83E-02	-2.42E-02	-1.45E-02	2.25E-02	4.36E-02
$f_{45\to4d\_PI35P2}$	3.90E-07	2.49E-07	-7.85E-07	-2.55E-07	6.83E-08	-3.24E-06	-1.02E-06	3.18E-06	1.76E-06	-2.30E-06	-3.02E-06	-3.16E-06	-1.03E-05	-5.57E-06	-7.86E-06
$f_{45\to4d\_PI345P3}$	5.01E-07	2.08E-07	9.39E-07	-5.27E-07	-2.47E-08	-5.05E-06	5.02E-07	4.99E-06	2.79E-06	-3.58E-06	-4.37E-06	-4.94E-06	-1.14E-05	-3.91E-06	-4.64E-06
$\gamma_{5\to45}$	-5.72E-04	-3.14E-04	1.09E-04	-1.03E+00	-5.05E-05	8.40E-03	5.03E-04	-8.44E-03	-4.77E-03	5.96E-03	7.28E-03	8.21E-03	5.65E-03	-7.75E-03	-1.50E-02
$f_{5\to45\_PI5P}$	-2.26E-03	-1.24E-03	4.15E-04	-4.05E+00	-2.00E-04	3.32E-02	1.97E-03	-3.33E-02	-1.88E-02	2.35E-02	2.87E-02	3.24E-02	2.23E-02	-3.06E-02	-5.93E-02
$\gamma_{45\to5a}$	7.08E-05	3.87E-05	-1.17E-05	1.26E-01	6.11E-06	-1.04E-03	-6.06E-05	1.04E-03	5.89E-04	-7.35E-04	-8.98E-04	-1.01E-03	-7.06E-04	9.47E-04	1.84E-03
$f_{45\to5a\_PI3P}$	4.09E-07	1.13E-07	1.71E-06	-2.52E-04	-7.70E-08	-3.76E-06	1.28E-06	3.70E-06	2.06E-06	-2.67E-06	-3.16E-06	-3.70E-06	-1.04E-05	-5.09E-06	-6.93E-06
$f_{45\to5a\_PI4P}$	-2.81E-05	-1.55E-05	7.11E-06	-5.13E-02	-2.58E-06	4.15E-04	2.63E-05	-4.16E-04	-2.35E-04	2.94E-04	3.59E-04	4.05E-04	2.71E-04	-3.90E-04	-7.54E-04
$f_{45\to5a\_PI5P}$	4.08E-07	1.13E-07	1.71E-06	-2.53E-04	-7.71E-08	-3.75E-06	1.28E-06	3.69E-06	2.05E-06	-2.66E-06	-3.15E-06	-3.69E-06	-1.04E-05	-5.10E-06	-6.95E-06
$f_{45\to5a\_PI35P2}$	5.02E-07	1.64E-07	1.69E-06	-8.51E-05	-6.88E-08	-5.13E-06	1.20E-06	5.07E-06	2.84E-06	-3.64E-06	-4.35E-06	-5.03E-06	-1.14E-05	-3.83E-06	-4.49E-06
$f_{45\to5a\_PI45P2\_PI4P}$	-2.77E-05	-1.53E-05	7.04E-06	-5.07E-02	-2.55E-06	4.09E-04	2.60E-05	-4.11E-04	-2.32E-04	2.90E-04	3.54E-04	4.00E-04	2.68E-04	-3.85E-04	-7.44E-04
$f_{45\to5a\_PI45P2\_PI5P}$	5.10E-04	2.80E-04	-9.57E-05	9.14E-01	4.47E-05	-7.49E-03	-4.48E-04	7.53E-03	4.25E-03	-5.31E-03	-6.49E-03	-7.32E-03	-5.05E-03	6.89E-03	1.34E-02
$f_{45\to5a\_PI345P3}$	5.35E-07	1.83E-07	1.68E-06	-2.60E-05	-6.59E-08	-5.61E-06	1.17E-06	5.56E-06	3.11E-06	-3.98E-06	-4.77E-06	-5.50E-06	-1.17E-05	-3.39E-06	-3.63E-06
$f_{45\to5a\_PI45P2\_PI}$	-6.53E-06	-3.69E-06	3.02E-06	-1.27E-02	-6.87E-07	9.81E-05	7.38E-06	-9.86E-05	-5.58E-05	6.95E-05	8.50E-05	9.58E-05	5.81E-05	-9.89E-05	-1.89E-04
$\gamma_{45\to5c}$	8.69E-06	4.65E-06	1.36E-07	1.46E-02	6.51E-07	-1.25E-04	-5.99E-06	1.26E-04	7.11E-05	-8.88E-05	-1.08E-04	-1.22E-04	-9.23E-05	1.07E-04	2.10E-04
$f_{45\to5c\_PI45P2}$	5.06E-05	2.76E-05	-7.81E-06	8.97E-02	4.33E-06	-7.40E-04	-4.28E-05	7.43E-04	4.20E-04	-5.25E-04	-6.41E-04	-7.23E-04	-5.06E-04	6.73E-04	1.31E-03
$\gamma_{45\to345}$	-9.40E-05	2.56E-05	-1.07E-04	-3.17E-04	8.80E-05	8.17E-04	2.81E-03	2.67E+00	1.54E+00	5.68E-04	6.93E-04	8.00E-04	5.32E-04	-6.50E-04	-1.49E-03
$f_{45\to345\_PI45P2}$	-2.67E-04	7.29E-05	-3.06E-04	-8.98E-04	2.50E-04	2.32E-03	8.07E-03	7.76E+00	4.43E+00	1.61E-03	1.97E-03	2.27E-03	1.52E-03	-1.83E-03	-4.21E-03
$\gamma_{345\to45}$	9.37E-05	-2.46E-05	1.08E-04	3.11E-04	-8.65E-05	-8.19E-04	-2.72E-03	-2.56E+00	-1.49E+00	-5.70E-04	-6.95E-04	-8.02E-04	-5.49E-04	6.35E-04	1.47E-03
$f_{345\to45\_PI345P3}$	1.11E-05	-2.64E-06	1.38E-05	3.47E-05	-9.89E-06	-9.81E-05	-3.10E-04	-2.94E-01	-1.71E-01	-6.83E-05	-8.32E-05	-9.60E-05	-7.28E-05	6.94E-05	1.64E-04
$f_{345\to45\_PI34P2}$	5.11E-07	2.00E-07	1.64E-06	-7.71E-07	-2.92E-08	-5.49E-06	2.28E-06	1.07E-03	6.19E-04	-3.89E-06	-4.66E-06	-5.38E-06	-1.16E-05	-3.46E-06	-3.86E-06
$\gamma_{35\to5}$	-1.51E-05	-1.03E-02	-4.59E-05	1.03E-02	-9.79E-01	8.24E-04	-3.69E-05	-8.29E-04	-4.69E-04	5.84E-04	7.07E-04	8.06E-04	5.42E-04	-7.68E-04	-1.48E-03
$f_{35\to5\_PI35P2}$	-4.64E-05	-3.11E-02	-1.44E-04	3.10E-02	-2.95E+00	2.49E-03	-1.16E-04	-2.51E-03	-1.42E-03	1.77E-03	2.14E-03	2.44E-03	1.66E-03	-2.31E-03	-4.47E-03
$f_{35\to5\_PI3P}$	5.97E-07	3.17E-05	1.82E-06	-3.20E-05	2.99E-03	-8.35E-06	1.27E-06	8.31E-06	4.67E-06	-5.92E-06	-7.12E-06	-8.18E-06	-1.35E-05	-8.61E-07	1.26E-06
$\gamma_{34\to3}$	-3.85E-06	3.53E-03	1.38E-05	3.22E-03	3.53E-03	2.70E-04	-2.05E-01	-3.30E-04	-1.87E-04	1.91E-04	2.35E-04	2.63E-04	1.74E-04	-2.57E-04	-4.95E-04
$f_{34\to3\_PI34P2}$	-1.59E-05	1.32E-02	4.68E-05	1.20E-02	1.32E-02	1.02E-03	-7.66E-01	-1.25E-03	-7.08E-04	7.25E-04	8.90E-04	9.99E-04	6.83E-04	-9.50E-04	-1.84E-03
$\gamma_{345\to34a}$	6.03E-07	2.41E-06	1.68E-06	1.36E-06	2.12E-06	-6.94E-06	4.98E-04	-8.03E-04	-4.65E-04	-4.92E-06	-5.92E-06	-6.80E-06	-1.26E-05	-2.20E-06	-1.25E-06
$f_{345\to34a\_PI3P}$	5.49E-07	1.86E-07	1.68E-06	-6.47E-07	-6.90E-08	-5.82E-06	1.66E-07	7.38E-06	4.16E-06	-4.12E-06	-4.94E-06	-5.71E-06	-1.18E-05	-3.20E-06	-3.26E-06
$f_{345\to34a\_PI4P}$	5.27E-07	-7.13E-07	1.68E-06	-1.46E-06	-9.56E-07	-5.36E-06	-2.01E-04	3.35E-04	1.94E-04	-3.80E-06	-4.55E-06	-5.26E-06	-1.15E-05	-3.60E-06	-4.07E-06
$f_{345\to34a\_PI5P}$	5.49E-07	1.86E-07	1.68E-06	-6.47E-07	-6.90E-08	-5.82E-06	1.60E-07	7.39E-06	4.17E-06	-4.12E-06	-4.94E-06	-5.71E-06	-1.18E-05	-3.20E-06	-3.26E-06
$f_{345\to34a\_PI35P2}$	5.49E-07	1.89E-07	1.68E-06	-6.44E-07	-6.61E-08	-5.82E-06	8.23E-07	6.31E-06	3.54E-06	-4.13E-06	-4.95E-06	-5.71E-06	-1.18E-05	-3.19E-06	-3.26E-06
$f_{345\to34a\_PI45P2\_PI4P}$	5.27E-07	-7.02E-07	1.68E-06	-1.45E-06	-9.44E-07	-5.37E-06	-1.99E-04	3.31E-04	1.91E-04	-3.81E-06	-4.56E-06	-5.27E-06	-1.15E-05	-3.60E-06	-4.06E-06
$f_{345\to34a\_PI45P2\_PI5P}$	4.32E-07	-4.61E-06	1.68E-06	-4.99E-06	-4.80E-06	-3.39E-06	-1.08E-03	1.76E-03	1.02E-03	-2.41E-06	-2.84E-06	-3.33E-06	-1.02E-05	-5.36E-06	-7.61E-06
$f_{345\to34a\_PI345P3}$	5.55E-07	4.58E-07	1.68E-06	-4.01E-07	1.99E-07	-5.96E-06	6.10E-05	-9.17E-05	-5.32E-05	-4.22E-06	-5.06E-06	-5.84E-06	-1.19E-05	-3.07E-06	-3.01E-06
$f_{345\to34a\_PI45P2\_PI}$	5.43E-07	-3.30E-08	1.68E-06	-8.45E-07	-2.85E-07	-5.71E-06	-4.89E-05	8.72E-05	5.04E-05	-4.05E-06	-4.85E-06	-5.60E-06	-1.17E-05	-3.29E-06	-3.46E-06
$\gamma_{345\to34c}$	5.60E-07	6.41E-07	1.68E-06	-2.35E-07	3.80E-07	-6.05E-06	1.02E-04	-1.59E-04	-9.19E-05	-4.29E-06	-5.14E-06	-5.93E-06	-1.20E-05	-2.99E-06	-2.85E-06
$f_{345\to34c\_PI345P3}$	5.50E-07	2.45E-07	1.68E-06	-5.94E-07	-1.11E-08	-5.85E-06	1.33E-05	-1.40E-05	-8.23E-06	-4.15E-06	-4.97E-06	-5.73E-06	-1.18E-05	-3.17E-06	-3.21E-06
$f_{345\to34c\_PI45P2}$	5.43E-07	-6.64E-08	1.68E-06	-8.75E-07	-3.18E-07	-5.69E-06	-5.64E-05	9.94E-05	5.74E-05	-4.03E-06	-4.83E-06	-5.58E-06	-1.17E-05	-3.31E-06	-3.49E-06
$\gamma_{345\to34d}$	5.50E-07	2.30E-07	1.68E-06	-6.07E-07	-2.53E-08	-5.84E-06	1.01E-05	-8.78E-06	-5.19E-06	-4.14E-06	-4.96E-06	-5.73E-06	-1.18E-05	-3.18E-06	-3.22E-06
$f_{345\to34d\_PI345P3}$	5.49E-07	1.95E-07	1.68E-06	-6.39E-07	-5.99E-08	-5.82E-06	2.23E-06	4.01E-06	2.21E-06	-4.13E-06	-4.95E-06	-5.71E-06	-1.18E-05	-3.19E-06	-3.25E-06
$f_{345\to34d\_PI35P2}$	5.49E-07	1.90E-07	1.68E-06	-6.43E-07	-6.47E-08	-5.82E-06	1.15E-06	5.78E-06	3.24E-06	-4.13E-06	-4.95E-06	-5.71E-06	-1.18E-05	-3.19E-06	-3.26E-06
$f_{345\to34d\_PI45P2}$	5.48E-07	1.68E-07	1.68E-06	-6.64E-07	-8.71E-08	-5.81E-06	-3.93E-06	1.41E-05	8.02E-06	-4.12E-06	-4.94E-06	-5.70E-06	-1.18E-05	-3.20E-06	-3.28E-06
$\gamma_{345\to34e}$	5.51E-07	2.74E-07	1.68E-06	-5.67E-07	1.81E-08	-5.86E-06	1.99E-05	-2.48E-05	-1.45E-05	-4.16E-06	-4.98E-06	-5.75E-06	-1.18E-05	-3.16E-06	-3.18E-06
$f_{345\to34e\_PI345P3}$	5.49E-07	2.00E-07	1.68E-06	-6.34E-07	-5.47E-08	-5.83E-06	3.42E-06	2.08E-06	1.09E-06	-4.13E-06	-4.95E-06	-5.71E-06	-1.18E-05	-3.19E-06	-3.25E-06
$\gamma_{I\to}$	-9.45E-01	-5.32E-01	-7.45E-01	-2.64E-01	-1.15E-01	-7.52E-01	-6.87E-01	7.25E-01	4.08E-01	-5.34E-01	-7.41E-01	-7.25E-01	-5.68E-01	6.85E-01	1.34E+00
$\gamma_{0\to45}$	-1.01E-01	-5.42E-02	2.05E-02	8.48E-02	-7.19E-03	9.25E-01	9.49E-02	-9.18E-01	-5.19E-01	6.54E-01	8.02E-01	9.03E-01	6.23E-01	-8.61E-01	-1.65E+00
$f_{0\to45\_PI}$	-3.73E-01	-2.00E-01	7.45E-02	3.11E-01	-2.67E-02	3.41E+00	3.50E-01	-3.28E+00	-1.86E+00	2.39E+00	2.95E+00	3.33E+00	2.29E+00	-3.23E+00	-6.03E+00
$f_{0\to45\_PA}$	5.49E-07	1.90E-07	1.68E-06	-6.43E-07	-6.47E-08	-5.82E-06	1.16E-06	5.77E-06	3.23E-06	-4.13E-06	-4.95E-06	-5.71E-06	-1.18E-05	-3.19E-06	-3.26E-06

$f_{0\rightarrow 45\_PI45P2}$	4.63E-02	2.48E-02	-9.46E-03	-3.89E-02	3.27E-03	-4.23E-01	-4.34E-02	4.27E-01	2.41E-01	-3.00E-01	-3.67E-01	-4.13E-01	-2.85E-01	3.88E-01	7.55E-01
$\gamma_{4\rightarrow 34b}$	5.49E-07	1.90E-07	1.68E-06	-6.43E-07	-6.47E-08	-5.82E-06	1.16E-06	5.77E-06	3.23E-06	-4.13E-06	-4.95E-06	-5.71E-06	-1.18E-05	-3.19E-06	-3.26E-06
$f_{4\rightarrow 34b\_PI4P}$	5.49E-07	1.90E-07	1.68E-06	-6.43E-07	-6.47E-08	-5.82E-06	1.16E-06	5.77E-06	3.23E-06	-4.13E-06	-4.95E-06	-5.71E-06	-1.18E-05	-3.19E-06	-3.26E-06
$f_{4\rightarrow 34b\_PI}$	-6.57E-05	4.51E-03	-1.86E-03	4.12E-03	4.55E-03	1.35E-04	9.98E-01	1.50E-04	8.87E-05	9.56E-05	-1.12E-04	1.55E-04	-7.31E-05	-1.33E-04	-2.54E-04
$\gamma_{34\rightarrow 4}$	-5.98E-04	4.08E-02	-1.69E-02	3.72E-02	4.11E-02	1.27E-03	9.03E+00	1.21E-03	7.20E-04	8.97E-04	-9.73E-04	1.45E-03	-5.66E-04	-1.17E-03	-2.27E-03
$f_{34\rightarrow 4\_PI34P2}$	6.52E-04	-4.44E-02	1.84E-02	-4.05E-02	-4.48E-02	-1.39E-03	-9.83E+00	-1.58E-03	-9.32E-04	-9.86E-04	1.05E-03	-1.59E-03	5.92E-04	1.27E-03	2.47E-03
$\gamma_{i\rightarrow}$	1.21E-06	-4.48E-05	2.03E-05	-4.17E-05	-4.54E-05	-7.22E-06	-9.94E-03	4.31E-06	2.37E-06	-5.12E-06	-3.88E-06	-7.31E-06	-1.12E-05	-1.90E-06	-7.54E-07
$\gamma_{0\rightarrow 45}$	3.03E-06	-1.69E-04	7.17E-05	-1.55E-04	-1.71E-04	-1.11E-05	-3.74E-02	2.94E-07	-8.82E-09	-7.87E-06	-9.31E-07	-1.17E-05	-9.52E-06	1.66E-06	6.16E-06
$f_{34\rightarrow 4\_PI345P3}$	5.45E-07	4.32E-07	1.58E-06	-4.23E-07	1.78E-07	-5.81E-06	5.45E-05	5.77E-06	3.23E-06	-4.12E-06	-4.95E-06	-5.70E-06	-1.18E-05	-3.20E-06	-3.27E-06
$\gamma_{45\rightarrow 0}$	4.84E-06	2.49E-06	8.05E-07	-4.24E-06	2.39E-07	-4.50E-05	-2.87E-06	4.51E-05	2.55E-05	-3.19E-05	-3.89E-05	-4.40E-05	-3.82E-05	3.29E-05	6.67E-05
$f_{45\rightarrow 0\_PI3P}$	2.05E-05	1.09E-05	-2.39E-06	-1.74E-05	1.35E-06	-1.88E-04	-1.76E-05	1.89E-04	1.07E-04	-1.33E-04	-1.63E-04	-1.84E-04	-1.35E-04	1.65E-04	3.22E-04
$f_{45\rightarrow 0\_PI4P}$	-1.20E-06	-7.46E-07	2.04E-06	8.25E-07	-1.88E-07	1.02E-05	2.80E-06	-1.03E-05	-5.83E-06	7.20E-06	8.91E-06	9.90E-06	-1.05E-06	-1.79E-05	-3.18E-05
$f_{45\rightarrow 0\_PI5P}$	5.40E-07	1.86E-07	1.68E-06	-6.36E-07	-6.53E-08	-5.74E-06	1.16E-06	5.69E-06	3.18E-06	-4.07E-06	-4.88E-06	-5.63E-06	-1.18E-05	-3.27E-06	-3.40E-06
$f_{45\rightarrow 0\_PI35P2}$	5.46E-07	1.89E-07	1.68E-06	-6.41E-07	-6.49E-08	-5.79E-06	1.16E-06	5.74E-06	3.21E-06	-4.11E-06	-4.92E-06	-5.68E-06	-1.18E-05	-3.22E-06	-3.30E-06
$f_{45\rightarrow 0\_PI45P2\_PI4P}$	-1.18E-06	-7.34E-07	2.03E-06	8.06E-07	-1.87E-07	9.95E-06	2.78E-06	-1.01E-05	-5.72E-06	7.06E-06	8.73E-06	9.70E-06	-1.18E-06	-1.77E-05	-3.14E-05
$f_{45\rightarrow 0\_PI45P2\_PI5P}$	-8.76E-06	-4.79E-06	3.57E-06	7.16E-06	-7.22E-07	7.91E-05	9.88E-06	-7.95E-05	-4.50E-05	5.61E-05	6.87E-05	7.73E-05	4.55E-05	-8.14E-05	-1.55E-04
$f_{45\rightarrow 0\_PI345P3}$	5.48E-07	1.90E-07	1.68E-06	-6.42E-07	-6.47E-08	-5.81E-06	1.16E-06	5.76E-06	3.22E-06	-4.12E-06	-4.94E-06	-5.70E-06	-1.18E-05	-3.20E-06	-3.27E-06
$f_{45\rightarrow 0\_PI45P2\_PI}$	1.16E-07	-4.12E-08	1.77E-06	-2.80E-07	-9.52E-08	-1.87E-06	1.56E-06	1.80E-06	9.89E-07	-1.33E-06	-1.52E-06	-1.85E-06	-9.16E-06	-6.83E-06	-1.03E-05
$\gamma_{PI3KIc\rightarrow PI3KIa}$	-7.76E-05	2.13E-05	-8.79E-05	-2.63E-04	7.28E-05	6.75E-04	2.32E-03	2.21E+00	2.11E+00	4.64E-04	5.72E-04	6.61E-04	4.38E-04	-4.78E-04	-1.25E-03
$f_{PI3KIc\rightarrow PI3KIa\_pi\_3KI\_c}$	-5.46E-04	1.52E-04	-6.30E-04	-1.84E-03	5.14E-04	4.72E-03	1.69E-02	1.66E+01	1.58E+01	3.25E-03	4.00E-03	4.62E-03	3.11E-03	-3.32E-03	-8.64E-03
$f_{PI3KIc\rightarrow PI3KIa\_PI345P3}$	-6.14E-06	1.99E-06	-5.98E-06	-2.31E-05	6.17E-06	5.26E-05	1.98E-04	1.87E-01	1.79E-01	3.59E-05	4.45E-05	5.14E-05	2.67E-05	-4.37E-05	-1.10E-04
$\gamma_{PI3KIa\rightarrow PI3KIc}$	7.89E-05	-2.08E-05	9.13E-05	2.62E-04	-7.29E-05	-6.90E-04	-2.29E-03	-2.16E+00	-2.07E+00	-4.74E-04	-5.85E-04	-6.76E-04	-4.64E-04	4.73E-04	1.25E-03
$f_{PI3KIa\rightarrow PI3KIc\_pi\_3KI\_a}$	5.49E-07	1.90E-07	1.68E-06	-6.43E-07	-6.47E-08	-5.82E-06	1.16E-06	5.77E-06	3.23E-06	-4.13E-06	-4.95E-06	-5.71E-06	-1.18E-05	-3.19E-06	-3.26E-06
$\gamma_{PTENc\rightarrow PTENa}$	6.57E-05	-4.89E-05	8.89E-05	1.87E-04	-9.22E-05	-5.72E-04	-8.93E-03	-1.79E+00	-1.02E+00	7.07E-01	-4.83E-04	-5.60E-04	-3.85E-04	4.34E-04	1.02E-03
$f_{PTENc\rightarrow PTENa\_PTEN\_c}$	2.33E-04	-1.75E-04	3.12E-04	6.64E-04	-3.29E-04	-2.03E-03	-3.20E-02	-6.22E+00	-3.60E+00	2.54E+00	-1.71E-03	-1.98E-03	-1.34E-03	1.56E-03	3.66E-03
$f_{PTENc\rightarrow PTENa\_PI45P2}$	5.95E-04	-4.54E-04	7.96E-04	1.69E-03	-8.46E-04	-5.21E-03	-8.30E-02	-1.51E+01	-8.92E+00	6.64E+00	-4.40E-03	-5.10E-03	-3.44E-03	4.01E-03	9.42E-03
$\gamma_{PTENa\rightarrow PTENc}$	-6.48E-05	4.93E-05	-8.58E-05	-1.89E-04	9.22E-05	5.61E-04	8.92E-03	1.83E+00	1.04E+00	-7.04E-01	4.74E-04	5.49E-04	3.61E-04	-4.42E-04	-1.03E-03
$f_{PTENa\rightarrow PTENc\_PTEN\_a}$	-1.75E-04	1.31E-04	-2.33E-04	-5.07E-04	2.47E-04	1.51E-03	2.38E-02	4.98E+00	2.82E+00	-1.87E+00	1.28E-03	1.48E-03	9.87E-04	-1.18E-03	-2.75E-03
$f_{PTENa\rightarrow PTENc\_PI345P3}$	5.49E-07	1.90E-07	1.68E-06	-6.43E-07	-6.47E-08	-5.82E-06	1.16E-06	5.77E-06	3.23E-06	-4.13E-06	-4.95E-06	-5.71E-06	-1.18E-05	-3.19E-06	-3.26E-06
$\gamma_{45\rightarrow DAG}$	-1.74E-02	-1.94E-03	-1.70E-01	-4.38E-02	5.30E-03	-4.65E-01	-1.39E-01	4.70E-01	2.65E-01	-3.30E-01	4.60E-01	5.43E-01	2.75E-01	4.26E-01	8.29E-01
$f_{45\rightarrow DAG\_PI45P2}$	-1.56E-01	-1.68E-02	-1.53E+00	-3.98E-01	4.80E-02	-4.19E+00	-1.26E+00	4.45E+00	2.49E+00	-3.01E+00	4.14E+00	4.89E+00	2.47E+00	3.62E+00	7.34E+00
$f_{45\rightarrow DAG\_PI4P}$	2.06E-03	2.30E-04	2.00E-02	5.16E-03	-6.24E-04	5.48E-02	1.64E-02	-5.50E-02	-3.11E-02	3.88E-02	-5.43E-02	-6.41E-02	-3.25E-02	-5.05E-02	-9.79E-02
$f_{45\rightarrow DAG\_PS}$	2.01E-01	2.36E-02	1.94E+00	4.97E-01	-6.02E-02	5.32E+00	1.59E+00	-5.01E+00	-2.86E+00	3.72E+00	-5.29E+00	-6.25E+00	-3.18E+00	-5.09E+00	-9.29E+00
$\gamma_{PA\rightarrow DAG}$	7.53E-04	2.61E-04	2.30E-03	-8.83E-04	-8.87E-05	-7.99E-03	1.59E-03	8.02E-03	4.53E-03	-5.66E-03	8.68E-03	-7.83E-03	-1.62E-02	7.34E-03	1.42E-02
$f_{PA\rightarrow DAG\_PA}$	6.82E-03	2.36E-03	2.09E-02	-7.99E-03	-8.03E-04	-7.23E-02	1.44E-02	7.26E-02	4.10E-02	-5.13E-02	7.85E-02	-7.09E-02	-1.47E-01	6.65E-02	1.29E-01
$\gamma_{DAG\rightarrow PA}$	-2.51E-02	-8.70E-03	-7.68E-02	2.94E-02	2.96E-03	2.66E-01	-5.29E-02	-2.66E-01	-1.50E-01	1.89E-01	-2.91E-01	2.61E-01	5.41E-01	-2.46E-01	-4.75E-01
$f_{DAG\rightarrow PA\_DAG}$	-2.06E-01	-7.12E-02	-6.32E-01	2.41E-01	2.44E-02	2.18E+00	-4.37E-01	-2.13E+00	-1.21E+00	1.54E+00	-2.46E+00	2.14E+00	4.49E+00	-2.05E+00	-3.89E+00
$\gamma_{\rightarrow DAG}$	5.49E-07	1.90E-07	1.68E-06	-6.43E-07	-6.46E-08	-5.82E-06	1.16E-06	5.76E-06	3.23E-06	-4.12E-06	-4.93E-06	-5.70E-06	-1.18E-05	-3.20E-06	-3.26E-06
$\gamma_{DAG\rightarrow}$	2.38E-02	8.25E-03	7.27E-02	-2.79E-02	-2.80E-03	-2.52E-01	5.01E-02	2.54E-01	1.43E-01	-1.79E-01	-7.19E-01	-2.47E-01	-5.12E-01	2.32E-01	4.50E-01
$f_{DAG\rightarrow\_DAG}$	2.06E-01	7.15E-02	6.26E-01	-2.42E-01	-2.41E-02	-2.18E+00	4.30E-01	2.25E+00	1.26E+00	-1.56E+00	-6.14E+00	-2.14E+00	-4.37E+00	1.95E+00	3.87E+00
$\gamma_{\rightarrow PA}$	-3.36E-04	-1.16E-04	-1.03E-03	3.94E-04	3.96E-05	3.56E-03	-7.08E-04	-3.57E-03	-2.02E-03	2.53E-03	3.03E-03	3.49E-03	7.23E-03	-3.29E-03	-6.37E-03
$\gamma_{PA\rightarrow}$	6.70E-02	2.32E-02	2.05E-01	-7.85E-02	-7.88E-03	-7.10E-01	1.41E-01	7.19E-01	4.05E-01	-5.04E-01	-6.03E-01	-6.96E-01	-1.44E+00	6.48E-01	1.27E+00
$f_{PA\rightarrow\_PA}$	5.80E-01	2.02E-01	1.75E+00	-6.83E-01	-6.74E-02	-6.14E+00	1.19E+00	6.68E+00	3.72E+00	-4.43E+00	-5.21E+00	-6.02E+00	-1.21E+01	5.08E+00	1.05E+01
$\gamma_{PC\rightarrow PA}$	-1.90E-02	-6.59E-03	-5.82E-02	2.23E-02	2.24E-03	2.02E-01	-4.01E-02	-2.02E-01	-1.14E-01	1.43E-01	1.71E-01	1.98E-01	4.10E-01	-1.86E-01	-3.60E-01
$f_{PC\rightarrow PA\_PI45P2}$	-5.31E-02	-1.84E-02	-1.63E-01	6.22E-02	6.27E-03	5.63E-01	-1.12E-01	-5.61E-01	-3.17E-01	3.99E-01	4.79E-01	5.52E-01	1.15E+00	-5.22E-01	-1.01E+00
$\gamma_{IP3\rightarrow}$	5.49E-07	1.90E-07	1.68E-06	-6.43E-07	-6.47E-08	-5.82E-06	1.16E-06	5.77E-06	3.23E-06	-4.13E-06	-4.95E-06	-9.90E-01	-1.18E-05	-3.19E-06	-3.26E-06
$f_{IP3\rightarrow\_IP3}$	5.49E-07	1.90E-07	1.68E-06	-6.43E-07	-6.47E-08	-5.82E-06	1.16E-06	5.77E-06	3.23E-06	-4.13E-06	-4.95E-06	-5.97E+00	-1.18E-05	-3.19E-06	-3.26E-06
$\gamma_{4\rightarrow DAG}$	-1.03E-02	2.55E-03	-1.45E-01	1.20E-02	7.22E-03	5.29E-02	-1.26E-01	-5.30E-02	-2.99E-02	3.75E-02	1.63E-01	5.35E-02	1.16E-01	-4.87E-02	-9.45E-02
$f_{4\rightarrow DAG\_PI4P}$	-9.78E-02	2.48E-02	-1.37E+00	1.14E-01	6.90E-02	5.00E-01	-1.20E+00	-4.98E-01	-2.81E-01	3.54E-01	1.55E+00	5.06E-01	1.10E+00	-4.63E-01	-8.92E-01
$f_{4\rightarrow DAG\_PI45P2}$	2.18E-03	-5.38E-04	3.05E-02	-2.53E-03	-1.52E-03	-1.12E-02	2.67E-02	1.12E-02	6.34E-03	-7.93E-03	-3.45E-02	-1.13E-02	-2.45E-02	1.03E-02	2.00E-02
$f_{4\rightarrow DAG\_PS}$	1.15E-01	-2.73E-02	1.60E+00	-1.32E-01	-7.89E-02	-5.89E-01	1.39E+00	5.96E-01	3.36E-01	-4.19E-01	-1.81E+00	-5.96E-01	-1.28E+00	5.38E-01	1.05E+00
SPLUNC1	5.49E-07	1.90E-07	1.68E-06	-6.43E-07	-6.47E-08	-5.82E-06	1.16E-06	5.77E-06	3.23E-06	-4.13E-06	-4.95E-06	-5.71E-06	-1.18E-05	-4.52E-01	1.23E-01
γ1	5.49E-07	1.90E-07	1.68E-06	-6.43E-07	-6.47E-08	-5.82E-06	1.16E-06	5.77E-06	3.23E-06	-4.13E-06	-4.95E-06	-5.71E-06	-1.18E-05	8.17E-01	-2.21E-01
γ2	5.49E-07	1.90E-07	1.68E-06	-6.43E-07	-6.47E-08	-5.82E-06	1.16E-06	5.77E-06	3.23E-06	-4.13E-06	-4.95E-06	-5.71E-06	-1.18E-05	-3.62E-01	9.81E-02
γ3	5.49E-07	1.90E-07	1.68E-06	-6.43E-07	-6.47E-08	-5.82E-06	1.16E-06	5.77E-06	3.23E-06	-4.13E-06	-4.95E-06	-5.71E-06	-1.18E-05	-4.52E-01	1.23E-01
γ4	5.49E-07	1.90E-07	1.68E-06	-6.43E-07	-6.47E-08	-5.82E-06	1.16E-06	5.77E-06	3.23E-06	-4.13E-06	-4.95E-06	-5.71E-06	-1.18E-05	3.54E-01	6.89E-01
γ5	5.49E-07	1.90E-07	1.68E-06	-6.43E-07	-6.47E-08	-5.82E-06	1.16E-06	5.77E-06	3.23E-06	-4.13E-06	-4.95E-06	-5.71E-06	-1.18E-05	9.71E-02	1.88E-01
γ6	5.49E-07	1.90E-07	1.68E-06	-6.43E-07	-6.47E-08	-5.82E-06	1.16E-06	5.77E-06	3.23E-06	-4.13E-06	-4.95E-06	-5.71E-06	-1.18E-05	-1.23E-01	-2.38E-01
γ7	5.49E-07	1.90E-07	1.68E-06	-6.43E-07	-6.47E-08	-5.82E-06	1.16E-06	5.77E-06	3.23E-06	-4.13E-06	-4.95E-06	-5.71E-06	-1.18E-05	-3.29E-01	-6.35E-01

Colored cell represents high sensitivities (variations of the dependent variables that are higher in absolute value to the perturbation in the parameters, which was 1%). Red and blue signal positive and negative high sensitivities respectively. Values in the box at the lower right corner are for sensitivities for ENaC and ASL to variables from the Olivença *et al.* (2019) [16] and are similar to the ones presented the first two columns in Table 3 of this paper.

References

1. Olivença, D. V. *et al.* A mathematical model of the phosphoinositide pathway. *Sci. Rep.* **8**, 3904 (2018).

2. Falkenburger, B. H., Dickson, E. J. & Hille, B. Quantitative properties and receptor reserve of the DAG and PKC branch of Gq-coupled receptor signaling. *J Gen Physiol* **141**, 537–555 (2013).

3. Sampaio, J. L. *et al.* Membrane lipidome of an epithelial cell line. *Proc. Natl. Acad. Sci. U. S. A.* **108**, 1903–1907 (2011).

4. Sciorra, V. A. *et al.* Dual role for phosphoinositides in regulation of yeast and mammalian phospholipase D enzymes. *J. Cell Biol.* **159**, 1039–1049 (2002).

5. Hodgkin, M. N. *et al.* Phospholipase D regulation and localisation is dependent upon a phosphatidylinositol 4, 5-bisphosphate-specific PH domain. *Curr. Biol.* **10**, 43–46 (2000).

6. Moritz, A., De Graan, P. N., Gispen, W. H. & Wirtz, K. W. Phosphatidic acid is a specific activator of phosphatidylinositol-4-phosphate kinase. *J. Biol. Chem.* **267**, 7207–7210 (1992).

7. Jenkins, G. H., Fisette, P. L. & Anderson, R. A. Type I phosphatidylinositol 4-phosphate 5-kinase isoforms are specifically stimulated by phosphatidic acid. *J. Biol. Chem.* **269**, 11547–54 (1994).

8. Jarquin-Pardo, M., Fitzpatrick, A., Galiano, F. J., First, E. A. & Davis, J. N. Phosphatidic acid regulates the affinity of the murine phosphatidylinositol 4-phosphate 5-kinase-1β for phosphatidylinositol-4-phosphate. *J. Cell. Biochem.* **100**, 112–128 (2007).

9. Sasaki, T. *et al.* Mammalian phosphoinositide kinases and phosphatases. *Prog. Lipid Res.* **48**, 307–343 (2009).

10. Feng, S. *et al.* A rapidly reversible chemical dimerizer system to study lipid signaling in living cells. *Angew. Chemie - Int. Ed.* **53**, 6720–6723 (2014).

11. Schomburg, I. *et al.* BRENDA: integrated reactions, kinetic data, enzyme function data, improved disease classification. Retrieved 2015, from http://www.brenda-enzymes.org (2015).

12. Jenkins, G. H. & Subrahmanyam, G. Anderson, R. A. Purification and reconstitution of phosphatidylinositol 4-kinase from human erythrocytes. *Biochim Biophys Acta.* **1080**, 11–28 (1991).

13. Jones, D. R., Sanjuan, M. A. & Mérida, I. Type I alpha phosphatidylinositol 4-phosphate 5-kinase is a putative target for increased intracellular phosphatidic acid. *FEBS Lett.* **476**, 160–165 (2000).

14. Purvis, J. E., Chatterjee, M. S., Brass, L. F. & Diamond, S. L. A molecular signaling model of platelet phosphoinositide and calcium regulation during homeostasis and P2Y1 activation. *Blood* **112**, 4069–4079 (2008).

15. Pochynyuk, O., Bugaj, V., Vandewalle, A. & Stockand, J. D. Purinergic control of apical plasma membrane PI(4,5)P2 levels sets ENaC activity in principal cells. *Am. J. Physiol. Renal Physiol.* **294**, F38-46 (2008).

16. Olivença, D. V., Fonseca, L. L., Voit, E. O. & Pinto, F. R. Thickness of the airway surface liquid layer

in the lung is affected in cystic fibrosis by compromised synergistic regulation of the ENaC ion channel. *J. R. Soc. Interface* **16**, 20190187 (2019).

17. Brass, L. F. & Joseph, S. K. A role for inositol triphosphate in intracellular Ca2+ mobilization and granule secretion in platelets. *J. Biol. Chem.* **260**, 15172–15179 (1985).

18. James, S. R., Paterson, A., Harden, T. H. & Downes, C. P. Kinetic analysis of phospholipase C beta isoforms using phospholipid-detergent mixed micelles. *J. Biol. Chem.* **270**, 11872–11881 (1995).

19. Seifert, J. P. *et al.* RhoA activates purified phospholipase C-ε by a guanine nucleotide-dependent mechanism. *J. Biol. Chem.* **279**, 47992–47997 (2004).

20. Ginger, R. S. & Parker, P. J. Expression, purification and characterisation of a functional phosphatidylinositol-specific phospholipase C-delta 1 protein in Escherichia coli. *Eur. J. Biochem.* **210**, 155–60 (1992).

21. Xu, C., Watras, J. & Loew, L. M. Kinetic analysis of receptor-activated phosphoinositide turnover. *J. Cell Biol.* **161**, 779–791 (2003).

22. Gericke, A., Leslie, N. R., Lösche, M. & Ross, A. H. PtdIns(4,5)P2-mediated cell signaling: Emerging principles and PTEN as a paradigm for regulatory mechanism. *Adv. Exp. Med. Biol.* **991**, 85–104 (2013).

23. Dickson, E. J., Falkenburger, B. H., Hille, B., Bertil, H. & Hille, B. Quantitative properties and receptor reserve of the IP3 and calcium branch of Gq-coupled receptor signaling. *J Gen Physiol* **141**, 521–535 (2013).

24. Chang, C.-L. & Liou, J. Phosphatidylinositol 4,5-Bisphosphate Homeostasis Regulated by Nir2 and Nir3 Proteins at Endoplasmic Reticulum-Plasma Membrane Junctions. *J. Biol. Chem.* **290**, 14289–14301 (2015).

25. Antonescu, C. N., Danuser, G. & Schmid, S. L. Phosphatidic acid plays a regulatory role in clathrin-mediated endocytosis. *Mol. Biol. Cell* **21**, 2944–2952 (2010).

26. Várnai, P. *et al.* Quantifying lipid changes in various membrane compartments using lipid binding protein domains. *Cell Calcium* **64**, 72–82 (2017).

27. Zhong, S. *et al.* Allosteric activation of the phosphoinositide phosphatase Sac1 by anionic phospholipids. *Biochemistry* **51**, 3170–3177 (2012).

28. Pochynyuk, O., Bugaj, V. & Stockand, J. D. Physiologic regulation of the epithelial sodium channel by phosphatidylinositides. *Curr. Opin. Nephrol. Hypertens.* **17**, 533–540 (2008).

29. McConnachie, G., Pass, I., Walker, S. M. & Downes, C. P. Interfacial kinetic analysis of the tumour suppressor phosphatase, PTEN: evidence for activation by anionic phospholipids. *Biochem. J.* **371**, 947–955 (2003).

30. Johnston, S. B. & Raines, R. T. Catalysis by the tumor-suppressor enzymes PTEN and PTEN-L. *PLoS One* **10**, 1–13 (2015).

31. Pochynyuk, O., Tong, Q., Staruschenko, A. & Stockand, J. D. Binding and direct activation of the epithelial Na+ channel (ENaC) by phosphatidylinositides. *J Physiol* **580**, 365–372 (2007).

32. Narang, A., Subramanian, K. K. & A., L. D. A mathematical model for chemoattractant gradient sensing based on receptor-regulated membrane phospholipid signaling dynamics. *Ann. Biomed. Eng.* **29**, 677–691 (2001).

33. Balla, T. Phosphoinositides: tiny lipids with giant impact on cell regulation. *Physiol. Rev.* **93**, 1019–1137 (2013).

34. Viaud, J. *et al.* Phosphoinositides: Important lipids in the coordination of cell dynamics. *Biochimie* **125**, 250–258 (2016).

35. Almaça, J. *et al.* High-content siRNA screen reveals global ENaC regulators and potential cystic fibrosis therapy targets. *Cell* **154**, 1390-1400 (2013).

36. Garcia-Caballero, A. *et al.* SPLUNC1 regulates airway surface liquid volume by protecting ENaC from proteolytic cleavage. *Proc. Natl. Acad. Sci.* **106**, 11412–11417 (2009).

37. Charalampous, F. C. Levels and distributions of phospholipids and cholesterol in the plasma membrane of neuroblastoma cells. *BBA - Biomembr.* **556**, 38–51 (1979).

38. Voit, E. O. *A first course in systems biology*. (Garland Science,Taylor & Francis Group, 2013).

39. Hammond, G. R. V. *et al.* PI4P and PI(4,5)P2 are essential but independent lipid determinants of membrane identity. *Science (80-.).* **337**, 727–730 (2012).

40. Yada, Y., Ozekis, T., Kanohlf, H. & Nozawasii, Y. Purification and Characterization of Human Platelets of Cytosolic Diacylglycerol Kinases. *Biochemistry* **265**, 19237–19243 (1990).

41. Wissing, J. B. & Wagner, K. G. Diacylglycerol kinase from suspension cultured plant cells : characterization and subcellular localization. *Plant Physiol* **98**, 1148–1153 (1992).

42. Takeuchi, M. *et al.* Cloning and characterization of DPPL1 and DPPL2, representatives of a novel type of mammalian phosphatidate phosphatase. *Gene* (2007) doi:10.1016/j.gene.2007.05.009.

43. English, D. *et al.* Characterization and purification of neutrophil ecto-phosphatidic acid phosphohydrolase. *Biochem J* **324 (Pt 3**, 941–950 (1997).
